# Gene-specific selective sweeps are pervasive across human gut microbiomes

**DOI:** 10.1038/s41586-025-09798-y

**Published:** 2025-12-17

**Authors:** Richard Wolff, Nandita R. Garud

**Affiliations:** 1 Department of Ecology and Evolutionary Biology, University of California Los Angeles, Los Angeles, CA USA; 2 Department of Human Genetics, University of California Los Angeles, Los Angeles, CA USA

**Keywords:** Population genetics, Microbial genetics

## Abstract

The human gut microbiome is composed of a highly diverse consortia of species that are continually evolving within and across hosts^1,2^. The ability to identify adaptations common to many human gut microbiomes would show not only shared selection pressures across hosts but also key drivers of functional differentiation of the microbiome that may affect community structure and host traits. However, the extent to which adaptations have spread across human gut microbiomes is relatively unknown. Here we develop a new selection scan statistic named the integrated linkage disequilibrium score (iLDS) that can detect sweeps of adaptive alleles spreading across host microbiomes by migration and horizontal gene transfer. Specifically, iLDS leverages signals of hitchhiking of deleterious variants with a beneficial variant. Application of the statistic to around 30 of the most prevalent commensal gut species from 24 human populations around the world showed more than 300 selective sweeps across species. We find an enrichment for selective sweeps at loci involved in carbohydrate metabolism, indicative of adaptation to host diet, and we find that the targets of selection differ significantly between industrialized populations and non-industrialized populations. One of these sweeps is at a locus known to be involved in the metabolism of maltodextrin—a synthetic starch that has recently become a widespread component of industrialized diets. In summary, our results indicate that recombination between strains fuels pervasive adaptive evolution among human gut commensal bacteria, and strongly implicate host diet and lifestyle as critical selection pressures.

## Abstract (web summary)

Development and application of the integrated linkage disequilibrium score (iLDS) reveals both selective pressures impacting the human gut microbiome and the mechanisms by which gut bacteria adapt to meet them.

## Main

The diverse species that make up the human gut microbiome evolve throughout the lifetimes of individual hosts and over longer timescales across many host colonization cycles. Recent work has shown that rapid evolution within hosts is common among commensal gut bacteria, with new mutations often arising and sweeping to high frequency in healthy adults over the course of days to months even in the absence of obvious perturbations such as antibiotics^1–5^. However, far less is known about the longer-term dynamics of adaptations as they spread across hosts.

A new adaptation first appearing in one host’s microbiome may potentially spread to many hosts through strain transmission and subsequent horizontal gene transfer (HGT). The human gut microbiome is known to be a hotspot for HGT^6–8^, allowing adaptive alleles to be recombined easily onto new genetic backgrounds. HGT has been shown to play a crucial role in the transmission of some genes, such as antibiotic resistance genes^9^, especially across species boundaries. However, the extent to which HGT facilitates the spread of adaptive alleles among strains of the same species of commensal gut microbiota, especially by homologous recombination, is unclear.

When an adaptive allele spreads in a population by means of a ‘gene-specific’ selective sweep, other nearby ‘hitchhiking’ variants, which may be neutral or even deleterious, will be transferred together with the adaptive variant. As a result, the same genomic sequence bearing both the adaptive allele and the hitchhikers will appear in otherwise distantly related strains present in different host microbiomes^6,10,11^. This local sequence sharing will result in a distinct signature of elevated linkage disequilibrium (LD)—a measure of the correlation between alleles at different positions—in the vicinity of the adaptive allele relative to the genomic background.

Whereas local elevations in LD have long been leveraged as a signature of selection in sexual eukaryotes^12–17^, LD-based scans for selection in bacteria have been limited so far^18^. One reason could be that the pervasiveness and dynamics of recombination in many species of bacteria, particularly gut commensal bacteria^1,6,19^, are just starting to emerge. Moreover, LD-based statistics can be confounded by other non-selective evolutionary forces, such as demographic contractions, which can also result in elevations in LD^20^. However, the understanding of how such forces operate among gut bacteria is still nascent^21^.

**Fig. 1:**
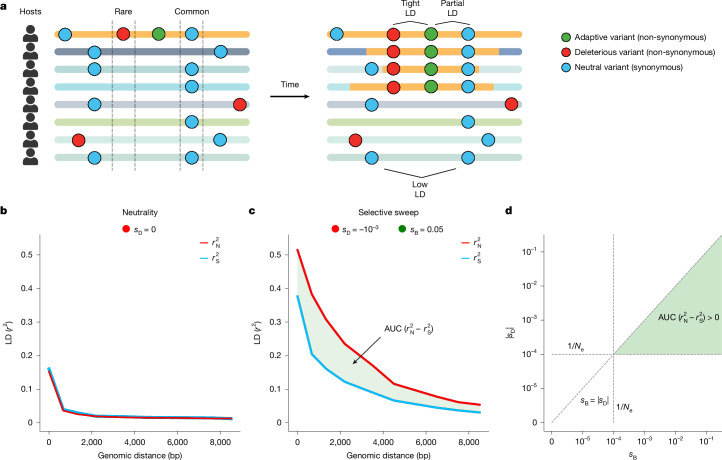
LD among common non-synonymous versus synonymous variants during a gene-specific selective sweep. **a**, During a gene-specific selective sweep, a genomic fragment bearing an adaptive variant transfers between strains. Here, each horizontal line represents a bacterial haplotype from a different host’s microbiome. The yellow region of each haplotype represents the fragment bearing the adaptive allele that has recombined onto different strains. **b**, ${r}_{{\rm{N}}}^{2}$ and ${r}_{{\rm{S}}}^{2}$ among common variants under neutrality. **c**, AUC$({r}_{{\rm{N}}}^{2}-{r}_{{\rm{S}}}^{2})$ among common variants for which purifying selection is of strength ${s}_{{\rm{D}}}={-10}^{-3}$ and beneficial selection is of strength ${s}_{{\rm{B}}}={10}^{-2}$. See Supplementary Figs. 1–3 for ${r}_{{\rm{N}}}^{2}$ and ${r}_{{\rm{S}}}^{2}$ measured across a comprehensive set of simulated evolutionary scenarios. **d**, AUC$({r}_{{\rm{N}}}^{2}-{r}_{{\rm{S}}}^{2})$ is expected to be greater than zero when *s*_B_ > *s*_D_ and both *s*_D_ and *s*_B_ are stronger than the effects of drift (1/*N*_e_, dashed lines). In this schematic and in all simulations (prior to a demographic contraction), *N*_e_ = 10^4^. See Supplementary Figs. 1–6 for ${r}_{{\rm{N}}}^{2}$ and ${r}_{{\rm{S}}}^{2}$ measured across a comprehensive set of simulated evolutionary scenarios.

We suggest that one way to detect recombination-mediated selective sweeps in bacteria while controlling for non-selective forces is to compare LD between non-synonymous versus synonymous variants. Specifically, we expect common non-synonymous variants to have higher LD than synonymous variants in the vicinity of adaptive loci that have swept to high frequency (Fig. 1a). Although both types of variant are subject to the same non-selective forces, synonymous variants are far more likely to be neutral. The vast majority of non-synonymous mutations, by contrast, are deleterious in any population^22^, and are thus generally rare^23,24^. However, initially rare non-synonymous variants in the vicinity of a new adaptive mutation may hitchhike to high frequency during a sweep^25^ and will therefore be found predominantly on haplotypes bearing the adaptive mutation. By contrast, synonymous variants can reach high frequency through neutral drift alone, in addition to hitchhiking during a sweep, and may be found both on sweeping and non-sweeping haplotypes. Thus, in the vicinity of a selective sweep, we expect that common high frequency non-synonymous variants will exhibit high LD with one another, while equally common synonymous variants will exhibit lower LD.

Here we confirm these hypotheses in simulations, and then show through application of our new statistic—the integrated LD score (iLDS)—to metagenomic data that recombination-mediated selective sweeps are pervasive in gut microbiota.

## Selection elevates non-synonymous LD

To test whether positive selection and hitchhiking of deleterious alleles can drive an excess of LD between pairs of non-synonymous variants versus pairs of synonymous variants and whether this signature is unique to selective sweeps or can arise due to non-selective forces, we performed forward population genetic simulations in SLiM 4.0 (ref. ^26^) (Supplementary Information section 2). We measured LD using the standard ${r}^{2}$ statistic (Supplementary Information section 1.1), and denote LD among non-synonymous variants ${r}_{{\rm{N}}}^{2}$ and LD among synonymous variants ${r}_{{\rm{S}}}^{2}$. In our simulations, we analyse LD among variants that are common (minor allele frequency (MAF) ≥ 0.2) in the broader population, enriching for positively selected alleles and hitchhikers. In recombining populations, more physically distant variants experience higher effective rates of recombination with one another and consequently have lower levels of LD, leading to a characteristic pattern of LD decay with distance. Therefore, to quantify whether ${r}_{{\rm{N}}}^{2}$ is elevated significantly over ${r}_{{\rm{S}}}^{2}$, we calculated the difference in area under their respective LD distance decay curves (AUC) (Fig. 1c). This test statistic, which we refer to as $\mathrm{AUC}({r}_{{\rm{N}}}^{2}-{r}_{{\rm{S}}}^{2})$, allows us to assess differences in total levels of ${r}_{{\rm{N}}}^{2}$ and ${r}_{{\rm{S}}}^{2}$ in a way that controls for genomic distance (and therefore effective recombination rates) between pairs of alleles (Fig. 1a and Supplementary Information section 1.2).

Before assessing whether selective sweeps produce $\mathrm{AUC}({r}_{{\rm{N}}}^{2}-{r}_{{\rm{S}}}^{2}) > 0$ among common variants, we first determined whether evolutionary scenarios lacking positive selection could generate this pattern. We found that this signature does not manifest under neutrality (Fig. 1b), as a result of purifying selection alone, demographic contractions or reductions in the recombination rate (Supplementary Figs. 1–3), or due to syntenic variability (Supplementary Fig. 27).

Next, we simulated an adaptive allele sweeping in a population in which all other non-synonymous mutations are deleterious with selection coefficient ${s}_{{\rm{D}}}$. First, regardless of ${s}_{{\rm{D}}}$, ${r}_{{\rm{N}}}^{2}$ and ${r}_{{\rm{S}}}^{2}$ generally increased monotonically with the strength of beneficial selection (${s}_{{\rm{B}}}$), reflecting the decrease in the expected time for the sweeping variant to reach intermediate frequency relative to neutrality. Second, we found that selective sweeps can in fact produce $\mathrm{AUC}({r}_{{\rm{N}}}^{2}-{r}_{{\rm{S}}}^{2}) > 0$; however, this pattern manifests only under particular combinations of ${s}_{{\rm{B}}}$ and ${s}_{{\rm{D}}}$. Specifically, the strength of purifying selection must exceed the effect of drift (${s}_{{\rm{D}}} > 1/{N}_{{\rm{e}}}$, where ${N}_{{\rm{e}}}$ is the effective population size), and the strength of positive selection must exceed that of purifying selection (${s}_{{\rm{B}}} > {s}_{{\rm{D}}}$) (Fig. 1d). In this regime, the bulk of non-synonymous variants are deleterious enough to be felt by selection and are therefore maintained at low frequency before the sweep, but are not so deleterious that they are unable to hitchhike. Thus, when a population experiences purifying and positive selection concurrently, we expect to see a systematic increase in ${r}_{{\rm{N}}}^{2}$ above ${r}_{{\rm{S}}}^{2}$ among common variants, in addition to an increase in LD overall.

## Elevated non-synonymous LD in commensals

Having established in simulations that selective sweeps can elevate ${r}_{{\rm{N}}}^{2}$ relative to ${r}_{{\rm{S}}}^{2}$ among common variants, we next quantified ${r}_{{\rm{N}}}^{2}$ and ${r}_{{\rm{S}}}^{2}$ in human gut commensal species to assess whether this signature of positive selection is observed at a genome-wide scale in natural populations. To do so, we analysed data from metagenomic samples of 693 people from North America, Europe and China^27–30^. To extract haplotypes from these data, we first aligned shotgun reads to a database of reference genomes using MIDAS^31^ (Supplementary Information section 3.1). We showed previously that, following read alignment, samples in which a single dominant strain of a species is present can be confidently ‘quasi-phased’ such that pairs of alleles can be assigned to the same haplotype with low probability of error^1^. Using these quasi-phased haplotypes, LD can be computed between pairs of alleles. In total, we extracted 3,316 quasi-phased haplotypes belonging to 32 species across the 693 people we examined. In subsequent sections of this work, we build further support for our findings made with quasi-phased haplotypes using a broader collection of metagenome assembled genomes (MAGs) and isolates collated from 24 populations around the world^32^.

Some of the species examined exhibit considerable population structure, with strong gene flow boundaries between clades. As LD can be sensitive to population structure, we focused our analyses only on haplotypes belonging to the largest clade of each species^1,6^ (Supplementary Information section 3.1.7).

**Fig. 2:**
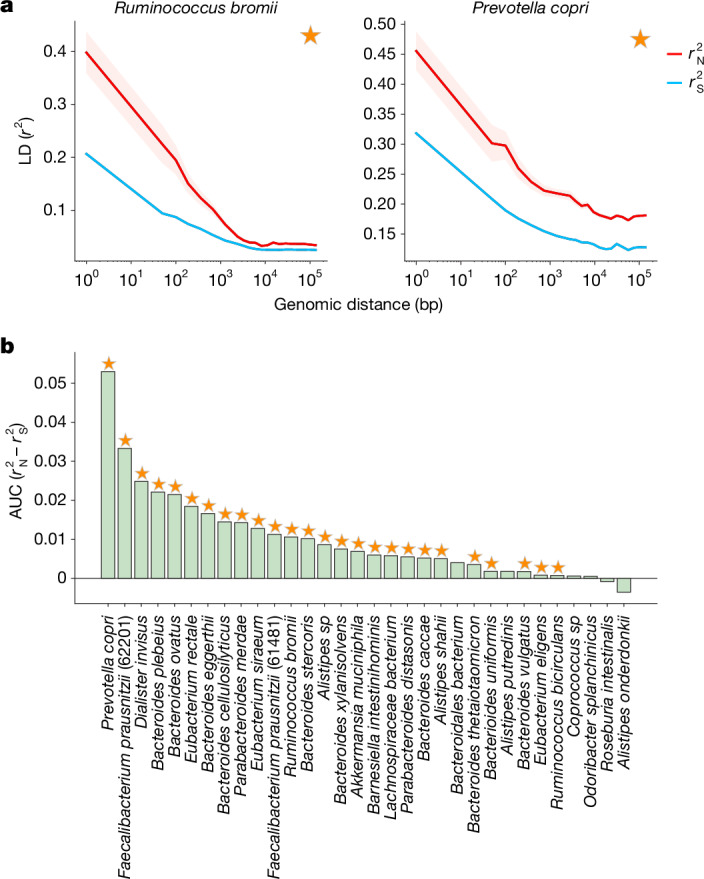
${{\boldsymbol{r}}}_{{\bf{N}}}^{{\bf{2}}}$ and ${{\boldsymbol{r}}}_{{\bf{S}}}^{{\bf{2}}}$ measured in prevalent commensal gut microbiota. **a**, Decay in LD among common ($\mathrm{MAF}\ge 0.2$) variants for the species *R. bromii* and *P. copri*. Both species show significant differences between ${r}_{{\rm{N}}}^{2}$ and ${r}_{{\rm{S}}}^{2}$ for common variants, as denoted by the star (confidence interval around ${r}_{{\rm{N}}}^{2}$ is shown by the red shaded region). For details on significance test, see Supplementary Information section 1.3. **b**, AUC(${r}_{{\rm{N}}}^{2}-{r}_{{\rm{S}}}^{2}$) among common variants for 32 prevalent gut commensal bacteria species. Stars at top indicate that AUC(${r}_{{\rm{N}}}^{2}-{r}_{{\rm{S}}}^{2}$) is significantly positive.

**Extended Data Fig. 1:**
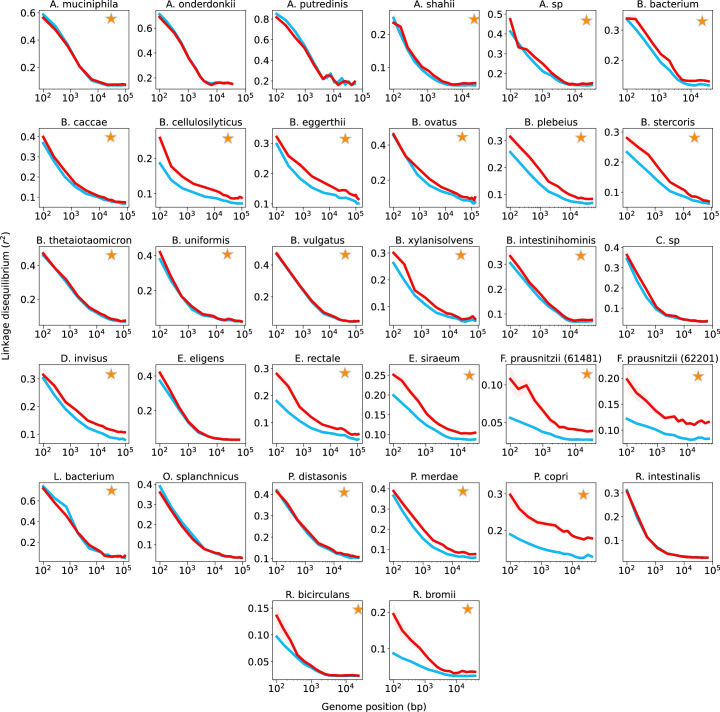
${{\boldsymbol{r}}}_{{\bf{N}}}^{{\bf{2}}}$ and ${{\boldsymbol{r}}}_{{\bf{S}}}^{{\bf{2}}}$ measured in prevalent commensal gut microbiota for common variants. This figure is analogous to Fig. 2a in the Main Text. ${r}_{N}^{2}$ is shown in red, while ${r}_{S}^{2}$ is shown in blue. Species in which AUC(${r}_{N}^{2}$ − ${r}_{S}^{2}$) is significantly greater than zero are denoted with a star. While ${r}_{N}^{2} < {r}_{S}^{2}$ at certain distances in some species (e.g. at short distances in *Alistipes putredinis*, overall ${r}_{N}^{2}$ typically exceeds ${r}_{S}^{2}$ for all species.

Shown in Fig. 2a are examples of genome-wide ${r}_{{\rm{N}}}^{2}$ and ${r}_{{\rm{S}}}^{2}$ for the species *Ruminococcus bromii* and *Prevotella copri*. For both species in Fig. 2a, $\mathrm{AUC}({r}_{{\rm{N}}}^{2}-{r}_{{\rm{S}}}^{2})$ is significantly greater than zero among common variants. More broadly, across the 32 species analysed, $\mathrm{AUC}({r}_{{\rm{N}}}^{2}-{r}_{{\rm{S}}}^{2})$ is significantly greater than zero among common variants in 26 of 32 species (Extended Data Fig. 1). Moreover, among rare variants (MAF $\le 0.05$), $\mathrm{AUC}({r}_{{\rm{N}}}^{2}-{r}_{{\rm{S}}}^{2})$ was significantly less than zero for most species, consistent with known effects of purifying selection (Supplementary Figs. 16 and 17). Together, these patterns of LD among synonymous and non-synonymous variants are consistent with widespread purifying and positive selection acting on non-synonymous sites in these species.

## Detecting selective sweeps with iLDS

To identify specific loci undergoing gene-specific sweeps, we developed the iLDS. This statistic detects genomic regions exhibiting both $\mathrm{AUC}({r}_{{\rm{N}}}^{2}-{r}_{{\rm{S}}}^{2}) > 0$ and elevated LD overall relative to the genomic background. By combining these sources of information, we identify regions of the genome where LD is elevated due to positive selection rather than other non-selective forces.

iLDS is calculated in sliding windows across a genome. Within each window, we first determine $\mathrm{AUC}({r}_{{\rm{N}}}^{2}-{r}_{{\rm{S}}}^{2})$ among common single-nucleotide variants ($\mathrm{MAF}\ge 0.2$). Next, to augment our ability to detect selection, we also identify windows with elevated LD relative to the genomic background, as expected for selective sweeps. To do so, we compute the difference in the area under the LD curve between all intermediate frequency variants in the window ($\mathrm{AUC}({r}_{\mathrm{local}}^{2})$), irrespective of whether they are synonymous or non-synonymous, and the area under the average genome-wide LD curve over the same distance defined by the window ($\mathrm{AUC}({r}_{\mathrm{genome} \mbox{-} \mathrm{wide}}^{2})$). The two components of iLDS are therefore:

$${r}_{\mathrm{\varDelta NS}}^{2}=\mathrm{AUC}({r}_{{\rm{N}}}^{2}-{r}_{{\rm{S}}}^{2})\,\mathrm{and}\,{r}_{\mathrm{\varDelta LG}}^{2}=\mathrm{AUC}({r}_{\mathrm{local}}^{2}-{r}_{\mathrm{genome}-\mathrm{wide}}^{2})$$

Next, to eventually focus on windows with outlier patterns of LD, each component is standardized by its mean and standard deviation across all windows along the genome:

$${\bar{r}}_{\Delta {\rm{N}}{\rm{S}}}^{2}=\frac{{r}_{\Delta {\rm{N}}{\rm{S}}}^{2}-E[{r}_{\Delta {\rm{N}}{\rm{S}}}^{2}]}{{\rm{s.d.}}({r}_{\Delta {\rm{N}}{\rm{S}}}^{2})}\,{\rm{a}}{\rm{n}}{\rm{d}}\,{\bar{r}}_{\Delta {\rm{L}}{\rm{G}}}^{2}=\frac{{r}_{\Delta {\rm{L}}{\rm{G}}}^{2}-E[{r}_{\Delta {\rm{L}}{\rm{G}}}^{2}]}{{\rm{s.d.}}({r}_{\Delta {\rm{L}}{\rm{G}}}^{2})}$$

Finally, the statistic is defined as:

$${\rm{i}}{\rm{L}}{\rm{D}}{\rm{S}}=({\bar{r}}_{\Delta {\rm{N}}{\rm{S}}}^{2}{)}^{2}+({\bar{r}}_{\Delta {\rm{L}}{\rm{G}}}^{2}{)}^{2}.$$

In essence, ${\bar{r}}_{\Delta {\rm{L}}{\rm{G}}}^{2}$ quantifies the increase in total LD within the window relative to the expected level of LD across the whole genomic background for a region of the same size, whereas ${\bar{r}}_{\Delta {\rm{N}}{\rm{S}}}^{2}$ quantifies the local extent of elevation in LD among non-synonymous variants relative to synonymous variants. Both terms are expected to be elevated during a sweep; however, iLDS should not be elevated in regions where ${r}_{\mathrm{local}}^{2}$ is high due to non-selective factors, as $\mathrm{AUC}({r}_{{\rm{N}}}^{2}-{r}_{{\rm{S}}}^{2})$ should remain near zero in such regions. To determine statistically whether a genomic window is under selection, we assess whether (1) both $\mathrm{AUC}({r}_{{\rm{N}}}^{2}-{r}_{{\rm{S}}}^{2})$ and $\mathrm{AUC}({r}_{\mathrm{local}}^{2}-{r}_{\mathrm{genome} \mbox{-} \mathrm{wide}}^{2})$ significantly exceed zero and (2) iLDS exceeds a critical threshold. For further details on this significance test, see Supplementary Information section 4.3.

## iLDS correctly identifies known sweeps

Before applying iLDS to gut commensals, we evaluated its ability to identify gene-specific selective sweeps first in simulations and then in empirical data from *Clostridiodes difficile*—a well-studied bacterial species known to have experienced recombination-mediated gene-specific sweeps at several loci.

Using simulations, we tested the ability of iLDS to correctly detect selective sweeps of varying strengths and ages since the cessation of selection (Supplementary Information section 4.4). We found that the statistic is powerful in detecting ongoing and recent, strong selective sweeps (Supplementary Fig. 7). Notably, given our stringent criteria for assessing significance, false positive rates remained below 1% under neutrality (Supplementary Fig. 11), indicating that iLDS is conservative in its selection inferences. In Supplementary Information section 4.4 we investigated the robustness of iLDS to a variety of evolutionary forces, including demographic contractions and variations in recombination rate and synteny, and found that none of these forces significantly elevated the false positive rate.

**Fig. 3:**
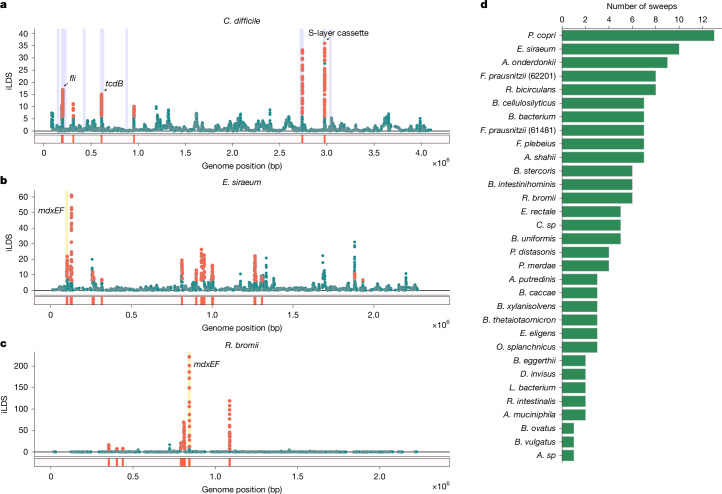
Gene-specific selective sweeps in gut bacteria. **a**, iLDS scan in *C. difficile*. Each point corresponds to an iLDS value for a given genomic window centred around a single intermediate frequency non-synonymous SNP. Significant windows are coloured orange, whereas non-significant windows are coloured green (see Supplementary Information section 4.3 for details of significance test). The locations of sweeps are shown as orange bars below the scan. Highlighted in blue are the locations of the genes predicted to be virulence factors^62^ (Supplementary Information section 3.2.1). iLDS is computed only within contigs. If several contigs are present, as in the case of *E. siraeum*, the scan is presented as a concatenation of results. **b**,**c**, iLDS scans for *E. siraeum* (**b**) and *R. bromii* (**c**). Both species have a sweep at the genes *mdxEF*, highlighted in yellow, as well as nine other loci in *E. siraeum* and five other loci in *R. bromii*. **d**, Number of selective sweeps detected per species.

To probe its performance in real data, we next applied the statistic to a set of 135 isolates of *C. difficile* (Fig. 3a)—an enteric pathogen. *C. difficile* has experienced recombination-mediated partial selective sweeps at several loci, including the *tcdB* locus encoding the toxin B virulence factor^33,34^ and the S-layer cassette^35,36^. We therefore sought to determine whether iLDS could correctly identify these loci as under selection.

To identify specific genomic loci that are under selection, we calculated iLDS in sliding windows across the genome, where the size of the windows (in base pairs) is approximately matched to the distance at which LD decays genome-wide (see Supplementary Information section 4.1 for further details), as such a window definition would allow us to then detect regions of elevated LD due to selection. In *C. difficile*, iLDS remains close to zero in most windows, as expected in the absence of positive selection. However, the value of the statistic peaks sharply in several regions across the genome (Fig. 3a), as expected following a selective sweep (Supplementary Information section 4.5).

**Extended Data Fig. 2:**
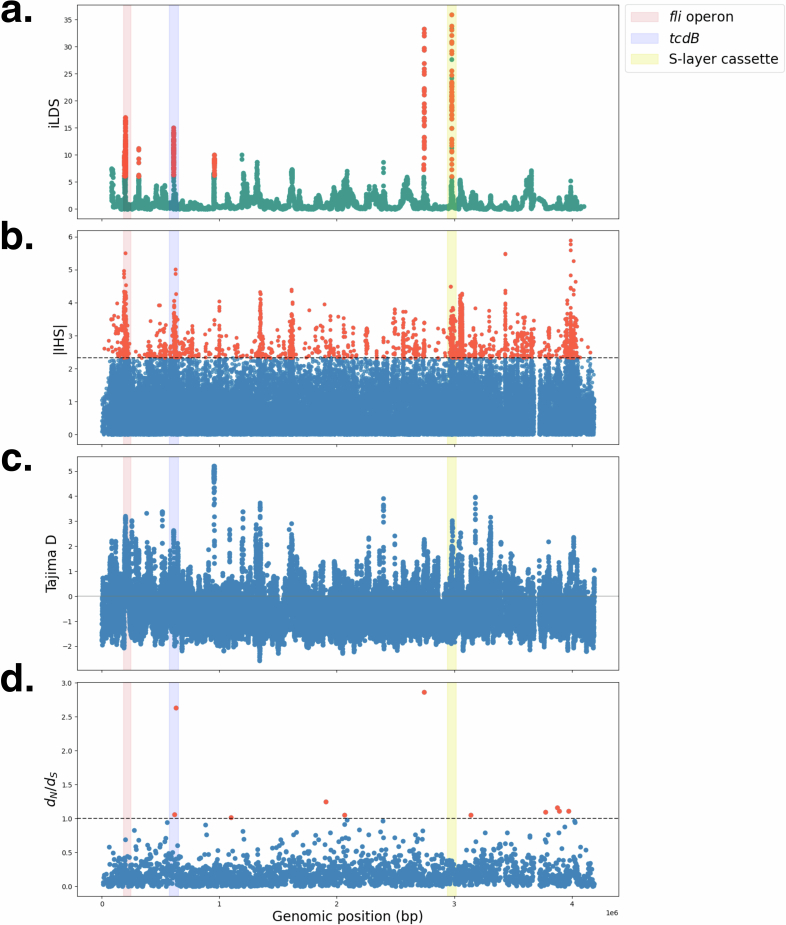
Comparison of iLDS with other selection scans run on *C. difficile.* 135 isolates of *C. difficile* were analyzed here in four different scans. (**a**) iLDS. Colored in orange are windows passing significance criteria for iLDS. (**b**) iHS. Colored in orange are windows with iHS values laying in the 99th percentile of the normal distribution ($\approx 2.32$). (**c**) Tajima’s D calculated in 1 kb windows, spaced by 100 bp. (**d**) ${d}_{N}/{d}_{S}$ calculated per gene. Colored in orange are genes with values lying above one. Highlighted are the positions of three loci previously shown (*tcdB* and S-layer cassette) or hypothesized (fli) to be under positive selection. Further details for the implementation of each statistic can be found in Supplementary Section 7.

In total, we identified six putative selective sweeps in *C. difficile*. Two of these sweeps overlap *tcdB* and the S-layer cassette, confirming that iLDS can indeed sensitively recover known instances of positive selection (Fig. 3a). Moreover, we found that iLDS visually exhibits improved resolution to detect these positive controls relative to other selection scan statistics (Extended Data Fig. 2).

Beyond these positive controls, we found a striking correspondence between the locations of putative sweeps inferred by iLDS and known virulence factors in the *C. difficile* genome (Fig. 3a, blue bars; Supplementary Information section 3.2.1). For instance, one sweep overlaps the *fli* operon—a virulence factor involved in flagellar biosynthesis that has been suggested previously to be under positive selection^36^. In total, out of eight predicted *C. difficile* virulence factors, four overlap or are near iLDS predicted sweeps, potentially indicating that positive selection on virulence-associated traits is an important facet of *C. difficile* evolution. In Supplementary Information section 9, we use iLDS to further investigate clade-specific selection pressures in *C. difficile*, finding evidence for selective sweeps spreading adaptive fragments both within and between clades.

**Extended Data Fig. 3:**
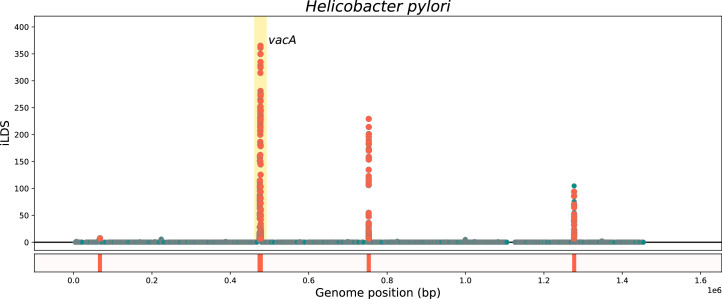
iLDS scan of *H. pylori.* iLDS scan for the highly recombinant gastric pathogen *Helicobacter pylori*. Much like *C. difficile* (Fig. 3a Main Text), iLDS detects that a key virulence factor (here, the *vacA* gene, which encodes the vacuolating cytotoxin) is under positive selection in this species. Previous studies have likewise reported that vacA is experiencing positive selection^66^. *H. pylori* isolate genomes were downloaded from UHGG.

**Extended Data Fig. 4:**
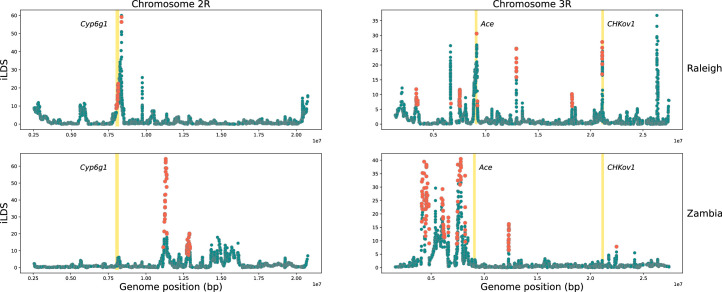
iLDS scans in *D. melanogaster.* iLDS scans for chromosomes 2 R (left) and 3 R (right) of *D. melanogaster* in the Raleigh (top) and Zambia (bottom) populations. ${l}_{{DD}}$ was set at 300 kb (Figure S31). The locations of the yellow vertical lines correspond to the location of known selective sweeps in the Raleigh population at the *Cyp6g1* (2 R) and *Ace* and *Chkov1* (3 R) loci plus 50 kb to either side of the locations. Significant windows are shown in orange, while non-significant windows are shown in green. For further details on our analyses with *D. melanogaster*, see Supplementary Section 10.

Further confirming the ability of iLDS to uncover selection in diverse natural populations, we found that iLDS is able to sensitively recover positive controls in two more well-studied species, *Helicobacter pylori* (Extended Data Fig. 3) and *Drosophila melanogaster* (Supplementary Information section 10 and Extended Data Fig. 4), underscoring its effectiveness in organisms of any ploidy, provided the species recombines.

## Pervasive selective sweeps in commensals

**Extended Data Fig. 5:**
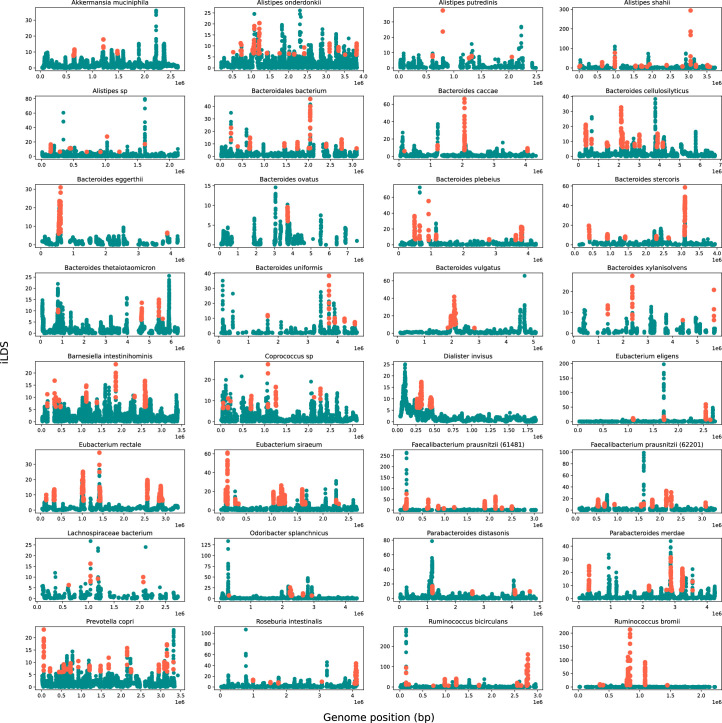
iLDS scans for all species analyzed (quasi-phased genomes). Analogous to Fig. 3b,c, iLDS scans are shown here for all 32 species whose genomes we quasi-phased. Significant windows are shown in orange, while non-significant windows are shown in green.

**Extended Data Fig. 6:**
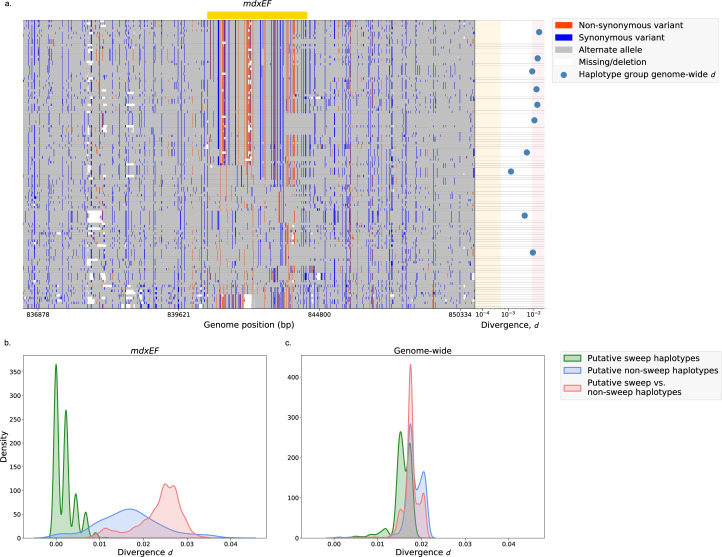
Genomic diversity at the *mdxEF* locus versus genome-wide for the species *R. bromii.* (**a**) Haplotype plot of a $$\sim $$10 kb region surrounding the sweep candidate *mdxEF* genes. Non-synonymous variants are colored red, while synonymous variants are colored blue, and missing sites are colored white. Horizontal lines separate strains into haplotype groups that are genetically identical at *mdxEF*. Haplotypes are ordered based on their genetic distance to the largest haplotype at the *mdxEF* locus. To aid in visualization, intermediate frequency variants (MAF $$ > 0.2$$) are colored based on the identity of the first strain in the first haplotype group (i.e. the top row), while the color of all other sites is determined by minor allele at that position. The alternate allele at each site assigned by this polarization scheme is colored gray. At right, mean genome-wide divergence $$d$$ within each haplotype group containing three or more strains is shown with blue dots, while the pink region shows the typical range of $$d$$ (within a factor of two of the mean), and the orange region denotes the region $$d < 5\times {10}^{-4}$$/bp, indicative of close-relatedness among strains. (**b**) For the species *R. bromii*, divergence $$d$$ at 4D sites is shown at *mdxEF* (left) and genome-wide (right). The distribution of $$d$$ among samples is shown for the putatively sweeping haplotype group in green (the 26 haplotype groups at the top in (**a**), above), for the remaining haplotype groups in blue, and between these two groups in red. At *mdxEF*, the putatively sweeping haplotype groups have very low $$d$$ relative to their genome-wide average, while the remaining haplotype groups have $$d$$ comparable to their genome-wide average (though with greater variance, as is expected given that $$d$$ is calculated over far fewer sites). Moreover, the putatively sweeping haplotypes are far more genetically distinct from the remaining haplotypes at *mdxEF* than genome-wide.

Next, we applied iLDS to the 32 gut microbiome species analysed in Fig. 2b. We identified a total of 155 unique sweeps across all species, with a median of four sweeps per species (Fig. 3d). In total, these sweeps spanned 447 genes (Extended Data Fig. 5 and Supplementary Table 4). While these genes were functionally diverse, we found certain classes of genes repeatedly under selection. For example, we identified five instances in five unique species of sweeps spanning *susC*/*susD* starch utilization system genes (Supplementary Fig. 20, which have been found previously to be under selection within several independent hosts over weeks to months^2,37^. Among all 447 genes overlapping iLDS predicted sweeps, we observed an enrichment for carbohydrate transport and metabolism genes at an $\alpha =0.05$ threshold after correcting for several hypotheses (clusters of orthologous groups category G^38^; Fisher exact test, adjusted single-sided *P* value $< 5\times {10}^{-7}$; Supplementary Information section 6 and Extended Data Fig. 6). We also saw that specific classes of enzymes involved in carbohydrate metabolism were enriched among genes under selection, in particular glycoside hydrolases^39^ (EC number 3.2.1; Fisher exact test, adjusted single-sided *P* value $=\,0.02$). These enrichment results provide evidence that genes related to the breakdown and transport of carbohydrates are targeted frequently by selection in the gut microbiome.

**Extended Data Fig. 7:**
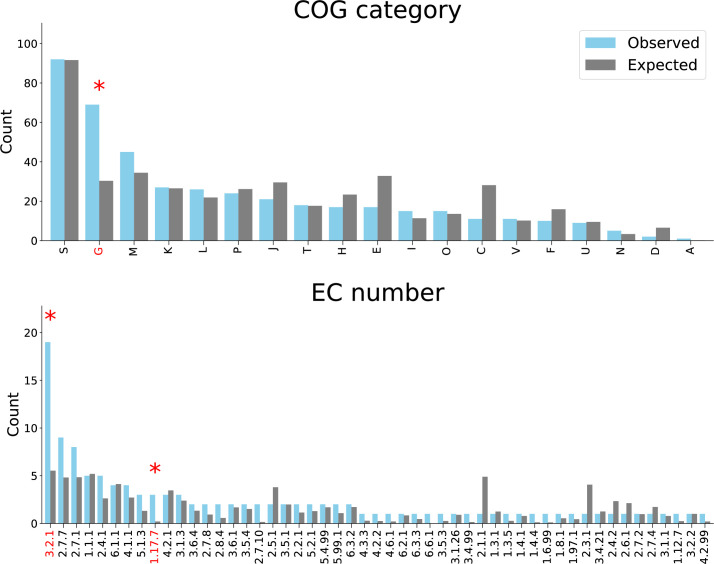
Enrichment of functional categories among sweeps. Number of genes expected to be under selection, based on the frequency of such genes across the core genomes of the 32 species analyzed, versus the number of genes observed to be under selection for two kinds of functional annotations: COG categories (top) and Enzyme Commission (EC) numbers (bottom). Functional categories exhibiting a statistically significant enrichment among selected genes are denoted by red asterisks and labels. Among COG categories (top), we observed a statistically significant enrichment of genes related to carbohydrate metabolism and transport (category G). Among ECs, we observed an enrichment of glycoside hydrolases (EC 3.2.1) and oxidoreductases using ferredoxin as an electron acceptor (EC 1.17.7). For further information on our gene enrichment analysis, including details of significance test, see Supplementary Section 6.

One particular set of carbohydrate metabolism genes detected repeatedly as under selection were *mdxE* and *mdxF*, which are ABC transporters capable of metabolizing maltodextrin^40^—a starch derivative commonly used as an emulsifier and textural component of ultra-processed foods^41^. The genes *mdxEF* are present in only four unique species in our dataset, but are identified as under selection by iLDS in two of these species: *Eubacterium siraeum*and *R. bromii* (Fig. 3b,c), both known to metabolize starches in the colon^42,43^. By inspecting the haplotypes at and surrounding *mdxEF*, we see that this putatively adaptive region exhibits evidence of extensive, recent HGT resembling a selective sweep (Extended Data Fig. 7).

## Spread of adaptations around the world

The shift from traditional to industrialized lifestyle has reshaped the gut microbiome, altering its ecological composition and causing an overall reduction in diversity^44^. Previous work has demonstrated that the shift to industrialization has also altered evolutionary trajectories within species, with industrialization driving elevated rates of HGT^8^, as well as differentiation in the pool of mobile genetic elements^45^. We hypothesized that the targets of adaptation in industrialized and non-industrialized microbiota are distinct as a consequence of selective pressures specific to each group.

**Fig. 4:**
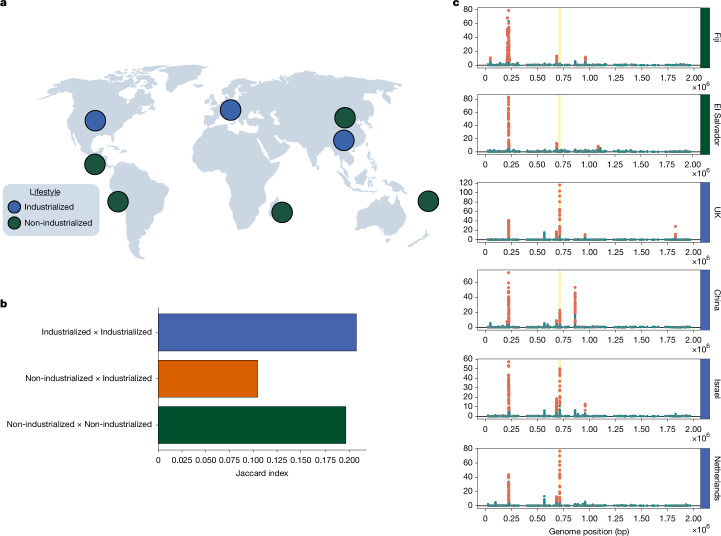
Selective sweeps across continents and lifestyles. **a**, Locations of industrialized and non-industrialized populations analysed. **b**, Overlap in the locations of sweeps between industrialized and non-industrialized populations, as determined by the Jaccard index (Supplementary Information section 5.2) **c**, Selective sweeps in *R. bromii* in two non-industrialized populations and four industrialized populations from around the world. The *mdxEF* genes are highlighted in gold. For the full set of scans across all 16 populations analysed, see Extended Data Fig. 8. World map in **a** created by Pixabay and reproduced from Canva (https://www.canva.com/) under a Free Content licence.

To test this hypothesis, we performed iLDS scans in isolates and MAGs from the Unified Human Gastrointestinal Genome (UHGG) catalogue^32^. We performed this analysis for 16 species present in healthy hosts from industrialized populations in Europe, Asia and North America, as well as in those from non-industrialized populations from Fiji^45^, Madagascar^46^, Peru^47^, El Salvador^48^ and Mongolia^49^ (Fig. 4a). In total, we analysed 24 populations around the world (19 industrialized, five non-industrialized; Supplementary Information section 3.2.2).

Application of iLDS to the UHGG dataset revealed a total of 309 unique selective sweeps across the 24 populations and 16 species studied (Supplementary Table 4). Most (201) of sweeps were unique to a single population, suggesting potentially local adaptation to regional diets and lifestyles.

Although there are many sweeps unique to each population, 35% were shared between populations, with 108 sweeps replicating in more than one country and 83 on several continents (Supplementary Fig. 24). Some sweeps were distributed extremely broadly, with 26 sweeps present in 50% or more of the populations analysed and eight present in 80% or more. In total, we found that 25 sweeps in 11 species had spread to a significantly greater number of subpopulations than expected by chance under a null model in which sweeps were permuted randomly among subpopulations (Supplementary Fig. 25). Overall, we found evidence for selective sweeps that have spread across the world.

Next, we assessed whether sweeps were shared more commonly between industrialized populations than between industrialized versus non-industrialized. To do so, we calculated a Jaccard index ($$J$$) to quantify the proportion of sweeps shared between populations (Supplementary Information section 5.2). Consistent with our hypothesis, we found that industrialized populations share sweeps with one another at more than double the frequency ($$J=0.21$$, *P* value $$=\,0.047$$, permutation test) than they do with non-industrialized populations ($$J=0.11$$, *P* value $$ < {10}^{-4}$$) (Fig. 4b and Supplementary Fig. 26). Similarly, non-industrialized populations also shared sweeps with one another ($$J=0.18$$, *P* value $$=\,0.67$$) more frequently than with industrialized populations, though this elevated sharing was not statistically significant. Together, these results indicate that selection pressures shared across industrialized populations may drive evolutionary differentiation from non-industrialized populations through selective sweeps.

Beyond the evident aggregate difference in sweeps between industrialized and non-industrialized populations, we also identified specific selective sweeps that were unique to one group or the other. In total, we identified 32 sweeps present in 50% or more of populations of one type (industrialized or non-industrialized) but absent in populations of the other type. Of these, 24 were unique to industrialized populations and eight to non-industrialized populations. In contrast, only three sweeps were found to be present in $\ge 50 \%$ of both industrialized and non-industrialized populations, underscoring the lack of shared selective pressures between these populations.

**Extended Data Fig. 8:**
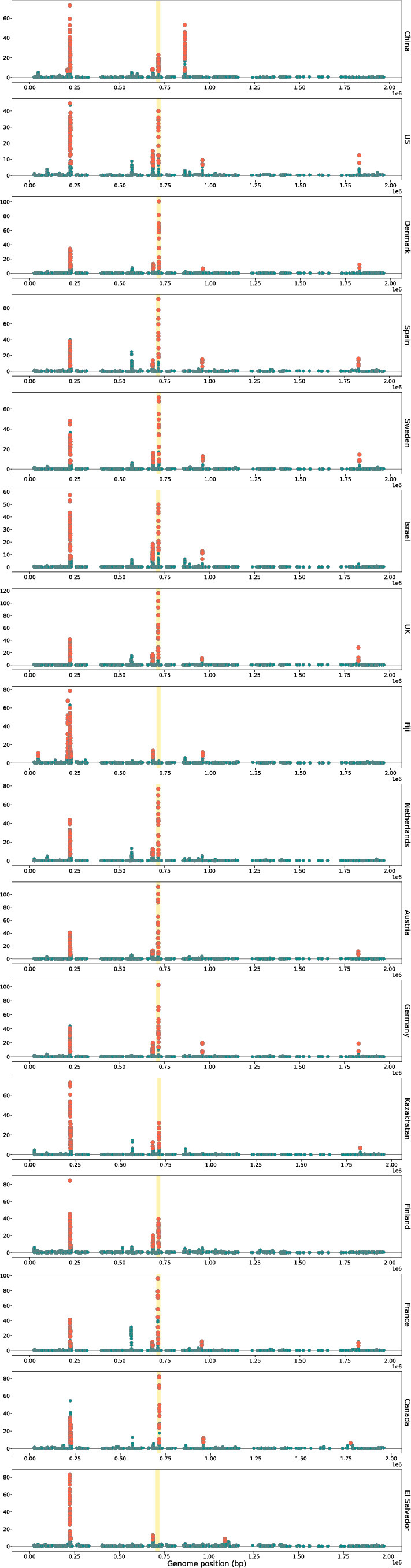
*R. bromii* scans across 16 populations. Expanding on Fig. 4c of the Main Text, iLDS scans for all populations of *R. bromii* present in UHGG. As noted in the Main Text, both non-industrialized populations (Fiji and El Salvador—bottom) lack the sweep at the *mdxEF* locus (highlighted in gold), which is present in 14/14 industrialized populations. Note that the order of the contigs present in the reference genome of *R. bromii* in UHGG differed from that of the reference genome in the MIDAS database to which metagenomes were mapped for analyses with quasi-phased samples. For ease of visualization and compatibility with Main Text Fig. 3c, we have matched the ordering of the contigs in UHGG to that of the reference genome in the MIDAS database for these iLDS scans.

Replicating our analysis of quasi-phased samples, the *R. bromii mdxEF* locus discussed in the preceding section (Fig. 3c) was also detected to be under selection in several populations in the UHGG data (Extended Data Fig. 8) indicating the robustness of our results to data type. This locus was among the sweeps that exhibited the strongest pattern of differential selection between industrialized and non-industrialized populations. In fact, these genes were found to be under selection in all 14 industrialized populations but neither non-industrialized population in which the species was present in sufficient numbers (Fiji and El Salvador) (Fig. 4c), suggesting that this species may be specifically adapting to industrialized lifestyles.

Although some targets of selection may differ between industrialized and non-industrialized microbiota, we also found that the total number of selective sweeps per population were similar, indicating the gut microbiota of non-industrialized populations may be adapting at a similar rate to those of industrialized populations. Whereas we found industrialized populations tended to harbour slightly more sweeps (3.46 sweeps per population) than non-industrialized populations (3.25 sweeps per population), this difference was not statistically significant (permutation test, *P* value $=\,0.6$).

## Discussion

The discovery of hundreds of gene-specific selective sweeps suggests that recombination is a principal mechanism by which adaptive DNA spreads in human gut bacteria. Whereas previous work has found extensive transfer of DNA by HGT across species boundaries^7,8^ and also among strains of the same species^1,6^, we establish here that gene-specific selective sweeps are a pervasive feature of the evolution of human gut bacteria. We emphasize that the success of our new statistic iLDS depends critically on the fact that homologous recombination is ubiquitous in these species, allowing us to distinguish selected regions of the genome with elevated LD relative to the genomic background, where recombination rapidly unlinks variants. So far, LD-based scans for selection may have been employed infrequently in bacteria^18^ because the true pervasiveness of recombination in many species of bacteria, including commensal gut bacteria, has become clear only recently^1,6,50,51^.

Our work adds to a growing literature suggesting that host diet not only changes the species composition of the microbiome but also selects for specific genetic variants within species. Human populations that consume diets rich in seaweed glycans^52^, red meat^53^ and plant starches^45^ seem to select for genes in particular bacterial species that facilitate the metabolism of these substrates within the host. Our findings suggest that adaptations to host diet are distributed broadly across many pathways in many different species. Underscoring the role of diet driving adaptations in the gut microbiome, we found a selective sweep at the *mdxEF* locus in *R. bromii* that is ubiquitous in industrialized populations but absent from non-industrialized populations. Although the precise selection pressures driving the spread of *mdxEF* variants across industrialized populations are unclear, these genes are known to facilitate growth on maltodextrin—a synthetic starch derivative used frequently in ultra-processed foods for its emulsifying and textural properties^41^—raising the possibility that this selective sweep represents an adaptation to a new source of dietary starch in the industrialized diet. Future work investigating the functional and ecological consequences of the selective sweeps we have identified will be important to understanding the role of each genetic variant in the microbiome.

We note that others have also observed that elevated LD among non-synonymous variants relative to synonymous variants can be a signature of adaptation^54–57^; however, the connection with hitchhiking of deleterious alleles had not been noted previously. Stolyarova et al.^54^ and Callahan et al.^57^ found that epistatic interactions between non-synonymous variants could generate this signal, while Arnold et al.^55^ concluded that epistasis was not necessary, and that adaptive inter-specific HGT of short genomic fragments bearing several positively selected non-synonymous alleles was the likely driving factor. We emphasize that our findings are fully consistent with those of Stolyarova et al., Callahan et al. and Arnold et al. But, crucially, our results indicate that elevated LD among common non-synonymous variants is not by itself sufficient to establish that all such variants are adaptive or epistatically interacting. Because purifying selection at the vast majority of non-synonymous sites is well-established to be a pervasive feature not only of bacterial genomes^1,24,58,59^, but also in the genomes of most other species^22^, it is likely some proportion of common non-synonymous polymorphisms will be deleterious hitchhikers in any adapting population^60^, with this proportion growing, paradoxically, as the strength of positive selection increases. In future work, disentangling the effect of epistasis versus hitchhiking of deleterious alleles will be important for understanding the relative contributions of different population genetic forces driving selective sweeps in bacteria and other natural populations.

In conclusion, development and application of iLDS helps us to learn about both selective pressures impacting the gut microbiome and the mechanisms by which gut bacteria adapt to meet them. Although we have uncovered hundreds of selective sweeps, we chose to conservatively calibrate iLDS to achieve a low false positive rate; as a result, we probably also missed some proportion of true positives, and thus the pervasiveness of positive selection in gut commensals may remain to be fully realized. Future molecular studies investigating the functional importance of selected loci identified by iLDS may provide mechanistic insight into how microbiome genotypes confer phenotypes on hosts, improve our ability to diagnose and treat diseases associated with specific microbiome variants^61^ and allow us to deploy existing natural variation in the design of rational probiotics.

### Reporting summary

Further information on research design is available in the Nature Portfolio Reporting Summary linked to this article.

## Online content

Any methods, additional references, Nature Portfolio reporting summaries, source data, extended data, supplementary information, acknowledgements, peer review information; details of author contributions and competing interests; and statements of data and code availability are available at 10.1038/s41586-025-09798-y.

## Supplementary information

Supplementary Information A guide to Supplementary Tables 1–6 (tables supplied separately), Figs. 1–32 and Text—see Contents for details.

Reporting Summary

Supplementary Table 1 Metadata for metagenomic samples.

Supplementary Table 2 Clade definitions.

Supplementary Table 3 iLDS scan parameters.

Supplementary Table 4 Peaks.

Supplementary Table 5 Metadata for UHGG samples.

Supplementary Table 6 Accessions for *R. bromii* isolates.

## Data Availability

Accession codes for the metagenomic data analysed in this paper are as follows: PRJNA48479, PRJNA275349, PRJEB9576, PRJNA422434 and PRJEB24041. A full set of individual samples is included in Supplementary Table 1. For analyses involving UHGG data^32^, we downloaded alignments of MAGs and isolates, as well as accompanying genomic data files used to annotate sites and genes, from MGnify (https://ftp.ebi.ac.uk/pub/databases/metagenomics/mgnify_genomes/human-gut/). Their accessions are available in Supplementary Table 6. Finally, for *D. melanogaster* analyses, we used publicly available *Drosophila* Genome Nexus dataset^63^. These include 205 *Drosophila* Genetic Reference Panel (DPGP) strains from Raleigh, North Carolina and 197 DPGP3 strains from Zambia. These data can be downloaded at www.johnpool.net. Data used for figure generation are available at Dryad (https://datadryad.org/dataset/doi:10.5061/dryad.9ghx3ffx4)^64^. Data for figures are available at Zenodo (10.5281/zenodo.17253586)^65^.

## Code availability

Custom code was developed for the analyses performed in this paper. The code is available at Zenodo (10.5281/zenodo.17253586)^65^. The code is also available on GitHub: https://github.com/garudlab/iLDS.
