# Supplementary material for: Gene-specific selective sweeps are pervasive across human gut microbiomes
Source: Nature. 2025 Dec 17;650(8102):710–7. doi: 10.1038/s41586-025-09798-y (PMC12916291; doi:10.1038/s41586-025-09798-y)
Supplement: Supplementary file 1 — A guide to Supplementary Tables 1–6 (tables supplied separately), Figs. 1–32 and Text—see Contents for details. [file 41586_2025_9798_MOESM1_ESM.pdf]

nature portfolio

https://doi.org/10.1038/s41586-025-09798-y

Supplementary information

Gene-specific selective sweeps are pervasive across human gut microbiomes

In the format provided by the
authors and unedited

Supplementary materials for Wolff and Garud 2025: Gene-specific selective sweeps are pervasive across human gut microbiomes

SI Guide (Table of Contents)

List of Supplementary Tables

Table S1: Metadata for metagenomic samples. This table contains a list of the 1,013 shotgun metagenomic samples collected from 693 unique, healthy individuals which we used in this study, and were originally analyzed by Garud *et al.* (2019)[1]. This includes 250 individuals from Lloyd-Price (2017) (accession numbers PRJNA48479 and PRJNA275349)[2], 250 from Xie *et al.* (2016) (accession number PRJEB9576)[3], 185 from Qin *et al.* (2012) (accession number PRJNA422434)[4], and 8 from Korpela *et al.* (2018) (accession number PRJEB24041)[5]. For each sample used, the subject identifier, sample identifier, accession number, country of the study, continent of the study, and visit number are included, as well as the study from which the sample was drawn.

Table S2: Clade definitions. This table contains the top-level clades which were manually defined for this dataset previously in Garud *et al.* (2019)[1]. This table also contains the clades that were manually defined in this paper for the UHGG dataset[6]. Each row details a strain ID, clade label, species, and data type of each strain.

Table S3: iLDS scan parameters. This table shows the parameters used to perform the iLDS scans. In particular, for each species, it shows the tract length l_r, decay distance l_{DD}, window size, number of bins per window, and the total number of intermediate frequency variants non-synonymous variants present genome-wide. For further methodological details on how these quantities were obtained, see Supplementary Section 4.1.

Table S4: Peaks. This table provides the locations of iLDS peaks. The columns of the table are:

- **Gene**: the gene the variant lies in
- **Contig**: the contig the variant lies on (in the case of peaks for *Drosophila melanogaster*, this is the chromosome)
- **SNV position (contig)**: the position of the central intermediate-frequency non-synonymous SNP in the analysis window with the highest iLDS value along the given contig
- **Species**: Name of the species

- **Peak number** the unique peak to which the variant belongs (peaks are numbered from left to right for each species)
- **Gene product**: the gene protein product
- **iLDS**: value of iLDS for the variant
- **Country**: For UHGG and Drosophila, country where scan was run
- **Data type**: the dataset the scan was performed on (quasi-phased metagenomes, UHGG, or Drosophila)
- **COG**: Cluster of Orthologous Genes category[7]
- **EC**: Enzyme Commission number of the gene[8]

Table S5: Metadata for UHGG samples. Metadata for strains coming from UHGG used in this study. These sequences, and the metadata presented in this table, were downloaded from UHGG[6]. The SNV catalogs used here are available from the MGnify FTP site: http://ftp.ebi.ac.uk/pub/databases/metagenomics/mgnify_genomes/.

Table S6: Accessions for *R. bromii* isolates. Strain ID, accession numbers, and sampling locations for *R. bromii* isolates used in analysis of syntenic variation (Supplementary Section 8).

List of Figures

S1 r^2_N and r^2_S measured among common variants in a range of simulated evolutionary scenarios, where $\rho = 10^{-5}$ 7

S2 r^2_N and r^2_S measured among common variants in a range of simulated evolutionary scenarios, where $\rho = 10^{-6}$ 8

S3 r^2_N and r^2_S measured among common variants in a range of simulated evolutionary scenarios, where $\rho = 10^{-7}$ 9

S4 r^2_N and r^2_S measured among rare variants in a range of simulated evolutionary scenarios, where $\rho = 10^{-5}$ 10

S5 r^2_N and r^2_S measured among rare variants in a range of simulated evolutionary scenarios, where $\rho = 10^{-6}$ 11

S6 r^2_N and r^2_S measured among rare variants in a range of simulated evolutionary scenarios, where $\rho = 10^{-7}$ 12

S7 Power analysis for iLDS under various sweep scenarios versus a constant N_e population at mutation-selection balance 13

S8 Power analysis for iLDS under various sweep scenarios versus populations experiencing a sharp, short bottleneck under mutation-selection balance 14

S9 Power analysis for iLDS under various sweeps scenarios versus populations experiencing a long, shallow bottleneck 15

S10 Power analysis for $r^2_{\Delta NS}$, $r^2_{\Delta LG}$ and iLDS 16

S11 True positive, false positive, and false discovery rates for sweeps against constant N_e ... 17

S12 True positive, false positive, and false discovery rates for sweeps against a sharp, short bottleneck 18

S13 True positive, false positive, and false discovery rates for sweeps against a long, shallow bottleneck 19

S14 Number of quasi-phased samples by species 20

S15 ($r^2_N - r^2_S$) measured in prevalent commensal gut microbiota for common variants. . . 21

S16 r^2_N and r^2_S measured in prevalent commensal gut microbiota for rare (MAF $\leq$ 0.05) variants 22

S17 AUC($r^2_N - r^2_S$) measured in prevalent commensal gut microbiota for varying allele frequencies 23
S18 $\bar{r}^2_{\Delta NS}$ versus $\bar{r}^2_{\Delta LG}$ in simulations and data 24
S19 QQ plots of $\bar{r}^2_{\Delta NS}$ versus $\bar{r}^2_{\Delta LG}$ in simulations and data 25
S20 *susC*/*susD* genes under selection 26
S21 Estimates of l_{DD} for common commensal gut microbiota 27
S22 Schematic of method for inferring l_r and l_{DD} 28
S23 Comparison of l_r and l_{DD} estimates with those of Liu and Good (2024) 29
S24 Spread of sweeps across populations 30
S25 Presence, absence, and widespread sweeps 31
S26 Jaccard permutation test 32
S27 Impact of syntenic variation on $r^2_{\Delta NS}$ and $r^2_{\Delta LG}$ in simulated sweeps and neutrality. 33
S28 Distribution of N^{50}_{syn} for *R. bromii* isolates. 34
S29 Synteny of *R. bromii* isolates with the reference genome used in the MIDAS database. 35
S30 Comparison of iLDS scans run in *R. bromii* with vs without syntenic variation and across datasets. 36
S31 iLDS and *C. difficile* population structure. 37
S32 $r^2_{\Delta LG}$, $r^2_{\Delta NS}$, and iLDS as a function of recombination rate 38

Supplementary Text

1 LD 39

1.1 LD calculations 39

1.2 AUC 39

1.3 LD confidence intervals 39

2 Simulations 40

2.1 Burn-in/mutation-selection balance 41

2.2 Demographic contractions 42

2.3 Partial sweep 42

3 Data sources 42

3.1 Quasi-phased genomes 43

3.1.1 Estimation of species, gene, and SNV content of shotgun metagenomic samples 43

3.1.2 Estimation of species content 43

3.1.3 Estimation of CNV content 43

3.1.4 Estimation of SNV content 44

3.1.5 Quasi-phasing procedure 44

3.1.6 Inclusion criteria 45

3.1.7 Population structure 45

3.2 UHGG 45

3.2.1 Virulence factors 46

3.2.2 Identifying industrialized samples 46

4 iLDS 47

4.1 Determining the window size for computing iLDS 47

4.1.1 Comparison of tract length estimates with previous work 49

4.2 Application of iLDS to data 50

4.3 Identification of analysis windows with significant iLDS values 50
4.4 Evaluating the performance of iLDS in simulations 50
4.4.1 Selective sweeps 51
4.4.2 Demographic contractions 51
4.4.3 Calculating iLDS 52
4.4.4 Simulation results 52
4.5 Peak calling 53
5 Distribution and overlap of sweeps among populations 54
5.1 Detecting widespread sweeps 54
5.2 Jaccard index 55
6 Gene enrichment analysis 55
7 Comparison of iLDS with previous selection scans in data 56
8 Robustness of iLDS to synteny 56
9 iLDS performance in structured populations 58
10 Analyses with Drosophila melanogaster 59

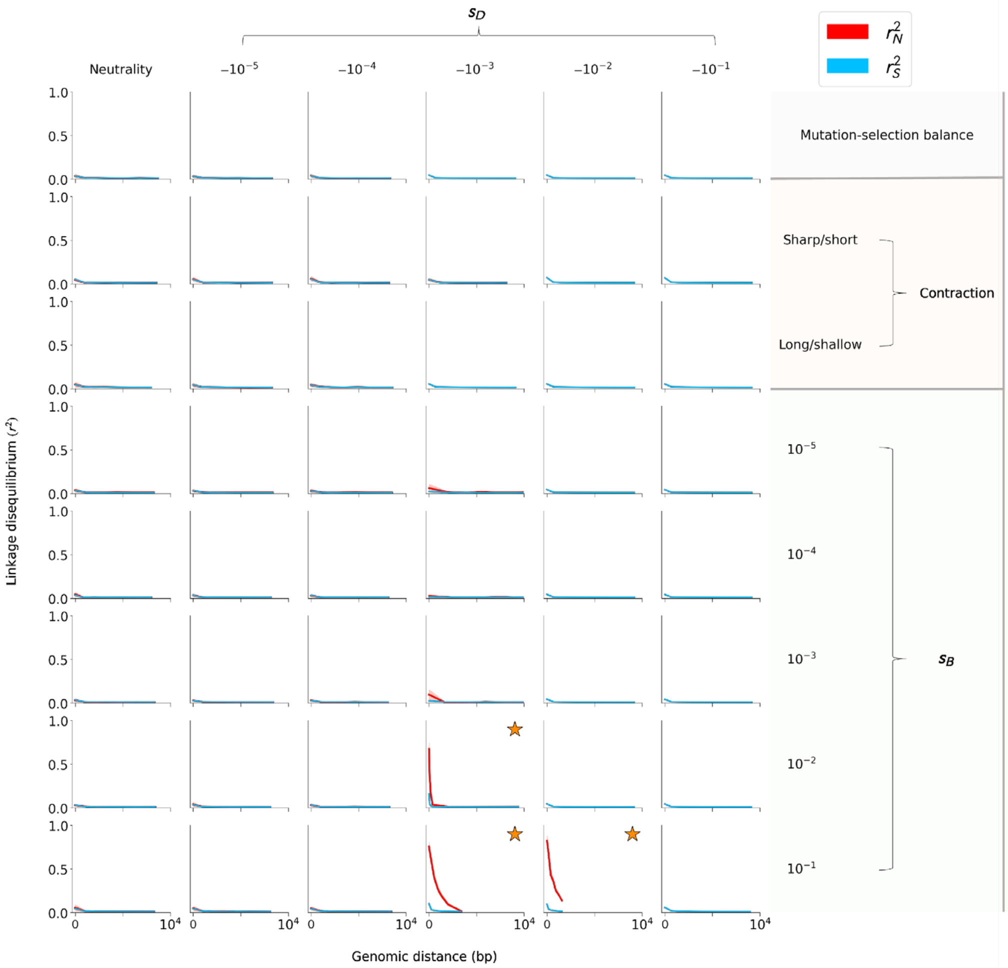

Figure S1: r_N^2 and r_S^2 measured among common variants in a range of simulated evolutionary scenarios, where $\rho = 10^{-5}$. Populations of size $N_e = 10^4$ were simulated with a range of deleterious selection coefficients (s_D), and with $\rho/\mu = 0.1$ (see Figures 2 and 3 for $\rho/\mu = 1$ and $\rho/\mu = 10$). Top row: r_N^2 and r_S^2 measured in populations at mutation-selection balance. When $s_D \geq 10^{-2}$, no intermediate frequency non-synonymous variants were observed, and therefore no r_N^2 curves are plotted in these columns. Second and third rows: r_N^2 and r_S^2 measured in populations experiencing a sharp, short population size contraction (second row) and a long, shallow contraction (third row). Fourth row onwards: r_N^2 and r_S^2 measured in populations experiencing a selective sweep. A total of 250 replicate burn-in populations were simulated until mutation-selection balance had been reached ($10N_e$ generations), followed by five replicates for each burn-in population for the bottleneck and selective sweep scenarios (Section 2). Orange stars denote significantly elevated r_N^2 compared to r_S^2. Under the strongest selective conditions ($s_B = 10^{-1}, s_D = \{-10^{-3}, -10^{-2}\}$), nearly all common deleterious variants were physically close to the adaptive variant, resulting in apparently truncated LD curves. 99% confidence intervals are plotted as a shaded region around each non-synonymous LD curve, though this region is narrow around some curves.

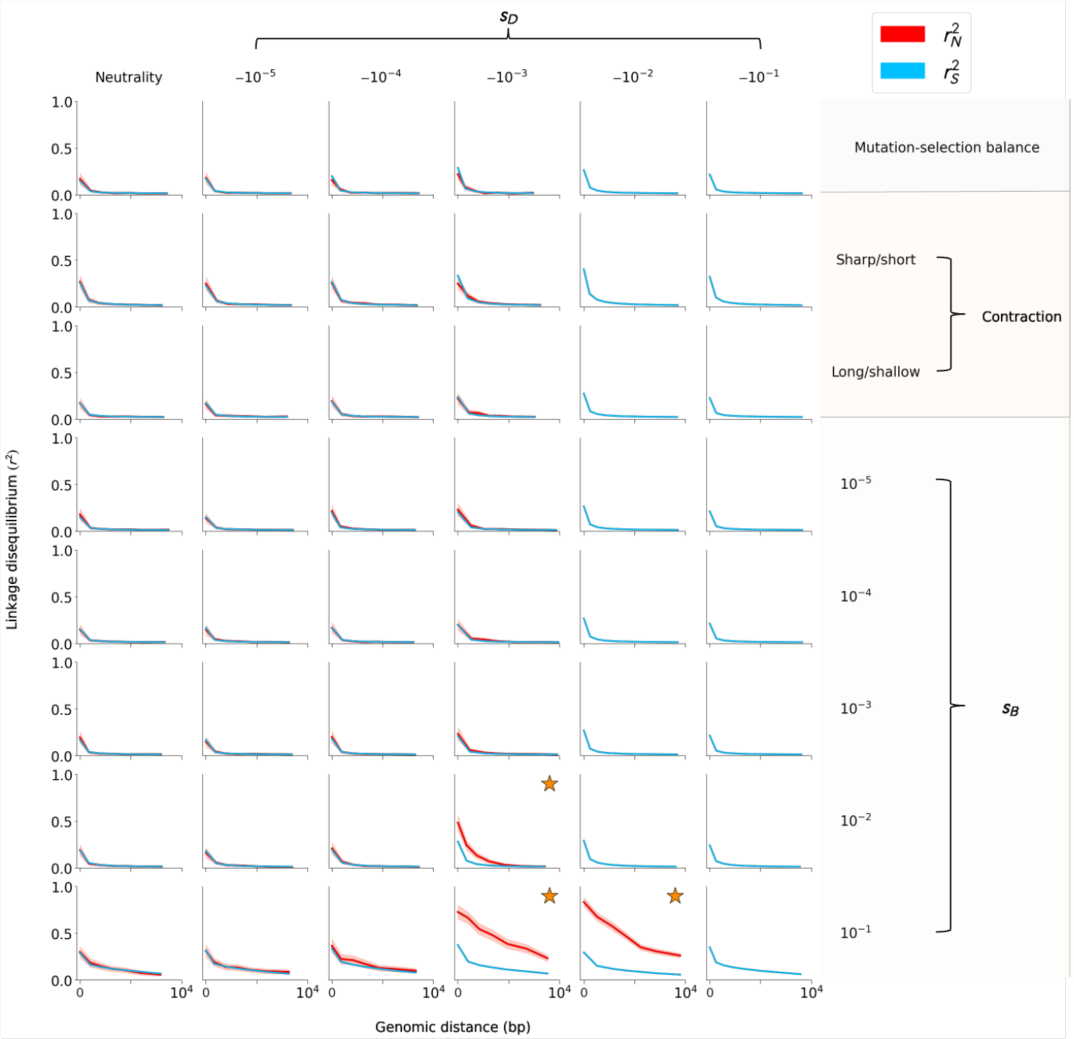

Figure S2: r_N^2 and r_S^2 measured among common variants in a range of simulated evolutionary scenarios, where $\rho = 10^{-6}$. Analogous to Figure 1, but for simulations performed with $\rho = 10^{-6}$ (i.e. $\rho/\mu = 1$). As expected, LD decays more rapidly in this regime than with $\rho/\mu = 10$. As with $\rho/\mu = 10$, AUC($r_N^2 - r_S^2$) was significantly greater than zero when $N_e s_D > 1$ and $s_B > s_D$.

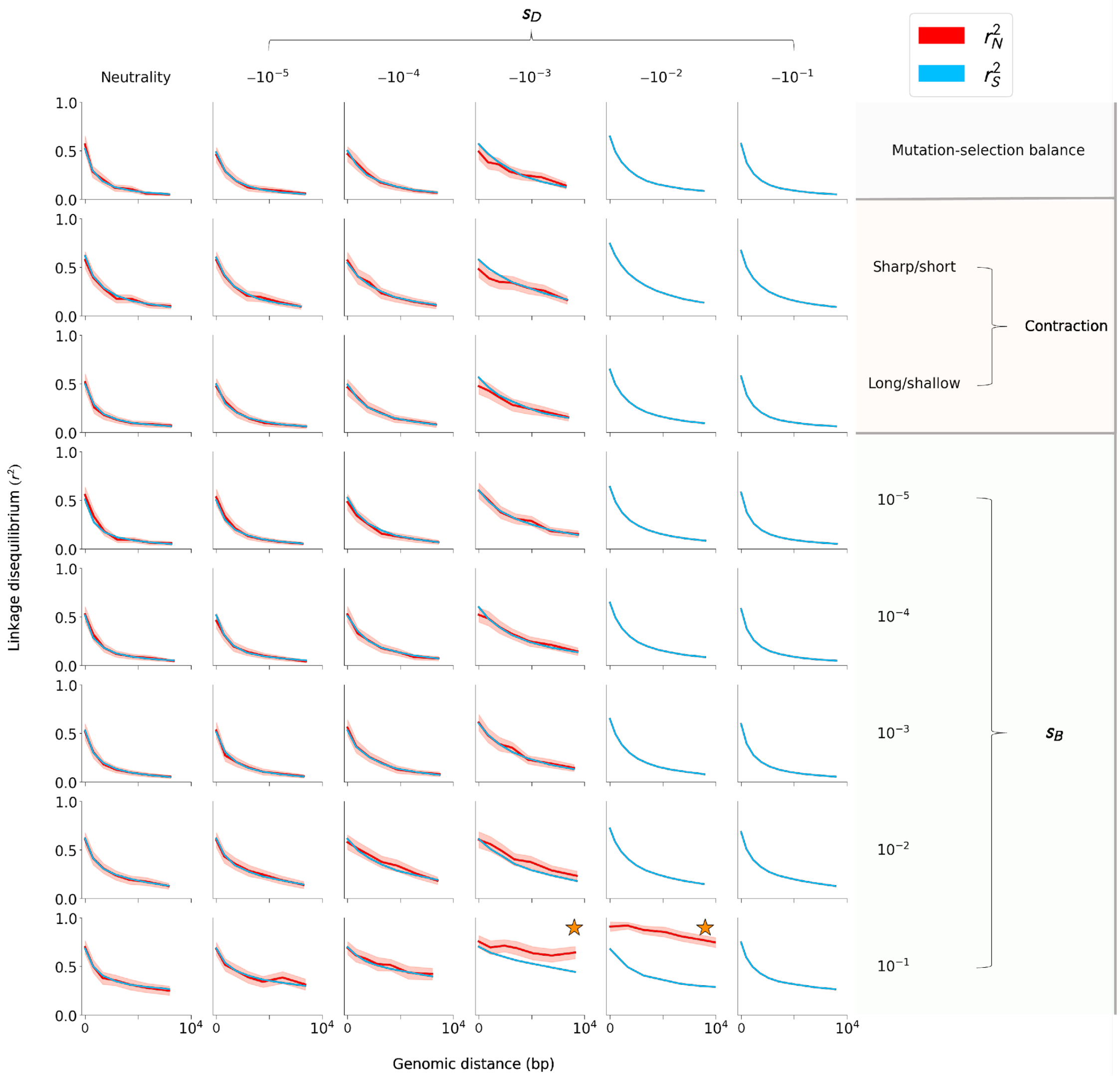

Figure S3: r_N^2 and r_S^2 measured among common variants in a range of simulated evolutionary scenarios, where $\rho = 10^{-7}$. Analogous to Figure 1, but for simulations performed with $\rho = 10^{-7}$ (i.e. $\rho/\mu = 10^{-1}$). As expected, LD decays much more slowly in this regime than with $\rho/\mu = 1$. Moreover, AUC($r_N^2 - r_S^2$) was significantly greater than zero only when $s_B = 10^{-1}$. While AUC($r_N^2 - r_S^2$) > 0 when $s_B = 10^{-2}$ and $s_D = -10^{-3}$, the difference was not significant.

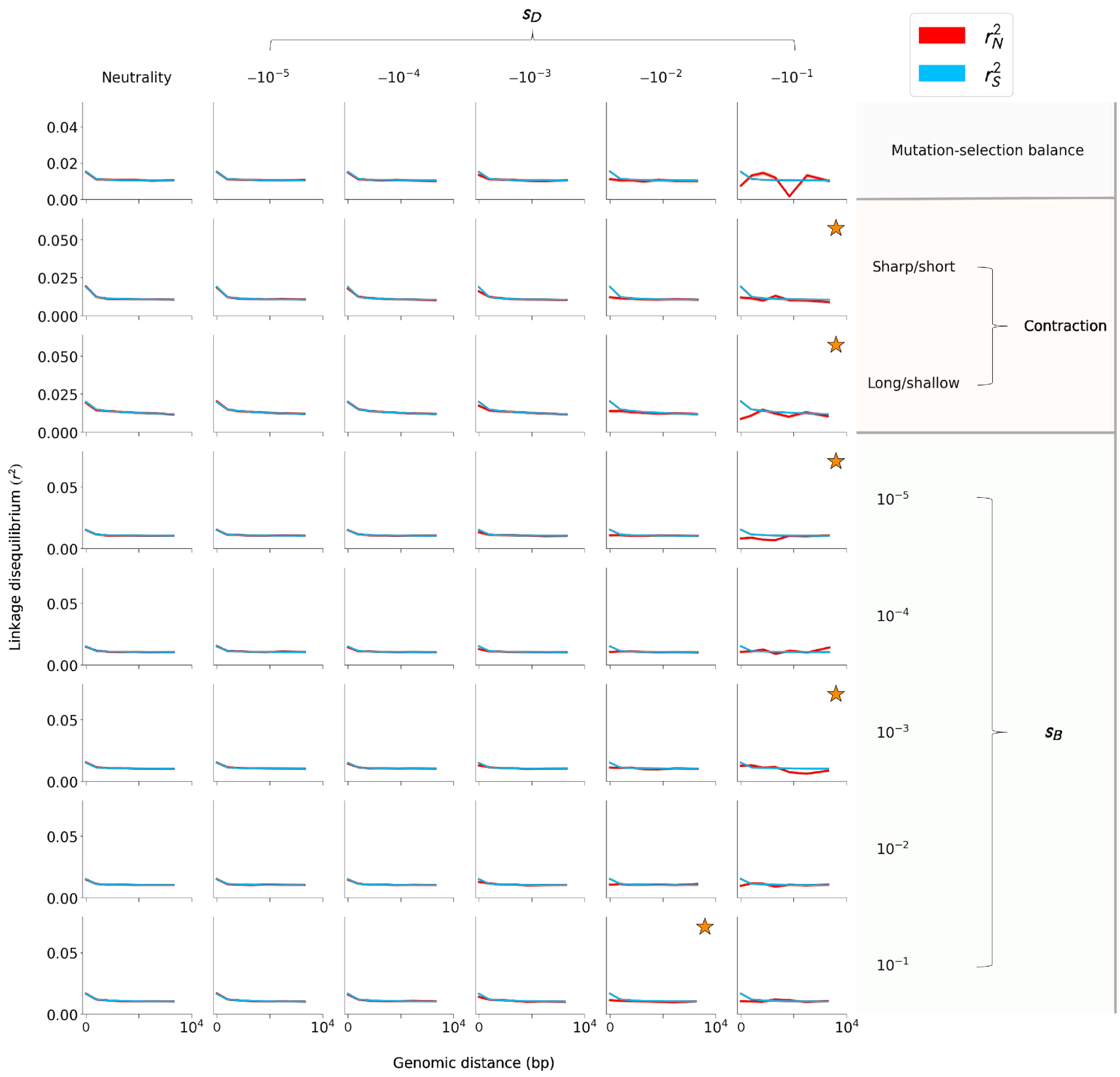

Figure S4: r_N^2 and r_S^2 measured among rare variants in a range of simulated evolutionary scenarios, where $\rho = 10^{-5}$. LD patterns among rare variants for the evolutionary scenarios discussed in the Main Text, with $\rho = 10^{-5}$ (i.e. $\rho/\mu = 10$). LD among both synonymous and non-synonymous variants decays extremely rapidly, and differences between r_N^2 and r_S^2 are typically only apparent over very short length scales.

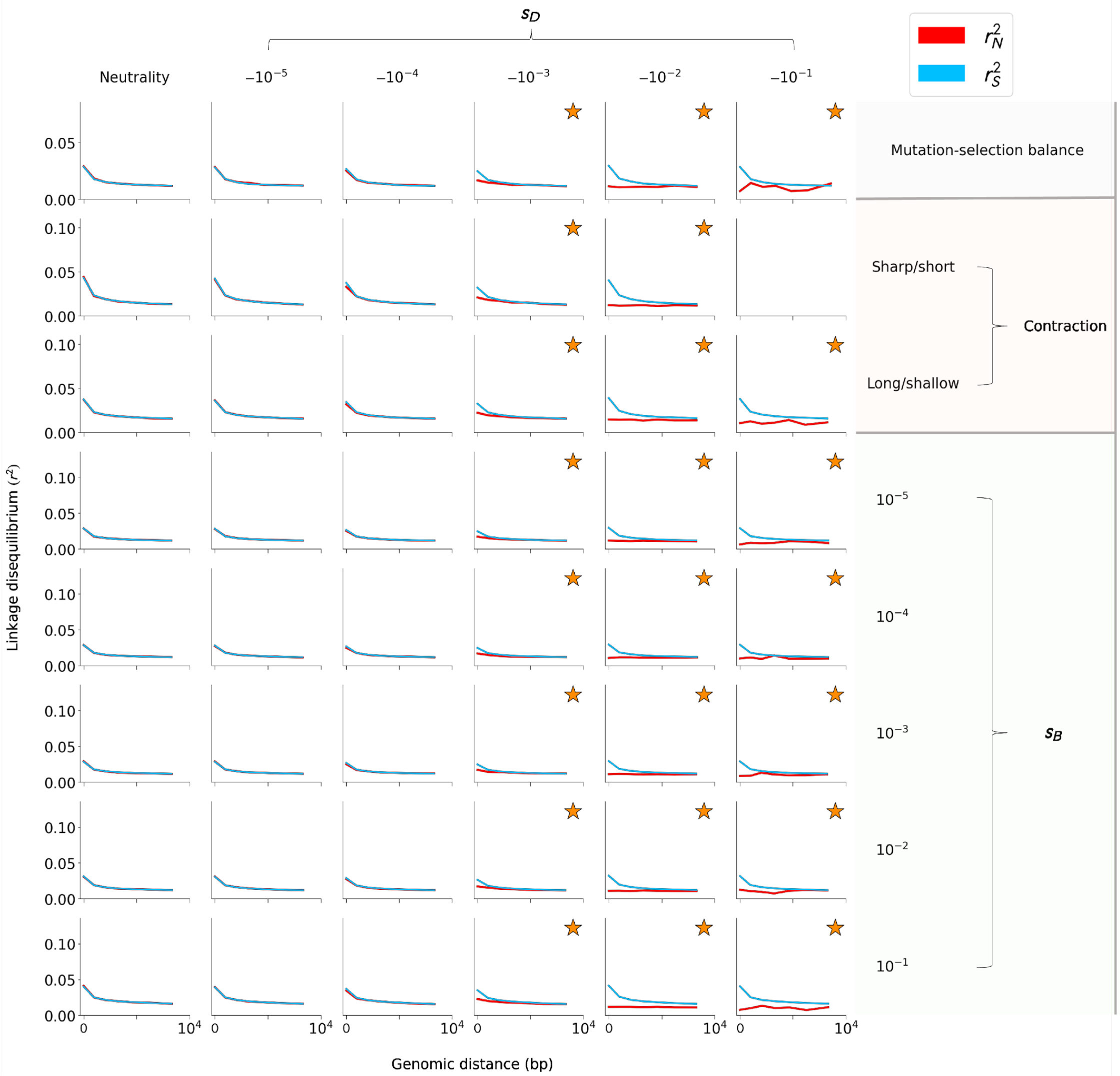

Figure S5: r_N^2 and r_S^2 measured among rare variants in a range of simulated evolutionary scenarios, where $\rho = 10^{-6}$. LD patterns among rare variants for the simulations discussed in the Main Text. AUC($r_N^2 - r_S^2$) is significantly less than zero in all simulations where $s_D > 1/N_e$ except for the short, sharp contraction with $s_D = -10^{-1}$, in which there were an insufficient number of both rare synonymous and non-synonymous variants to create LD curves.

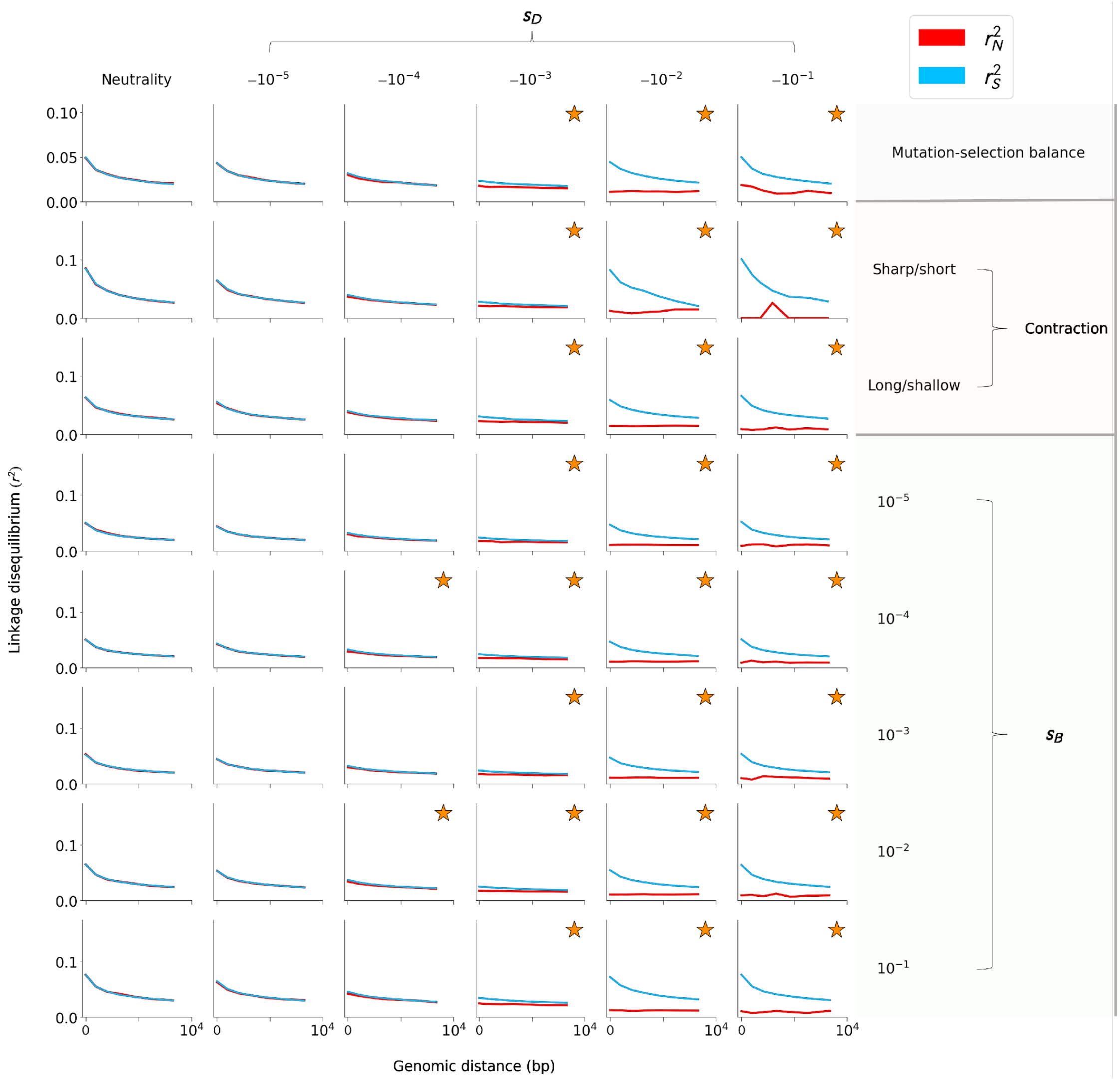

Figure S6: r_N^2 and r_S^2 measured among rare variants in a range of simulated evolutionary scenarios, where $\rho = 10^{-7}$. LD patterns among rare variants for the evolutionary scenarios discussed in the Main Text, with $\rho = 10^{-7}$ (i.e. $\rho/\mu = 10^{-1}$). AUC($r_N^2 - r_S^2$) is significantly less than zero in all simulations where $s_D > 1/N_e$, and in two instances also when $s_D = 1/N_e$.

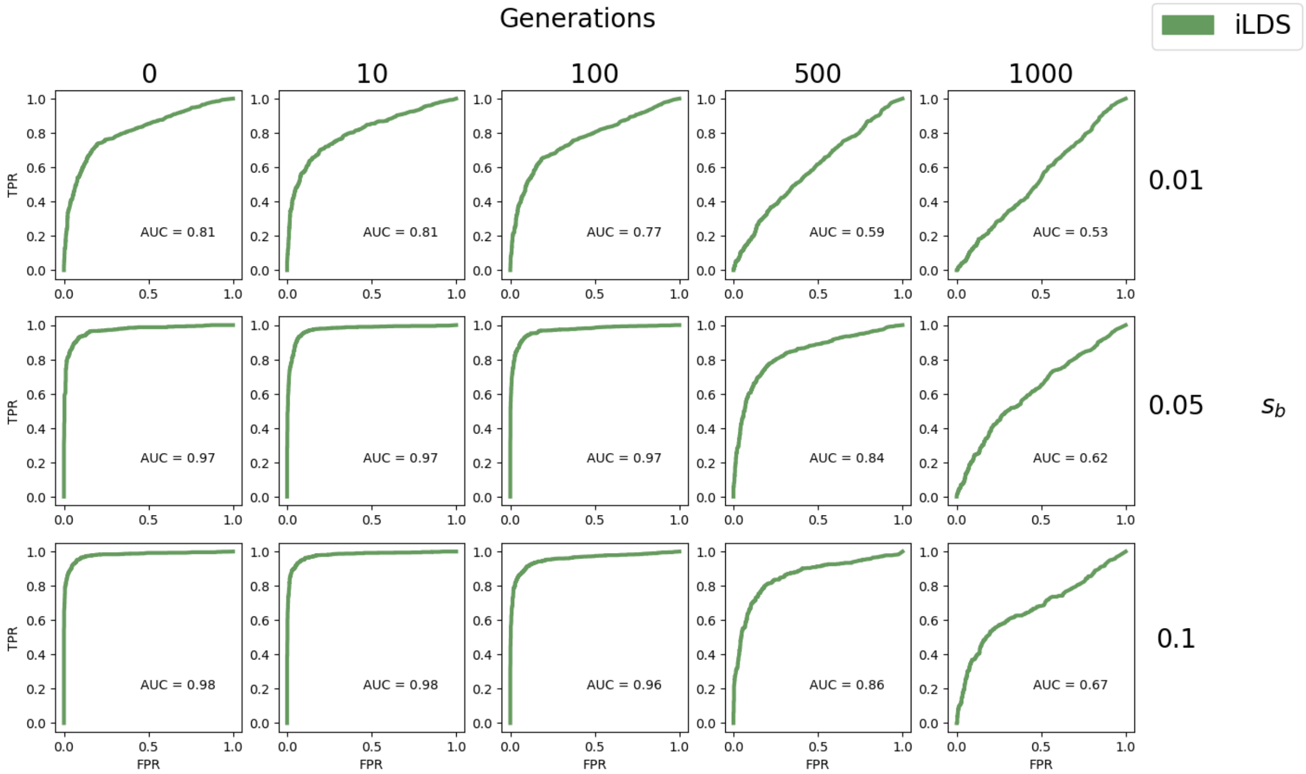

Figure S7: Power analysis for iLDS under various sweep scenarios versus a constant N_e population at mutation-selection balance. Receiver operating characteristic (ROC) curves showing true (TPR) and false positive rates (FPR) of iLDS over a range of significance values for sweeps of various strengths (rows) and number of generations since the sweep ended (columns). All populations experienced constant deleterious selection strength of $s_D = -10^{-3}$.

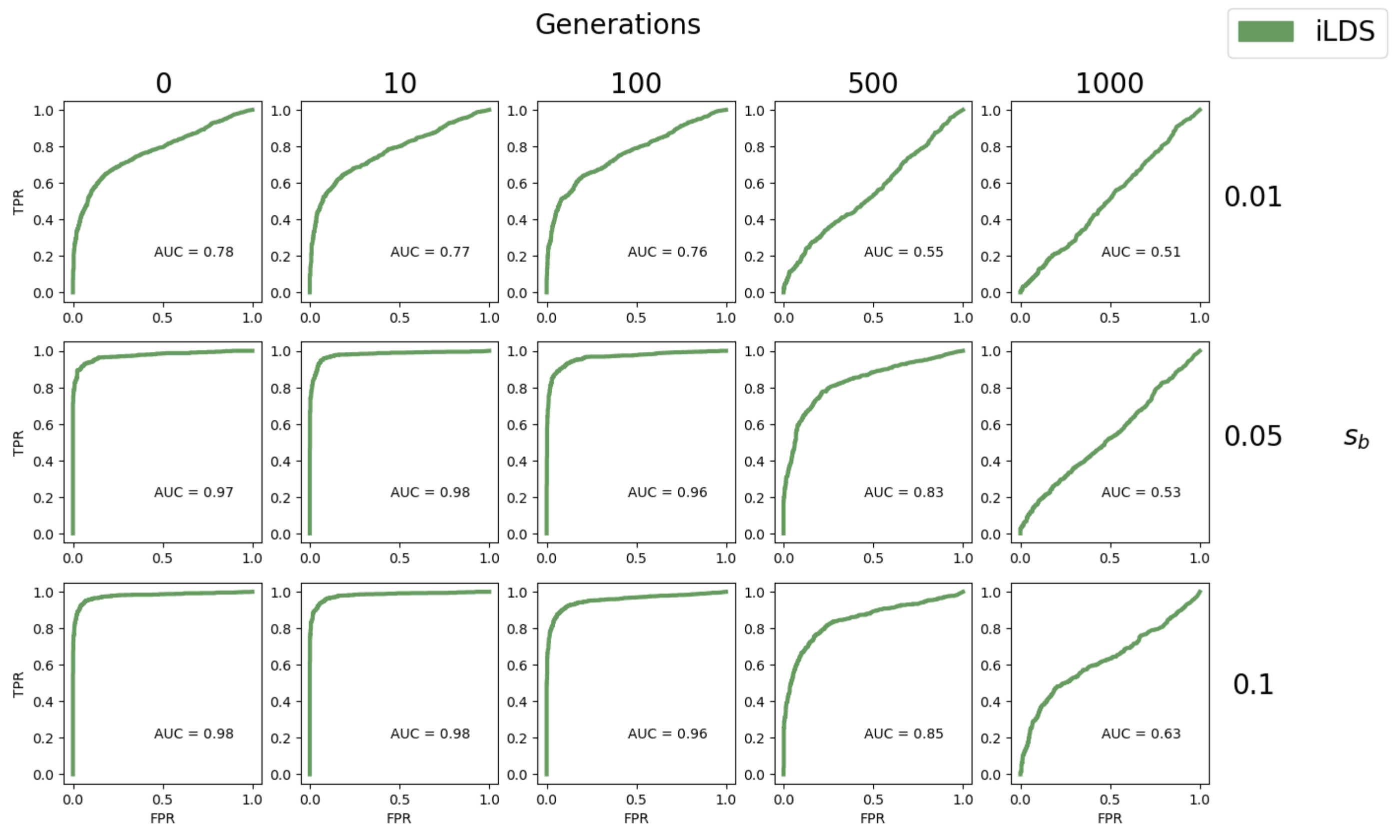

Figure S8: Power analysis for iLDS under various sweep scenarios versus populations experiencing a sharp, short bottleneck under mutation-selection balance. Receiver operating characteristic (ROC) curves showing true and false positive rates of iLDS over a range of significance values for sweeps of various strengths (rows) and number of generations since the sweep ended (columns). Here, the false positive rate is assessed by the rate of mis-classifying populations experiencing a short, sharp bottleneck but no positive selection (see Section 4.4). All populations experienced constant deleterious selection strength of $s_D = -10^{-3}$.

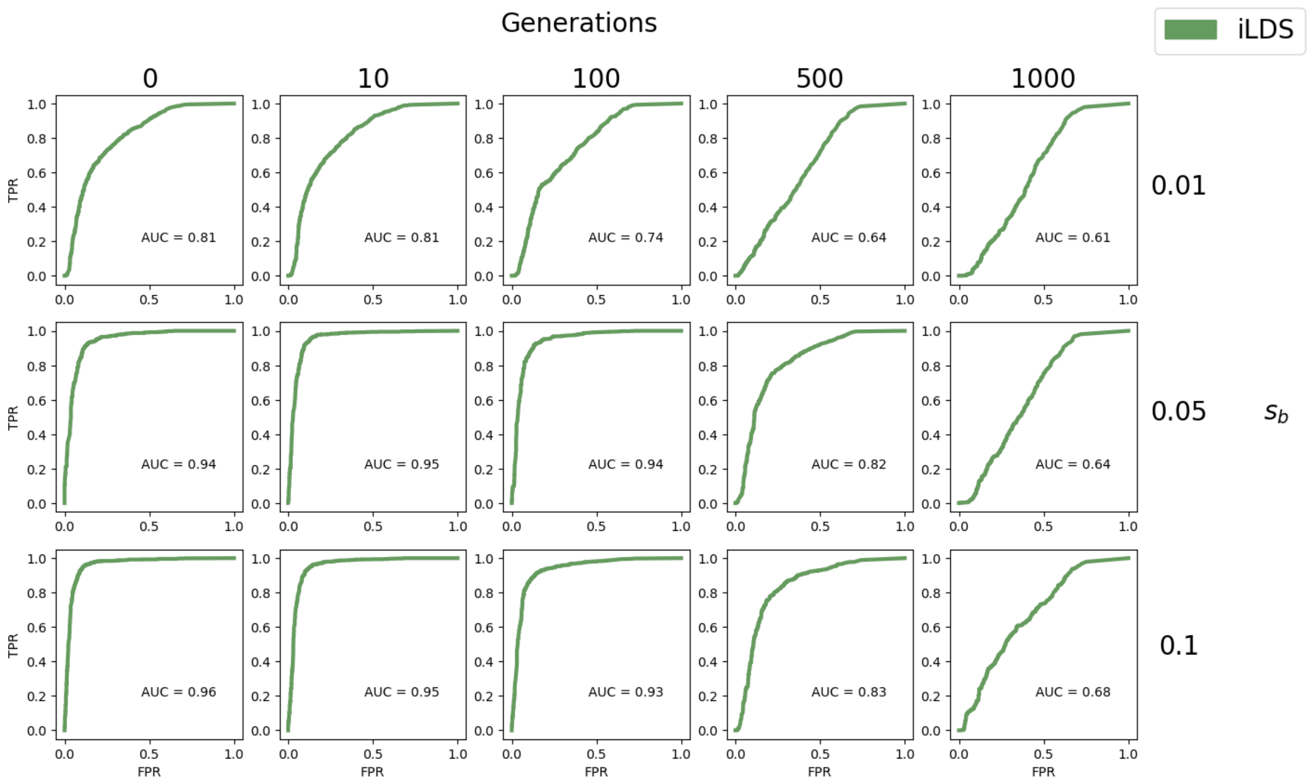

Figure S9: Power analysis for iLDS under various sweep scenarios versus populations experiencing a long, shallow bottleneck. Receiver operating characteristic (ROC) curves showing true and false positive rates of iLDS over a range of significance values for sweeps of various strengths (rows) and number of generations since the sweep ended (columns). Here, the false positive rate is assessed by the rate of mis-classifying populations experiencing a long, shallow bottleneck but no positive selection (see Section 4.4). All populations experienced constant deleterious selection strength of $s_D = -10^{-3}$.

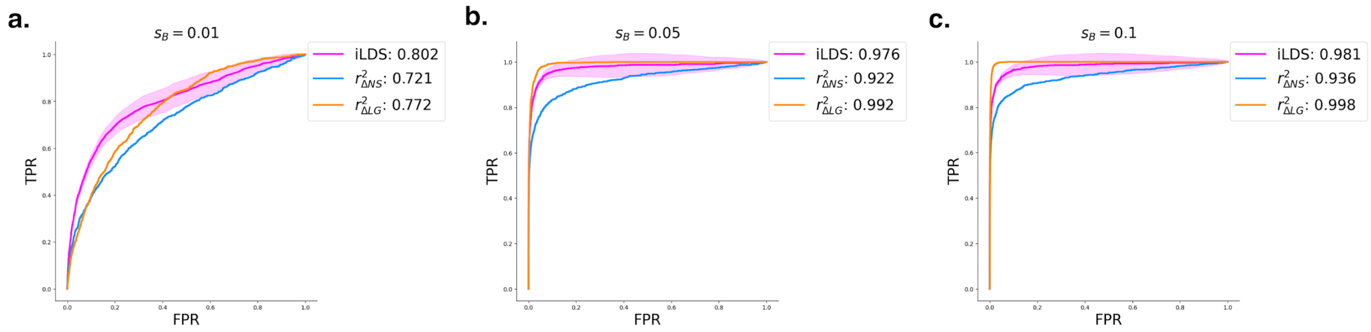

Figure S10: Power analysis for $r^2_{\Delta NS}$, $r^2_{\Delta LG}$ and iLDS. Receiver operating characteristic (ROC) curves showing true and false positive rates of $r^2_{\Delta NS}$, $r^2_{\Delta LG}$, and iLDS over a range of significance values for sweeps of various strengths (Supplementary Section 4.4). All sweeps were sampled at the time when selection ceased and the adaptive allele reached 50% frequency. All populations experienced constant deleterious selection strength of $s_D = -10^{-3}$. The AUROC for each statistic is shown in the legend.

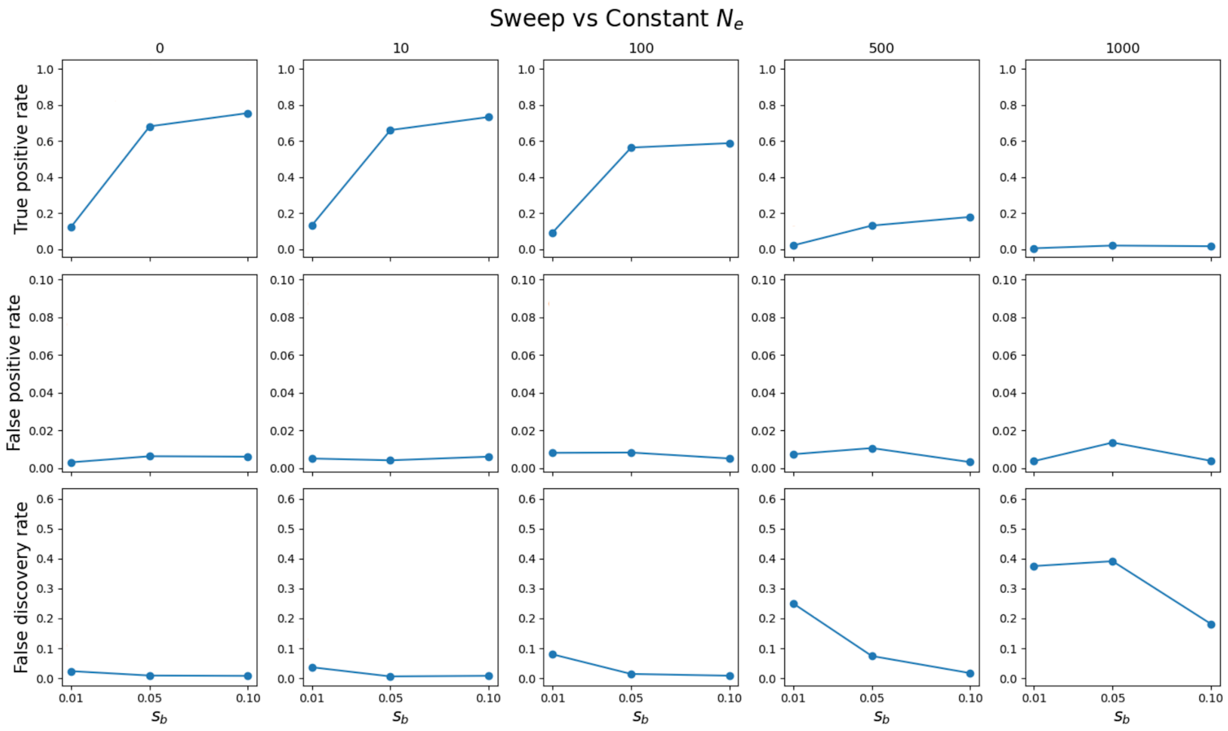

Figure S11: True positive, false positive, and false discovery rates for sweeps against constant N_e. True positive, false positive, and false discovery rates of iLDS at $\alpha = 0.05$ threshold when detecting selective sweeps against the genomic background of a population of constant size at mutation-selection balance. The selection strength of sweeps (rows) and number of generations since selection ceased (columns) were varied.

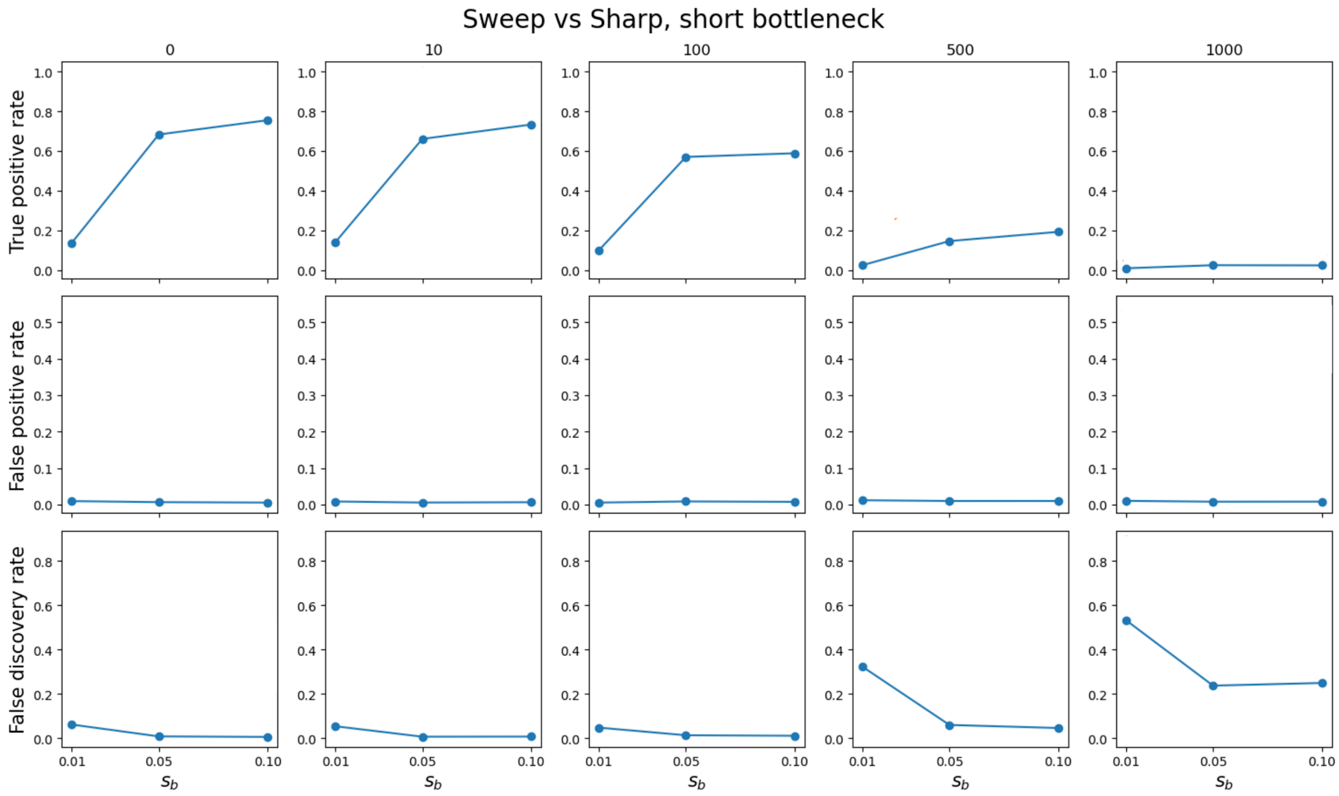

Figure S12: True positive, false positive, and false discovery rates for sweeps against a sharp, short bottleneck. True positive, false positive, and false discovery rates of iLDS at $\alpha = 0.05$ threshold when detecting selective sweeps against the genomic background of a population experiencing a sharp, short bottleneck. The selection strength of sweeps (rows) and number of generations since selection ceased (columns) were varied.

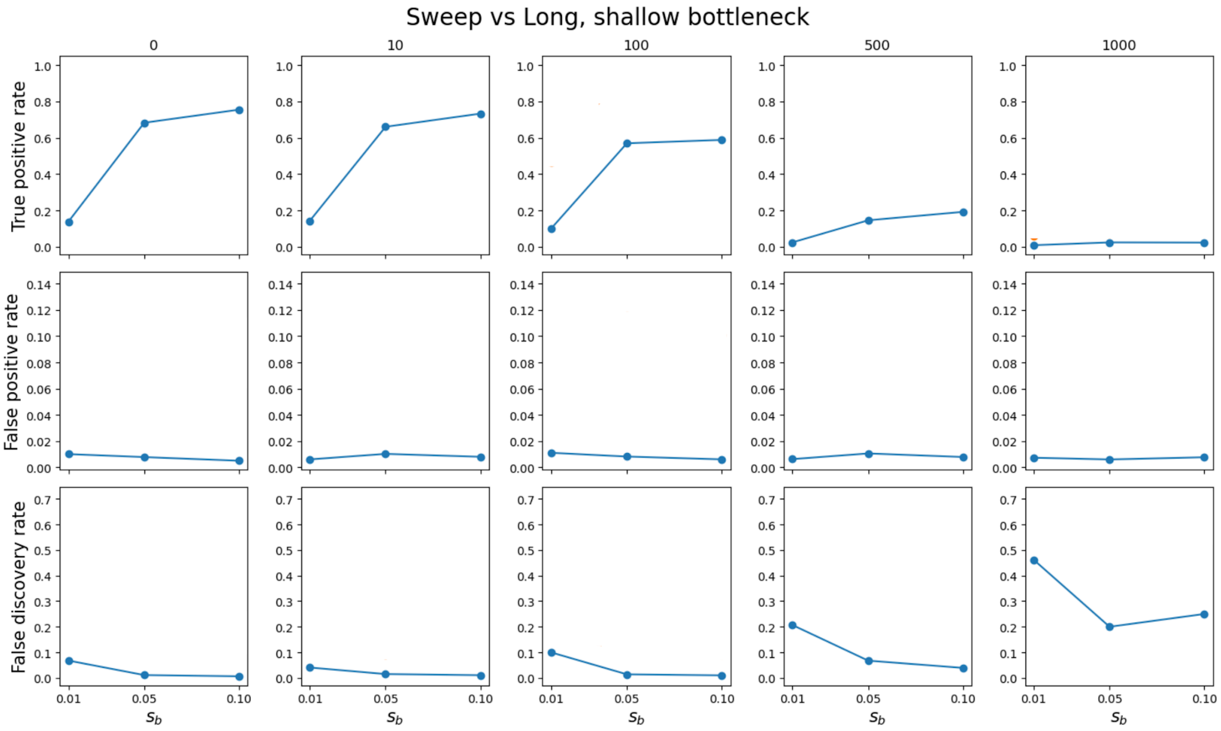

Figure S13: True positive, false positive, and false discovery rates for sweeps against a long, shallow bottleneck. True positive, false positive, and false discovery rates of iLDS at $\alpha = 0.05$ threshold when detecting selective sweeps against the genomic background of a population experiencing a long, shallow bottleneck. The selection strength of sweeps (rows) and number of generations since selection ceased (columns) were varied.

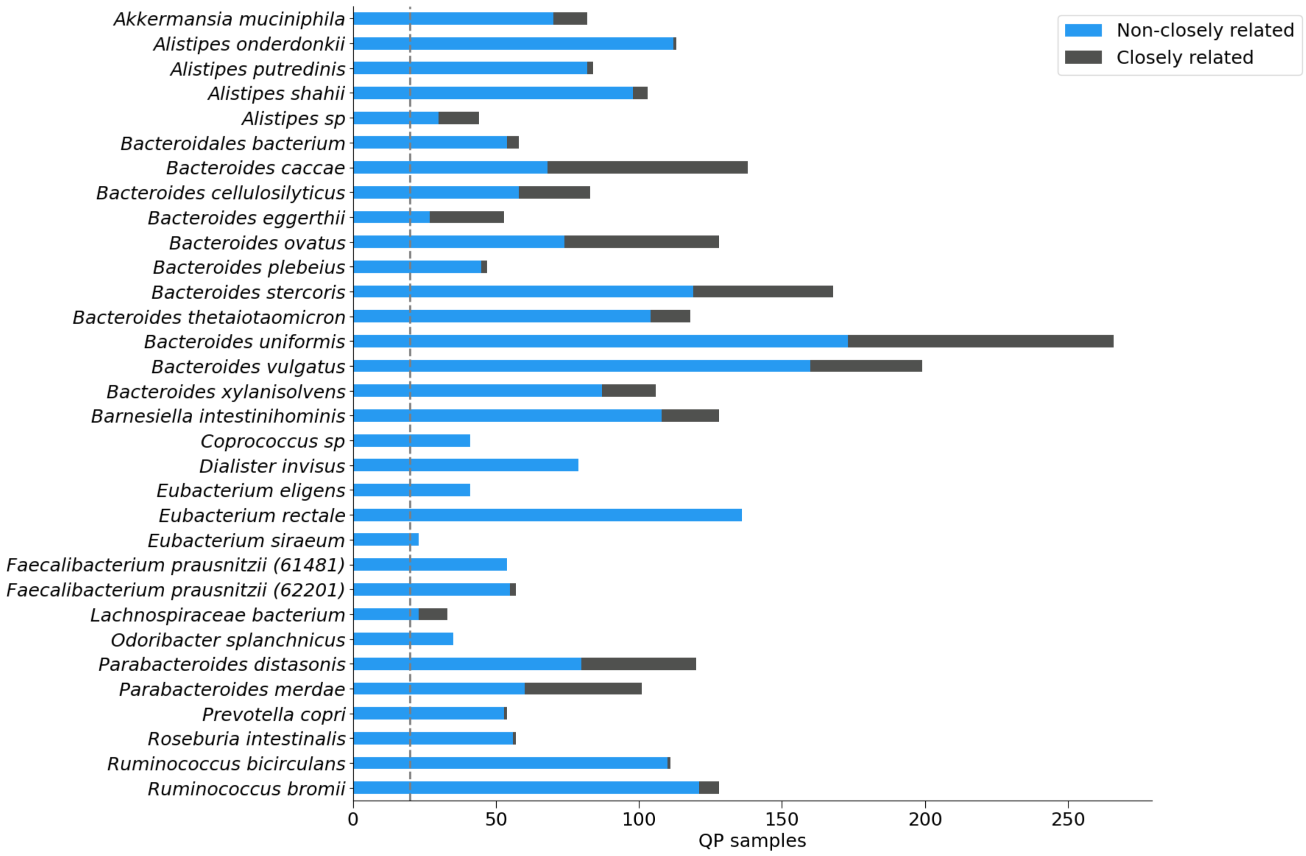

Figure S14: Number of quasi-phased samples by species. The number of quasi-phased samples (Supplementary Section 3.1.5) belonging to the largest clade for each of the 32 species analyzed. Samples are divided between closely related ($d < 5 \times 10^{-4}$ at 4D sites) and non-closely related samples.

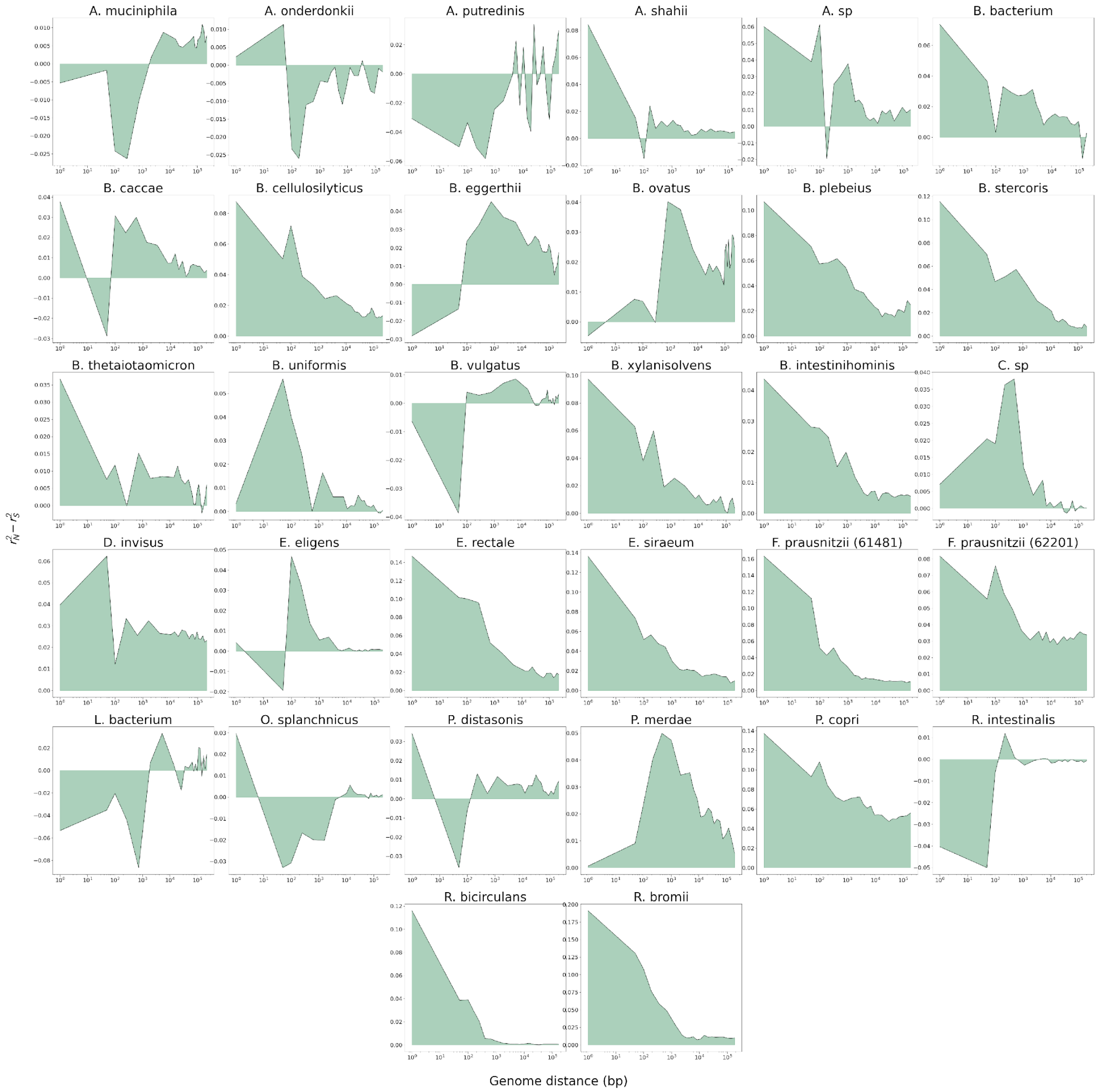

Figure S15: ($r_N^2 - r_S^2$) measured in prevalent commensal gut microbiota for common variants. Here, the function ($r_N^2 - r_S^2$) is plotted (black line), and AUC($r_N^2 - r_S^2$) is shown with the green shaded region. In a majority of species, ($r_N^2 - r_S^2$) tends to decrease as a function of distance, or reach a maximum at some intermediate distance. A minority of species (such as *Akkermansia muciniphilia*, top left) show an increase of ($r_N^2 - r_S^2$) with distance.

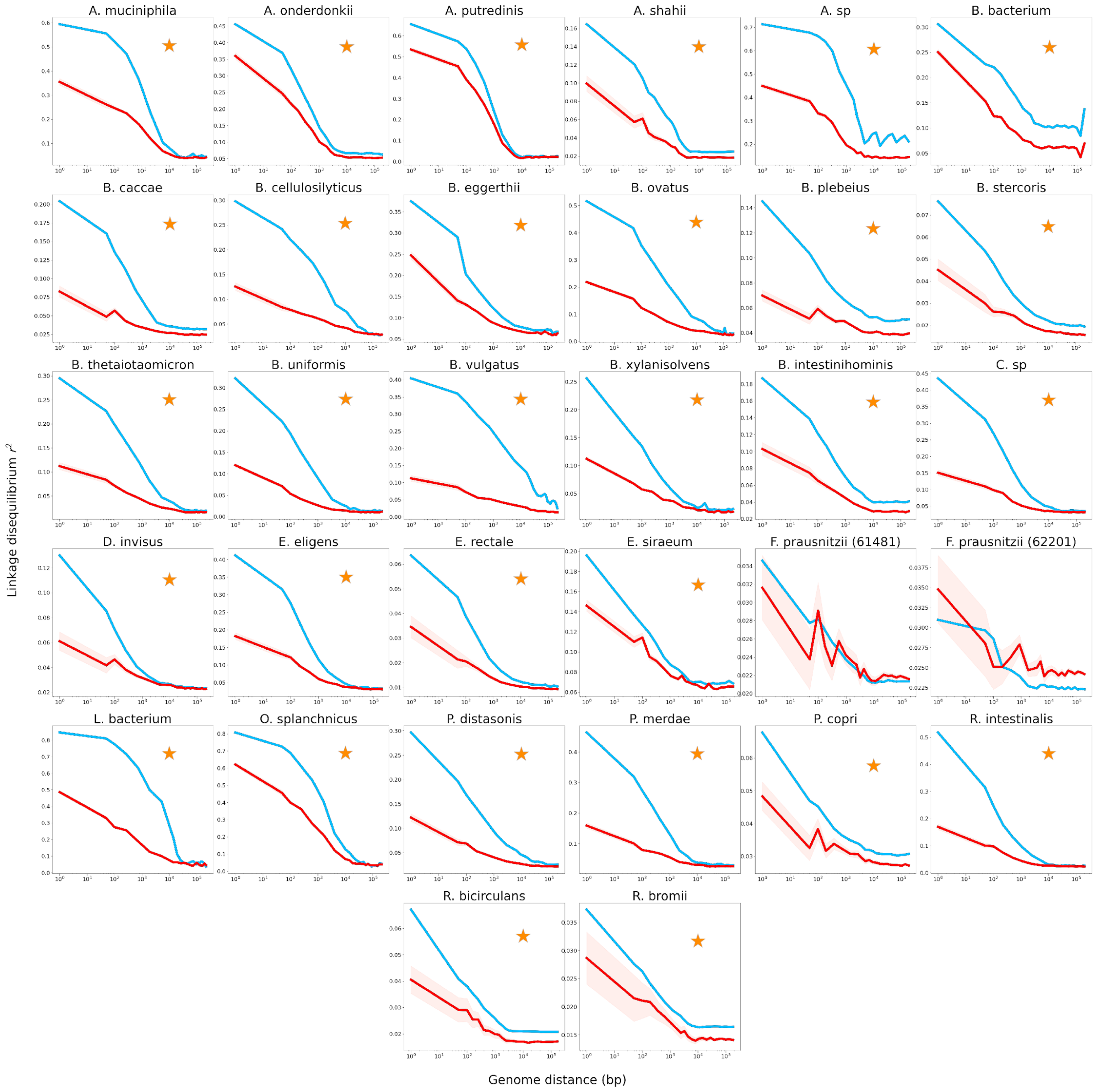

Figure S16: r_N^2 and r_S^2 measured in prevalent commensal gut microbiota for rare (MAF ≤ *0.05*) variants. r_N^2 is shown in red, while r_S^2 is shown in blue. Contrary to patterns seen among common variants, r_S^2 is significantly elevated above r_N^2 among rare variants in all but one species. This depression of LD between rare non-synonymous variants has been observed previously, and has been shown to arise both due to Hill-Robertson interference between deleterious variants[9–11] and as a result of epistatic interactions between deleterious variants[12–15].

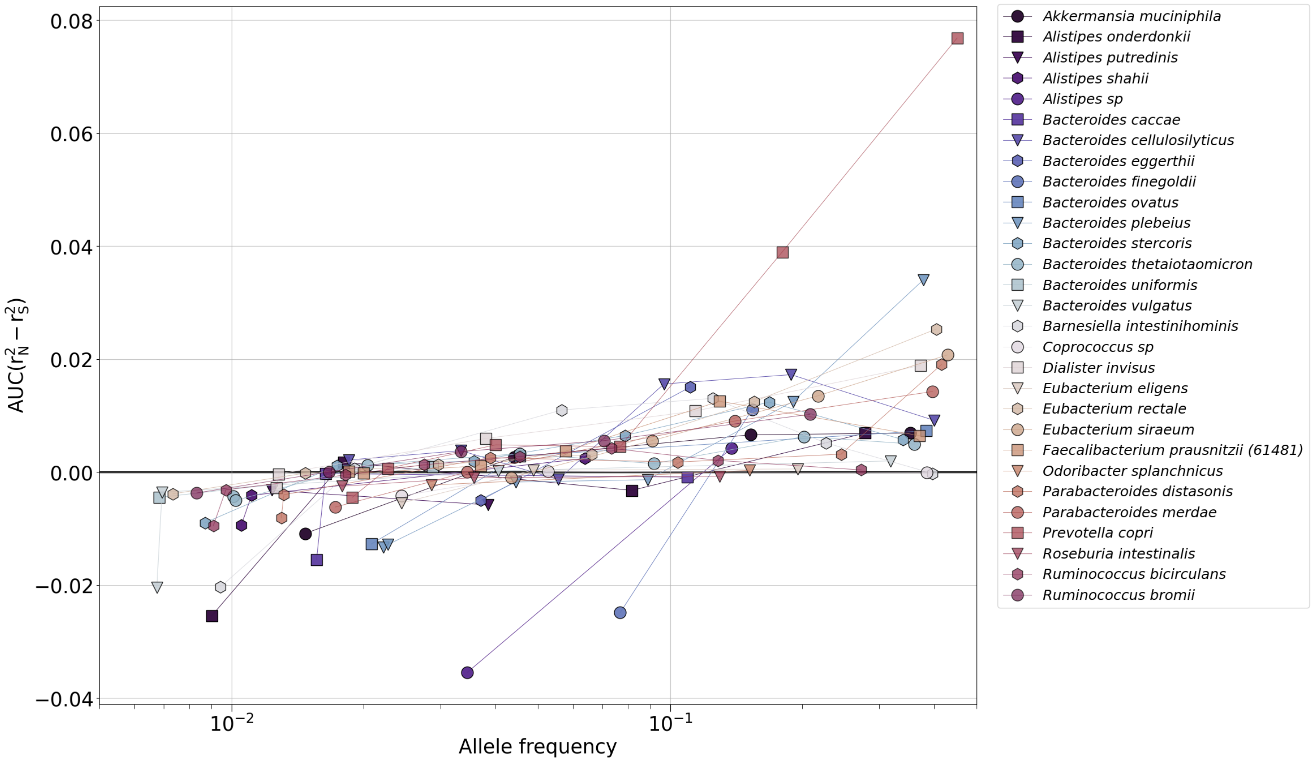

Figure S17: AUC($r_N^2 - r_S^2$) measured in prevalent commensal gut microbiota for varying allele frequencies. For each species, all variants were binned by allele frequency interval, and AUC($r_N^2 - r_S^2$) calculated for each bin. AUC($r_N^2 - r_S^2$) is universally an increasing function of allele frequency, with a transition between negative AUC($r_N^2 - r_S^2$) and positive AUC($r_N^2 - r_S^2$) typically occurring around $f \approx 0.05$.

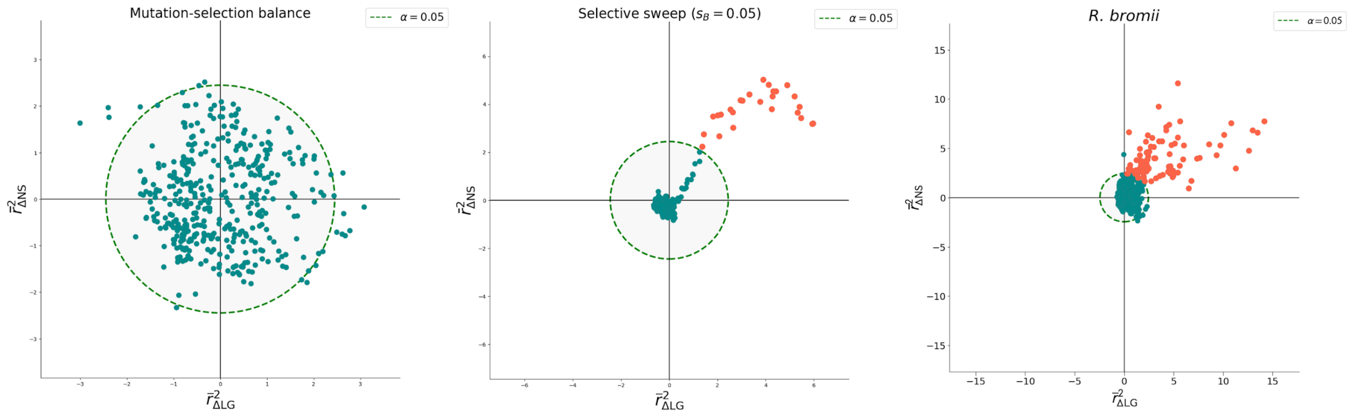

Figure S18: $\bar{r}^2_{\Delta NS}$ versus $\bar{r}^2_{\Delta LG}$ in simulations and data. Here, we plot the two components of iLDS ($\bar{r}^2_{\Delta NS}$ and $\bar{r}^2_{\Delta LG}$, the normalized differences between non-synonymous versus synonymous LD and local versus genome-wide LD) for simulations and data. Significant windows are colored orange, and non-significant windows green. Because the formula for iLDS is $\left(\bar{r}^2_{\Delta(NS)}\right)^2 + \left(\bar{r}^2_{\Delta(LG)}\right)^2$, the value of the statistic can be thought of as the square of the distance from the origin of a point in $(\bar{r}^2_{\Delta NS}, \bar{r}^2_{\Delta LG})$ space. The green dashed line is a circle with a radius corresponding to the square root of the 95th percentile of a χ^2 distribution with two degrees of freedom—an iLDS value exceeding this threshold is necessary but not sufficient for a window to be called significant. In a simulation at mutation-selection balance (left) the iLDS statistic may surpass the critical threshold, but the additional checks of significance (non-synonymous LD exceeding synonymous LD, local LD exceeding genome-wide LD) prevent false inferences of selection. By contrast, in a simulated selective sweep (center) and real data (right), there is a notable positive correlation between $\bar{r}^2_{\Delta LG}$ and $\bar{r}^2_{\Delta NS}$ in a tail of windows which also tend to be called as significant.

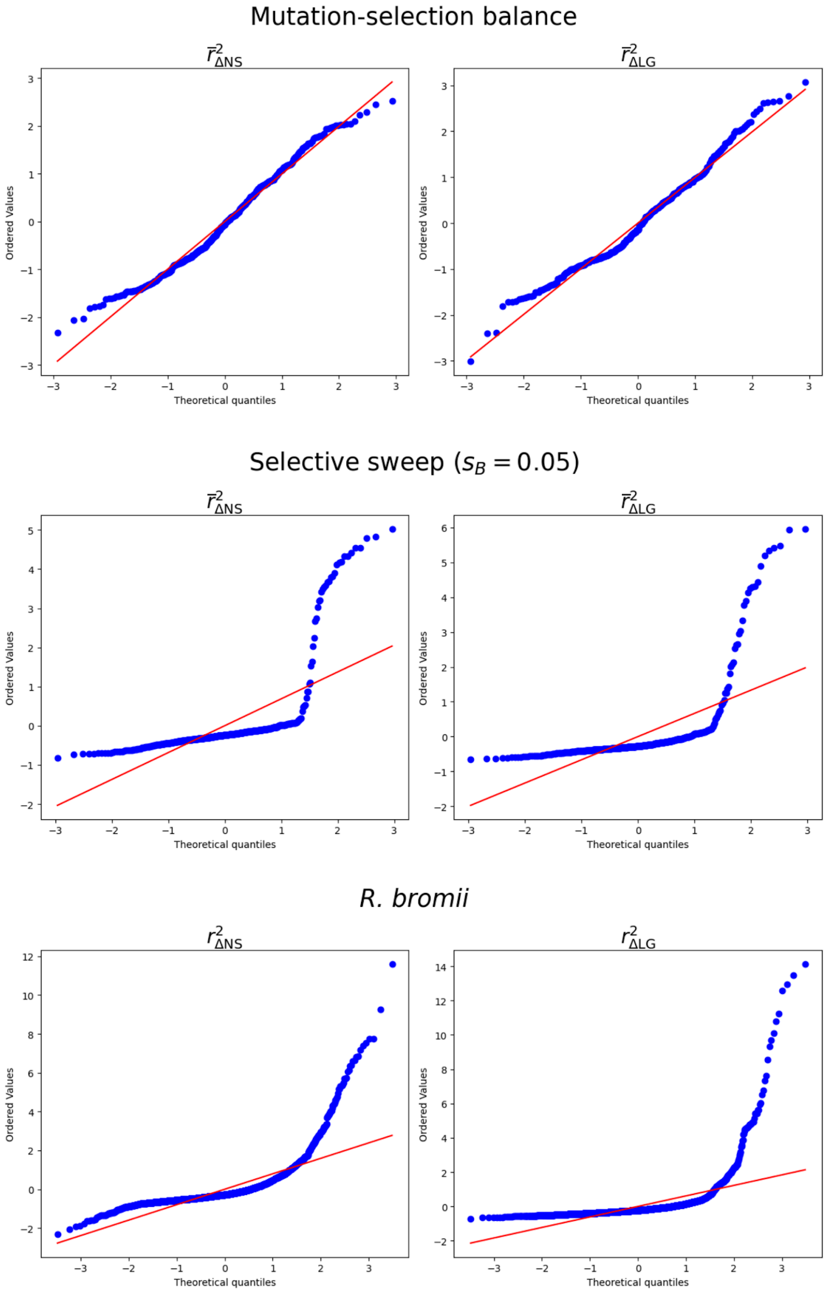

Figure S19: QQ plots of $\bar{r}^2_{\Delta NS}$ versus $\bar{r}^2_{\Delta LG}$ in simulations and data. Quantile-quantile plots showing the quantiles of $\bar{r}^2_{\Delta NS}$ and $\bar{r}^2_{\Delta LG}$ versus the expected quantiles if each statistic had a standard normal distribution for a simulation at mutation-selection balance (top), a simulated selective sweep (middle), and real data for *R. bromii* (bottom). While each statistic roughly follows a standard normal distribution at mutation-selection balance, the simulated selective sweeps and real data exhibit substantial departures from the mutation-selection balance scenario, particularly driven by a long right tail of large $\bar{r}^2_{\Delta NS}$ and $\bar{r}^2_{\Delta LG}$ values.

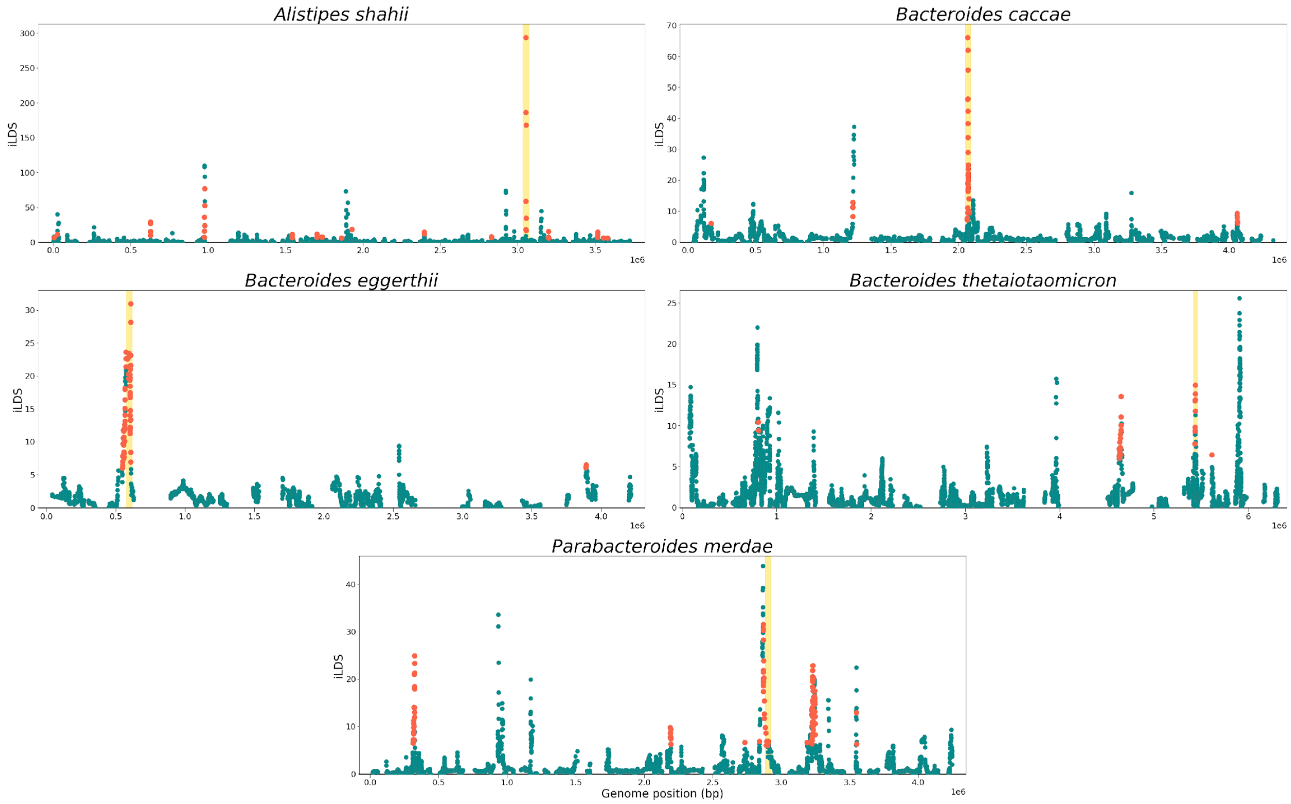

Figure S20: *susC*/*susD* genes under selection. *susC*/*susD* genes were detected as being under selection in five unique species. Here, the iLDS scans for these species are plotted, and the location of the *susC*/*susD* locus under selection in that species is shown with a gold bar.

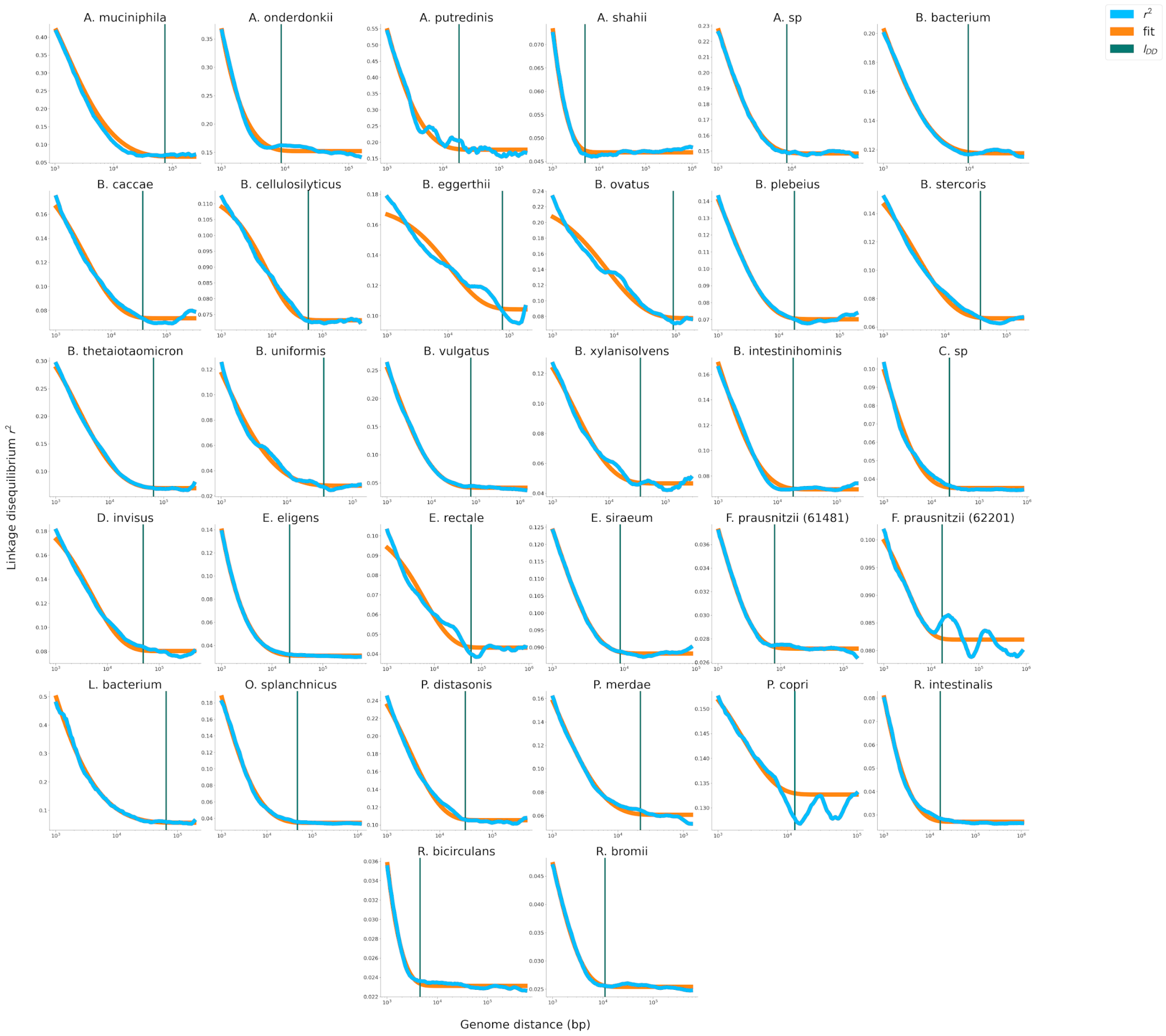

Figure S21: Estimates of l_{DD} for common commensal gut microbiota. Using the procedure outlined in Supplementary Section 4.1, we fit Equation S7 to r_S^2, and used the parameters of this fit to infer the decay distance l_{DD} (vertical lines). In general, r_S^2 appears to saturate at some long distance constant value in most species, and l_{DD} appears to approximate the length scale over which this saturation occurs.

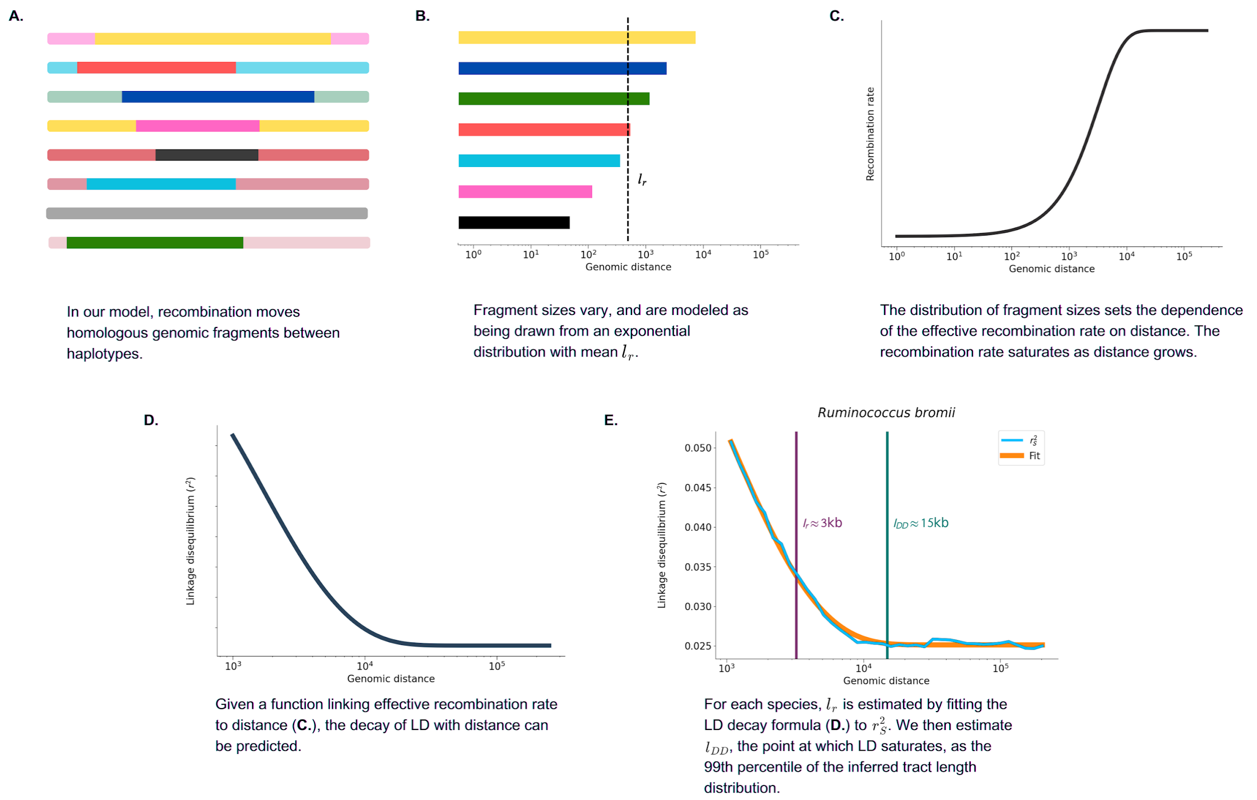

Figure S22: Schematic of method for inferring l_r and l_{DD}. Visual illustration of the procedure outlined in Supplementary Section 4.1, where Equation S7 is fit to r_S^2, and the parameters of this fit to infer the decay distance l_{DD} (in turn used to determine the window size for iLDS) in the microbial species examined here.

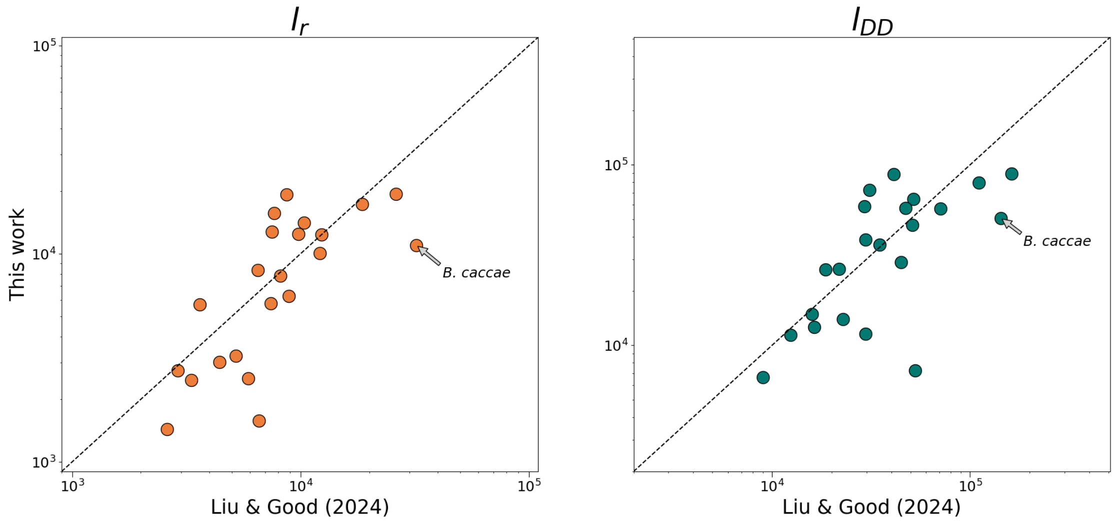

Figure S23: Comparison of l_r and l_{DD} estimates with Liu and Good (2024). We compared the values of l_r (left) and l_{DD} (right) obtained using the procedure outlined in Supplementary Section 4.1 with estimates of these values inferred from Supplementary data in Liu and Good (2024)[16]. The dashed line denotes the 1:1 line.

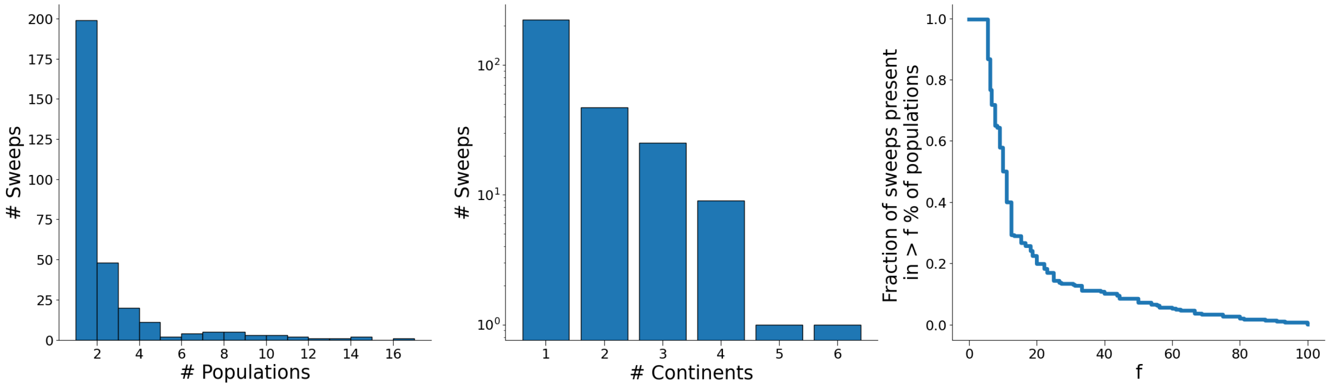

Figure S24: Spread of sweeps across populations. Distributions of the number of populations (left) and continents (center) sweeps have spread to. At right, the fraction of sweeps which have spread to at least a given percentage of the total number of populations in which each species was present. In each plot, it is clear that while it is most common for sweeps to be detected in a small number of populations, there are a substantial number of sweeps which have spread between populations and across continents.

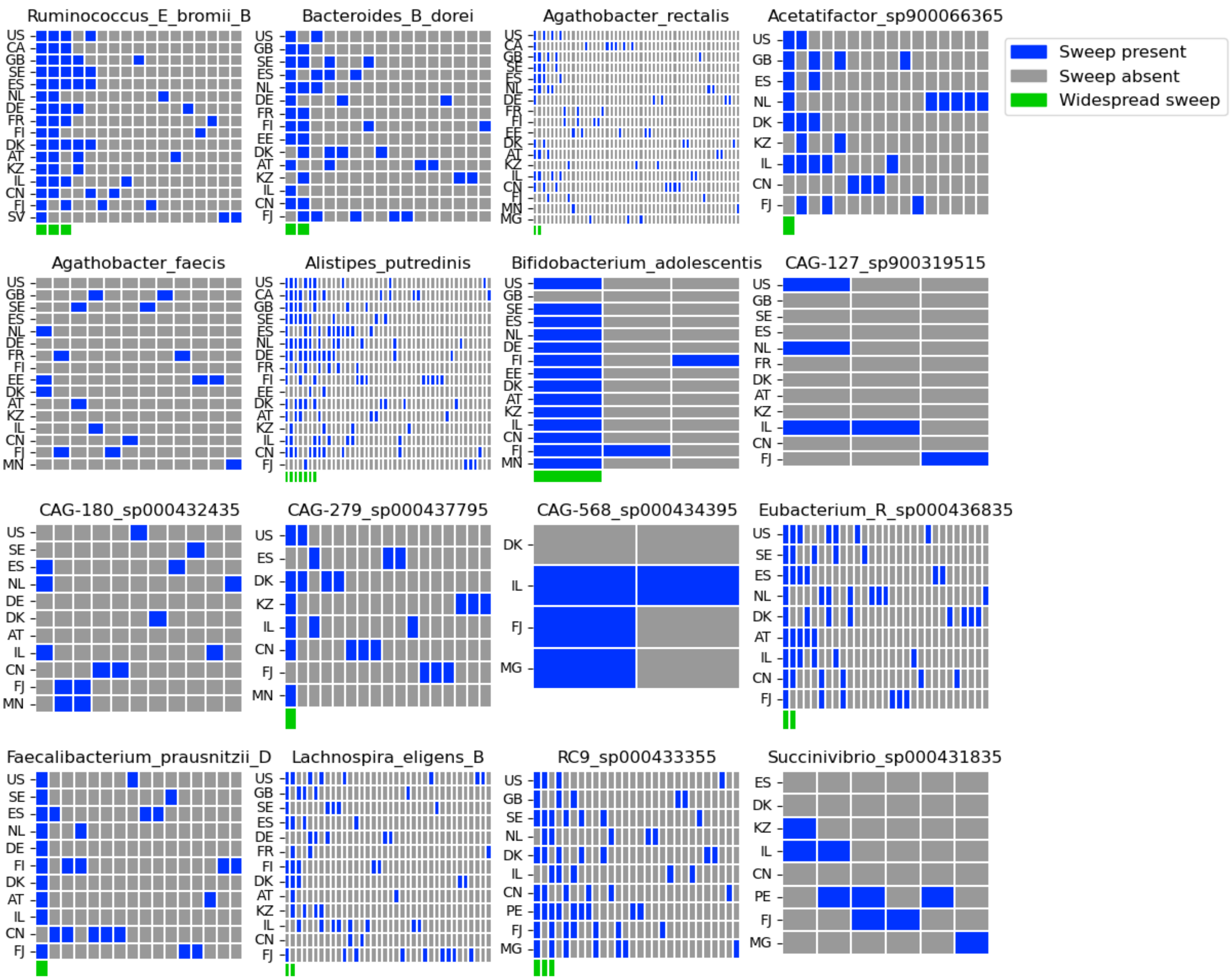

Figure S25: Presence and absence of sweeps across countries. Each heatmap shows the distribution of sweeps among populations that harbor a given species. Country codes are given on the y-axis, while each column corresponds to a unique sweep. Blue boxes indicate the presence of a sweep in a given population, while grey indicates its absence. Sweeps which are 'widespread'—that is, which have spread to a larger share of populations than expected by chance under a null model in which sweeps are randomly distributed among populations (Supplementary Section 5.1)—are highlighted with a green box below each column. The selective sweep at mdxEF in the species *R. bromii* (denoted "Ruminococcus_E_bromii_B" following the naming convention of Almeida et al. 2021[6]) is in the second column from the left of the top left subplot.

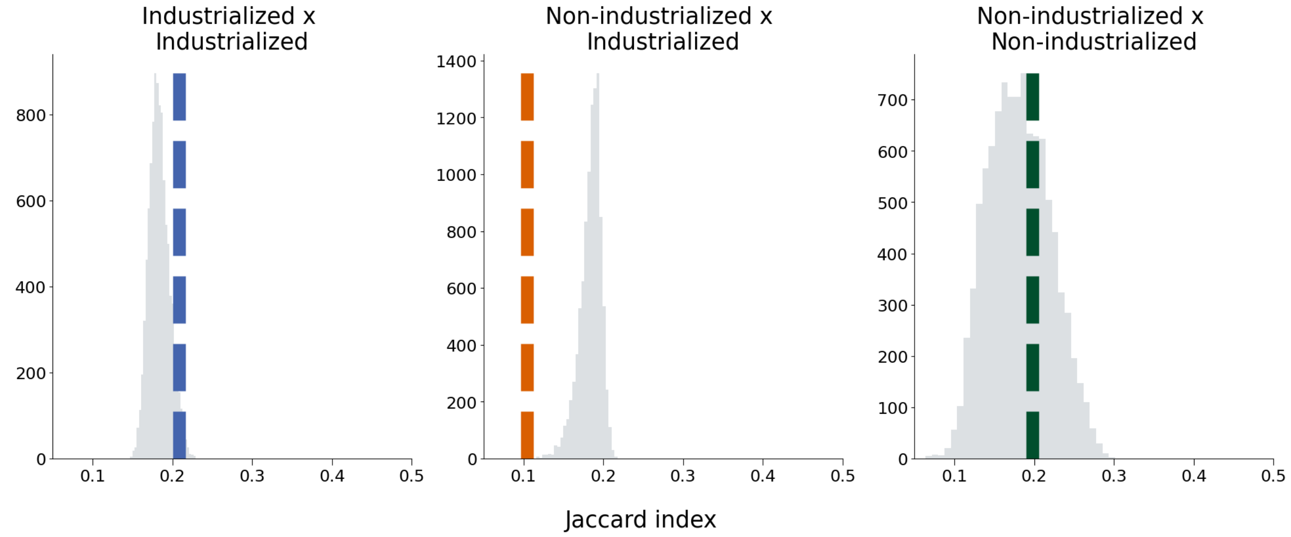

Figure S26: Jaccard permutation test. Distribution of Jaccard index after permuting population labels (grey distributions), compared with empirically measured Jaccard index (dashed colored lines). Industrialized populations share a greater proportion of sweeps with one another than expected by chance (p-value = *0.047*). Similarly, industrialized and non-industrialized populations share fewer sweeps than expected by chance (p-value $< 10^{-4}$). See Section 5.2 for further details.

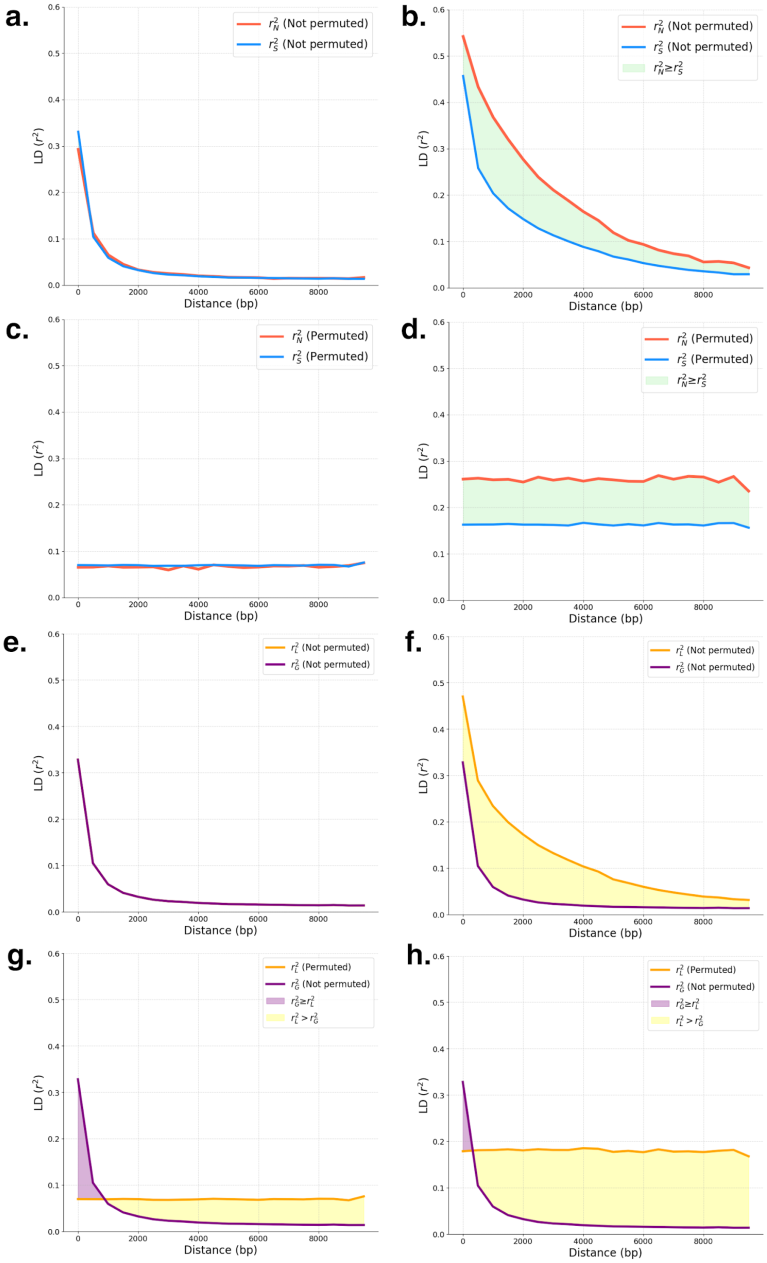

Figure S27: Impact of syntenic variation on $r^2_{\Delta NS}$ and $r^2_{\Delta LG}$ in simulated sweeps and neutrality. (A) r_N^2 and r_S^2 in a neutrally evolving region with preserved distances. **(B)** r_N^2 and r_S^2 in simulated sweeps with preserved distances (for all sweeps in this figure $s_B = 0.05$, $s_D = -0.001$, 0 generations since cessation of sweep). **(C)** r_N^2 and r_S^2 in a neutrally evolving region with permuted distances. **(D)** r_N^2 and r_S^2 in simulated sweeps with permuted distances. **(E)** r_L^2 and r_G^2 in a neutrally evolving region with preserved distances. **(F)** r_L^2 and r_G^2 in simulated sweeps with preserved distances. **(G)** r_L^2 and r_G^2 in a neutrally evolving region with locally permuted distances (genomewide distances are preserved). Overall, AUC(r_L^2) - AUC(r_G^2) exceeds 0, a false positive. **(H)** r_L^2 and r_G^2 in simulated sweeps with locally permuted distances (genomewide distances are preserved). Overall, AUC(r_L^2) - AUC(r_G^2) exceeds 0, a true positive.

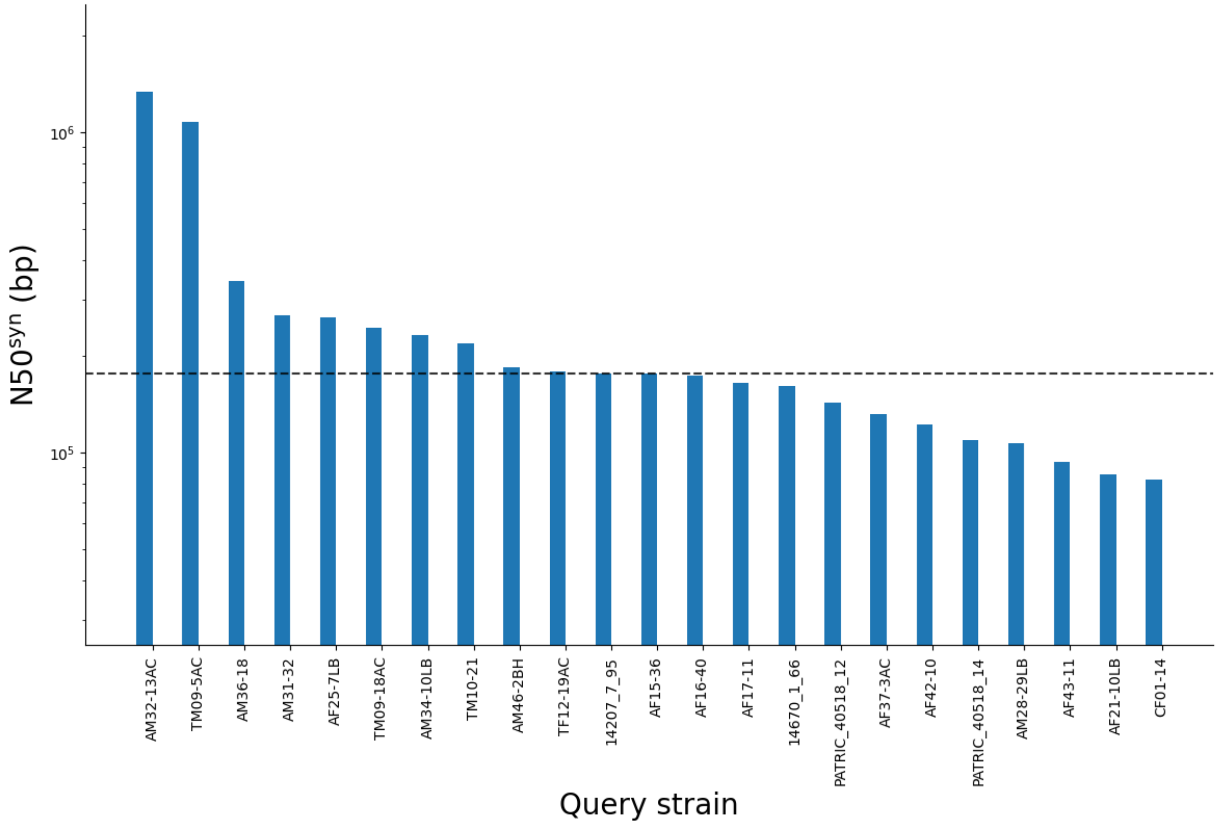

Figure S28: Distribution of N50^{syn} for *R. bromii* isolates. N50^{syn} is the distance such that 50% of the total subject assembly size is contained in runs of consecutive syntenic alignment blocks with the query strain of size N50^{syn} or greater. Median N50^{syn} is shown with the grey dashed line.

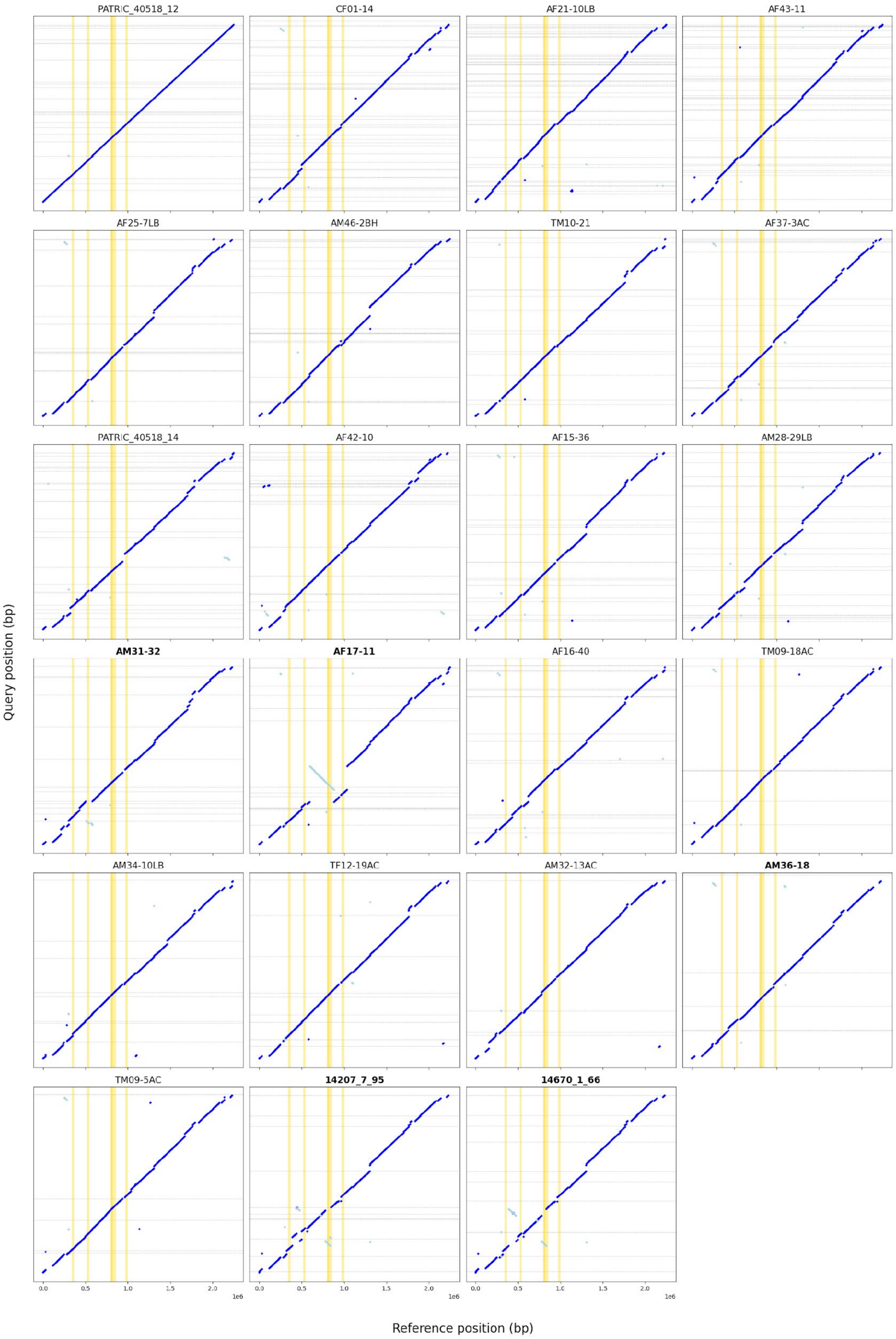

Figure S29: Synteny of *R. bromii* isolates with the reference genome used in the MIDAS database. Shown are dotplots of 23 *R. bromii i*solates against the single contig MIDAS reference genome (PATRIC ID: 657321.5). The x-axis gives the genomic position of each alignment block along the MIDAS reference genome, while the y-axis gives the position along each subject genome assembly contig, with contig boundaries denoted with grey bars. Dark blue lines show alignment blocks with forward orientation, while light blue blocks show regions aligned in the inverted orientation. Yellow bars show the locations of iLDS peaks detected across all 23

isolates. Each subplot's title corresponds to the ID of the strain being compared with the reference genome in the MIDAS database. Bolded titles connote strains exhibiting syntenic variation relative to the MIDAS reference genome around one or more peaks.

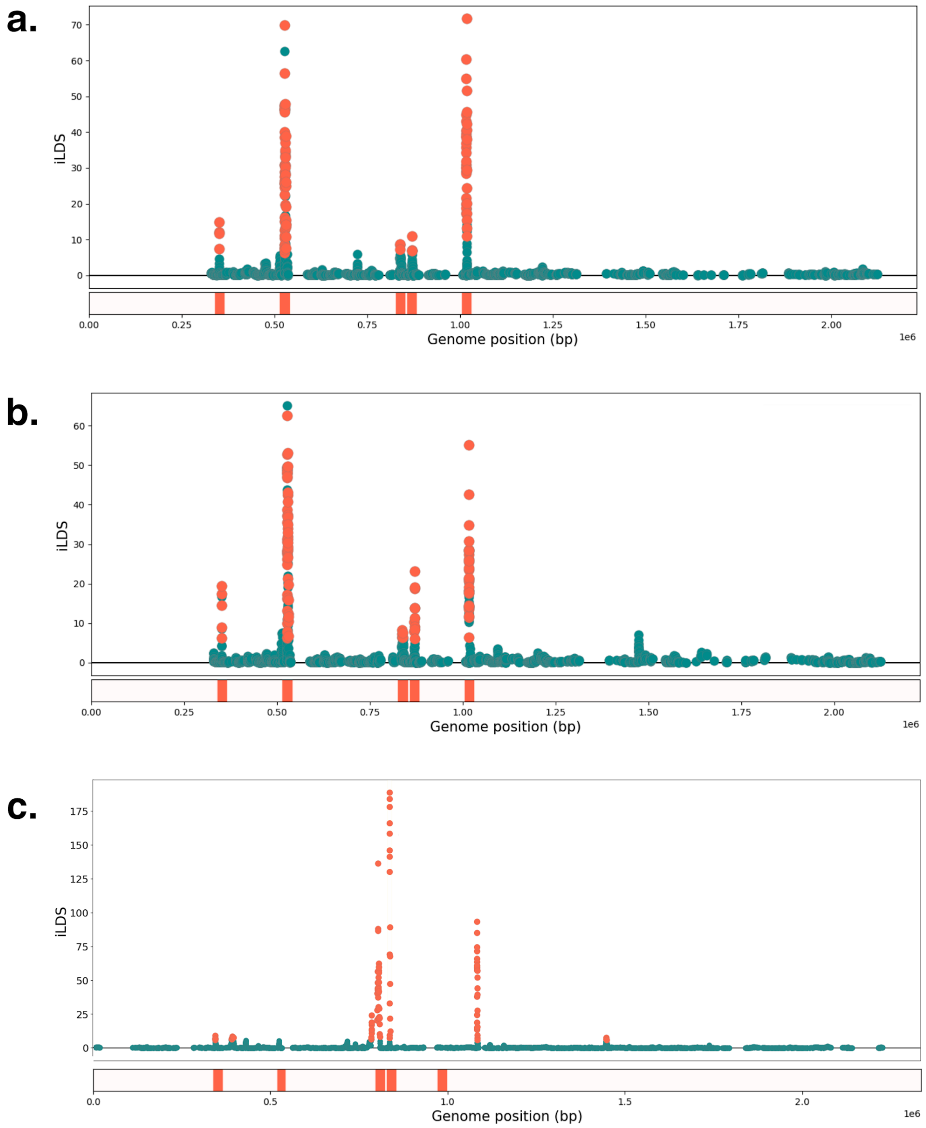

Figure S30: Comparison of iLDS scans run in *R. bromii* with vs without syntenic variation and across datasets. (A) iLDS for all isolates of *R. bromii* in UHGG. **(B)** iLDS scan for 18 isolates of *R. bromii* not exhibiting syntenic variation in any gene detected under selection in (A). The locations of the peaks in (A) and (B) are denoted by orange bars beneath each scan. While heights of peaks vary between (A) and (B), the identity of the peaks remains the same. **(C)** iLDS scan for *R. bromii* using quasi-phased genomes (see Figure 3B in Main Text). Here, the locations of the 5 peaks detected in (A) and (B) relative to the reference genome used in (C) are shown on the grid below the scan in orange bars. Three of 5 peaks present in (A) and (B) are also

present using the quasi-phasing approach, while a fourth peak (≈500kb) is present but not significant in (C) and a fifth peak (≈1mb) is entirely absent. As (C) relies on a different reference genome than (A) and (B), the locations of peaks were identified via blast search.

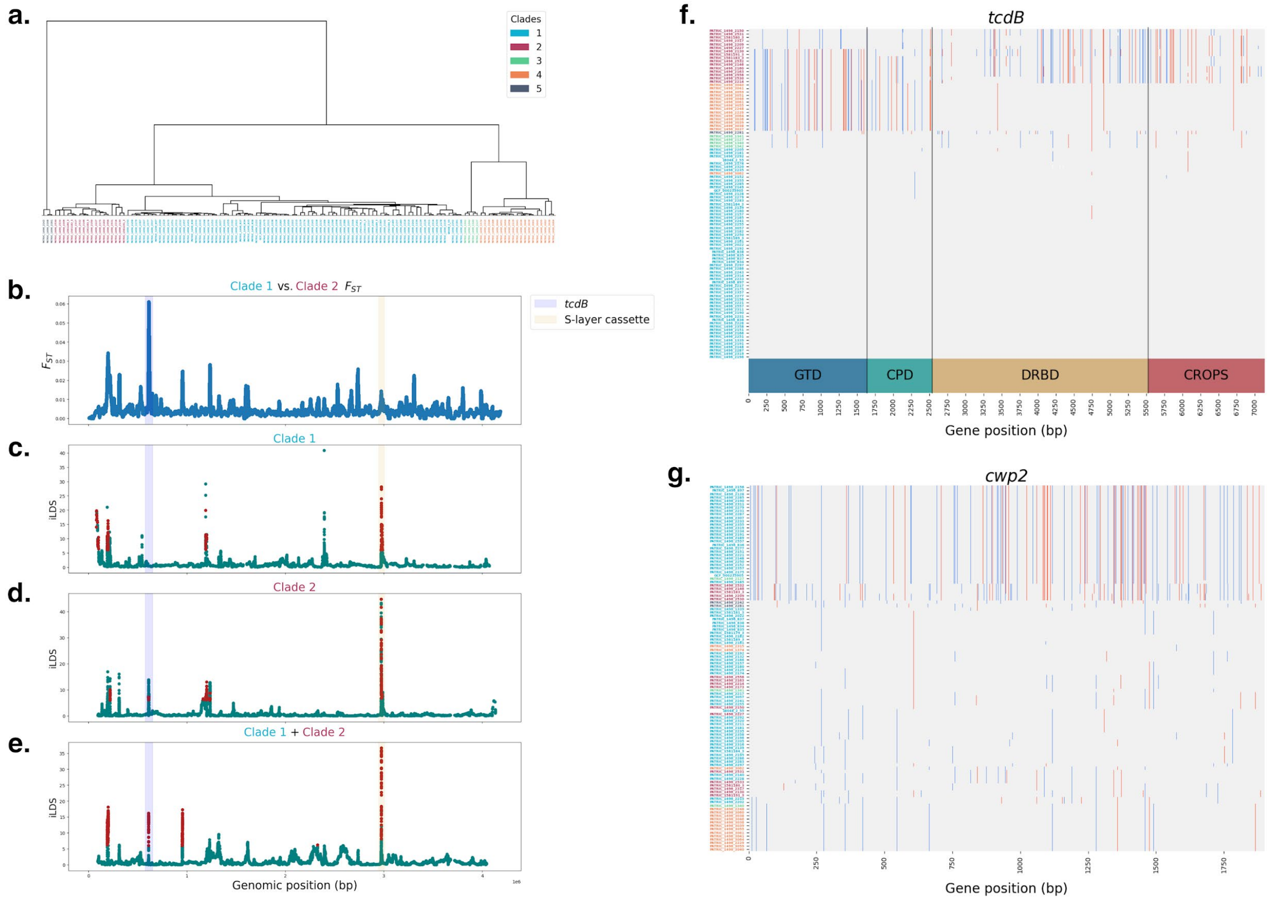

Figure S31: iLDS and *C. difficile* population structure. (A) UPGMA dendrogram made from pairwise divergence at 4D sites. Tips are colored by MLST clade inferred using PubMLST. **(B)** F_{ST} between Clade 1 and Clade 2, calculated in sliding 10kb windows. Here and in (C - E), the locations of the tcdB and the S-layer cassette loci are highlighted. **(C)** iLDS scan for isolates belonging to Clade 1 ($N = 63$). No peak is evident at tcdB but is evident at the S-layer cassette. **(D)** iLDS scan for isolates belonging to Clade 2 ($N = 16$). Peaks are evident at tcdB and the S-layer cassette. **(E)** iLDS scan for Clade 1 + Clade 2 ($N = 16 + 63 = 79$). Peaks are evident at *tcdB* and the S-layer cassette. **(F)** Haplotype diagram for tcdB. Isolate IDs are demarcated on the y-axis and colored based on their MLST clade. Positions along the *tcdB* gene are demarcated on the x-axis and are colored based on the domain of the protein. Grey indicates a strain is identical to the reference at that position, blue indicates a synonymous variant alternate allele, red indicates a non-synonymous variant alternate allele. **(G)** Haplotype diagram for the *cwp2* gene

which lies in the S-layer cassette. Axes are analogous to (F), though gene domains are not shown.

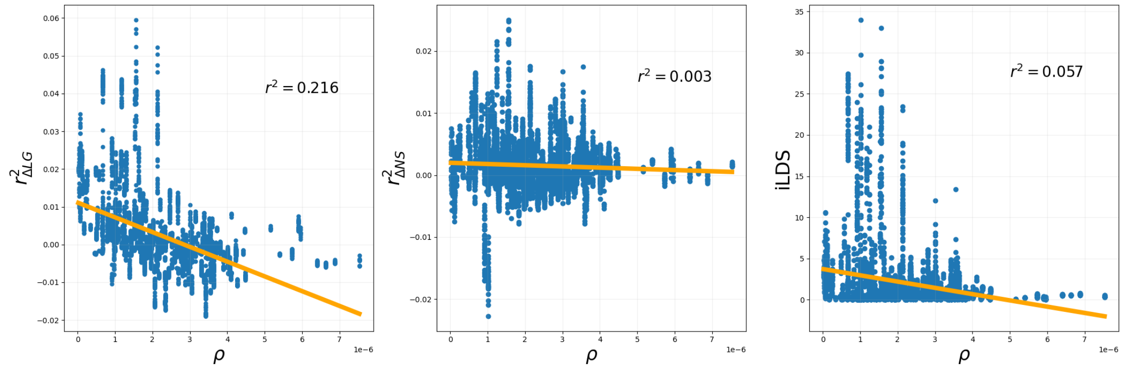

Figure S32: $r^2_{\Delta LG}$, $r^2_{\Delta NS}$, and iLDS as a function of recombination rate. Using the recombination rate map for *Drosophila melanogaster* chromosome 3R[17], we investigated the correlation between local recombination and $r^2_{\Delta LG}$, $r^2_{\Delta NS}$, and iLDS. While $r^2_{\Delta LG}$ shows a negative correlation with recombination rate—with regions of lower recombination showing higher overall LD, as expected—$r^2_{\Delta NS}$ showed minimal correlation with recombination rate (correlation coefficient of regression r^2 shown in panel). Consequently, iLDS shows moderate correlation, intermediate between $r^2_{\Delta LG}$ and $r^2_{\Delta NS}$, with local recombination rate. In each plot, the y-axis shows the mean values of $r^2_{\Delta LG}$/$r^2_{\Delta NS}$/iLDS in a 100kb window, while the x-axis gives the recombination rate in that window.

1 LD

1.1 LD calculations

To calculate r^2 between two loci, we used the standard formula:

$$r^2 = \frac{(f_{AB} - f_A f_B)^2}{(f_A(1-f_A) f_B(1-f_B))} \qquad \text{(S1)}$$

where f_A is the frequency of the minor allele at locus 1, f_B is the frequency at locus 2, and f_{AB} is the frequency of the AB haplotype. r^2 measurements were binned by genomic distance (bp) between the two loci, and mean r^2 within each bin was computed to determine decay in r^2 as a function of distance. We denote the location of the ith bin to be d_i, and the mean r^2 within this bin $\langle r_X^2(d_i)\rangle$.

1.2 AUC

To compute AUC(r_X^2)—the normalized area under the LD decay curve—we first approximate the total area under the curve using the trapezoidal rule and then divide by the total distance horizontal distance of the curve:

$$AUC(r_X^2) = \frac{1}{d_M - d_1} \sum_{i=1}^{M-1} \frac{\langle r_X^2(d_{i+1})\rangle - \langle r_X^2(d_i)\rangle}{2} (d_{i+1} - d_i) \qquad \text{(S2)}$$

where M is the total number of bins. The trapezoidal rule was implemented computationally with the trapz function in numpy[18].

1.3 LD confidence intervals

To quantify whether one LD curve is significantly elevated relative to another (e.g. r_N^2 versus r_S^2), we estimate confidence intervals (CIs) for the more elevated LD curve and assess if the area under the other LD curve is lower than the area under the lower CI. Once LD measurements are binned by distance and the mean value ($\langle r_X^2(d_i)\rangle$) and standard deviation ($\sigma^2_{r_X^2(d_i)}$) computed for each bin d_i, we compute a confidence interval for the sample mean within each bin:

$$CI(\langle r_X^2(d_i)\rangle) = \langle r_X^2(d_i)\rangle \pm z^* \frac{\sigma^2_{r_X^2(d_i)}}{\sqrt{n_i}} \qquad \text{(S3)}$$

where the z-score z^* sets the width of the interval used to assess significance, and n_i is the number of measurements in bin i. To determine if r_N^2 significantly exceeded r_S^2, we first computed a lower CI of r_N^2 (${r_N^2}^{(LCIB)}$) as follows:

$$r_N^2(d_i)^{(LCIB)} = r_N^2(d_i) - z^* \frac{\sigma^2_{r_N^2(d_i)}}{\sqrt{n_i}} \qquad \text{(S4)}$$

then calculated AUC(${r_N^2}^{(LCIB)}$) and compared this quantity with AUC(r_S^2). If AUC(${r_N^2}^{(LCIB)} - r_S^2$) > 0 (that is, if the total area between the lower bound of r_N^2 and r_S^2 is positive), we considered r_N^2 to be significantly elevated above r_S^2. When determining if r_N^2 exceeded r_S^2 at the whole-genome level (Main Text Figure 2B), significance was assessed at the $\alpha = 0.1\%$ level ($z^* = 3.291$). In essence, we assess whether r_N^2 overall is greater than r_S^2, on average, taking into account the variability of r_N^2 at each distance. It is important to note that in order for r_N^2 to significantly exceed r_S^2, it is neither necessary nor sufficient that r_N^2 be greater than r_S^2 in all bins. For rare variants, purifying selection is expected to decrease LD among non-synonymous variants relative to synonymous ones, and significance is therefore assessed with respect to the upper bound of r_N^2 (i.e. AUC(${r_N^2}^{(UCIB)}$) - AUC(r_S^2) < 0.)

To ensure that all species had comparably sized confidence intervals surrounding r_N^2 in our genome-wide assessment of significance, we downsampled the number of non-synonymous LD measurements to 1,000,000 for common variants (roughly 1,500 × 1,500 pairwise measurements) and 25,000,000 (roughly 7,000 × 7,000) for rare variants. Additionally, as the circular genome size in the species studied here varies, we only considered LD measurements of variants lying on the same contig and within 250kb of one another. LD was binned in 20 logarithmically spaced bins.

2 Simulations

To evaluate the conditions under which LD between non-synonymous variants might be elevated relative to LD between synonymous variants, we performed evolutionary simulations in SLiM 4.0[19] using custom recipes (Figures S1 -S 6).

While the species examined in the empirical portion of this study are haploid (with the exception of *D. melanogaster*, Section 10 below), simulations were performed with diploids. Diploid evolution was chosen as a simulation paradigm for two reasons. First, diploid simulations in SLiM employ a standard Wright-Fisher models of evolution, aiding in the interpretability of the simulation results. Second, we aimed to maximize computational efficiency for the large number of simulations we performed (*4500* burn-in simulations, *135,000* sweeps, *32,500* contractions) with population sizes of $N = 10^4$.

While haploid and diploid evolution differ in important ways, simulations were tailored to minimize the effect of these differences, while still retaining the performance advantages offered by simulations of diploidy in SLiM. First, all mutations were assigned a dominance coefficient of 1, making them fully visible to selection regardless of the presence or absence of any mutation on the opposite chromosome. In particular, this means that deleterious variants are always exposed to selection, as is the case with haploids. Second, while the crossing-over recombination typical of diploids differs from the homologous donation of tracts typical of bacterial evolution (at least in the core genome[16,20]), for our purposes these differences manifest primarily in the efficiency with which genomic elements at large distances can be unlinked. Variants that are physically close to each other in relation to the average recombined tract length l_r will be affected identically by crossing over versus homologous recombination (see Section 4.1). As individuals in our simulated populations have chromosomes of length 10kb, the decay of linkage should resemble that of a bacterial species with a tract length considerably larger than 10kb, such as *B. vulgatus* ($l_r \approx 20$kb).

To calculate LD, we first sampled 100 individuals from each simulated population. As with our genome-wide assessments, we next sampled a constant number of LD measurements between non-synonymous variants in our simulations to ensure that confidence intervals around r_N^2 were comparable across evolutionary scenarios. Specifically, for common variants, we sampled 1250 LD measurements between non-synonymous variants with replacement, for an average of one measurement per simulation for sweeps and contractions. Among rare variants, we sampled 1,000,000 LD measurements. We found, in general, that LD among rare variants was more variable (less smooth decay, wider confidence intervals) than LD among common variants for a given number of LD measurements, likely reflecting the more limited range of potential LD

values that can be taken on between pairs of rare variants. Pairs of singletons, for instance, may only take on r^2 values of zero or one.

If fewer than 100 LD measurements were available for a given parameter combination and mutation type, we did not calculate an LD decay curve (e.g. among non-synonymous variants for $[s_B = 10^{-5}, s_D = 10^{-1}]$, where the adaptive variant was always the only intermediate frequency non-synonymous variant following the sweep.) Following sampling, r_N^2 and r_S^2 were calculated using 8 bins each containing an equal number of non-synonymous variants. Significance was assessed in these simulations at the $\alpha = 0.1\%$ level, again mirroring the genome-wide measurements.

2.1 Burn-in/mutation-selection balance

In each replicate population of size of $N = 10^4$ individuals, burn-in proceeded for $10N$ generations, allowing the population to reach mutation-selection balance. Populations were constituted of individuals with diploid genomes of size $L = 10^4$ bp. Within each simulation, s_D was held constant at all non-synonymous sites, and between simulations, s_D was varied from a nearly-neutral regime ($N_e s_D << 1$: $s_D = 0, -10^{-5}, -10^{-4}$) through a weakly deleterious regime ($N_e s_D \approx 1$: $s_D = -10^{-3}$ to a strongly deleterious regime ($N_e s_D \gg 1$: $s_D = \{-10^{-2}, -10^{-1}\}$). Following the degeneracy of the codon table, we assumed a ratio of 2.31 non-synonymous sites per synonymous site in our simulations[10], with a per-base pair mutation rate $\mu = 10^{-6}$. Finally, the scaled recombination rate ρ/μ—i.e. the quotient of the recombination and mutation rates—was varied from 0.1 to 1 to 10 ($\rho = 10^{-7}, 10^{-6}, 10^{-5}$). For each parameter combination, 250 replicate populations were simulated.

2.2 Demographic contractions

To simulate demographic contractions, we began by first reading in a burn-in population with the desired s_D. In short, sharp contractions, the population size was reduced from $N = 10^4$ to $N = 10^3$, a 90% reduction, and the simulation was allowed to proceed for 20 generations. In the long, shallow contractions, the population size was reduced from $N = 10^4$ to $N = 5 \times 10^3$, a 50% reduction, and the simulation was then allowed to proceed for 5000 generations. For each burn-in population, five replicate contractions were performed, yielding a total of 1250 contraction simulations for each parameter combination.

2.3 Partial sweep

To simulate partial sweeps, we began by first reading in a burn-in population with the desired s_D, and then introducing a novel non-synonymous mutation at position zero on the chromosome. For each burn-in population, five replicate sweeps were performed, yielding a total of 1250 simulations per (ρ/μ, s_B, s_D) combination. Simulations proceeded until the focal, adaptive mutation reached 50% frequency, and were restarted from the burn-in state if the focal variant was lost from the population.

3 Data sources

In this work, we identify signatures of selection in haplotypes obtained from publicly available fecal shotgun metagenomes of individuals from around the world. We utilize two types of data to be able to ensure robustness of our approach. One type of data are 'quasi-phased' haplotypes, whereby a dominant haplotype is reconstructed from short read data with high confidence and minimal probability of error[1]. Another type of data are isolates and metagenome assembled genomes from the Unified Human Gastrointestinal Genome (UHGG) database)[6]. As shown for instance in Figure S30, both types of data yield replicable results. Below, we describe the pipeline used to reconstruct 'quasi-phased' haplotypes from short read metagenomic data, which replicates methodology from Garud *et al.* (2019)[1], and subsequently we describe our analysis of data from the UHGG.

3.1 Quasi-phased genomes

Raw sequencing reads for the 1,013 metagenomic samples used to construct quasi-phased genomes in these study were collected from 693 unique individuals (though only a single sample was used per person), including 250 individuals from the Human Microbiome Project (HMP)[21], a further 250 from Xie *et al.* (2016)[3], 185 from Qin *et al.* (2012)[4], and 8 from Korpela *et al.* (2018)[5]. The metadata for each sample are available in Table S1.

3.1.1 Estimation of species, gene, and SNV content of shotgun metagenomic samples

Species abundances, gene, and SNV content for each metagenomic shotgun sample were previously quantified in Garud *et al.* (2019)[1] using MIDAS (Metagenomic Intra-Species Diversity Analysis System, version 1.2, downloaded on November 21, 2016)[22]. Here we briefly summarize again the steps followed to obtain the data relevant for this work.

3.1.2 Estimation of species content

Species abundances for each sample were estimated by mapping reads to a set of single copy marker genes belonging to the 5,952 species in the MIDAS database. A species was considered present if it had an average marker gene coverage ≥ 3. Marker genes are defined as part of the MIDAS database. Since some hosts from the Human Microbiome Project dataset were sampled over multiple timepoints, we next determined a single reference database for each host by including all species present at one or more timepoints with coverage ≥3. In doing so, we were as inclusive as possible of any species present at any time point to avoid 'read donating' to species that may resemble true species that are present, but also selective enough to prevent 'read stealing' from a species truly present.

3.1.3 Estimation of CNV content

CNV content was next estimated by mapping reads to the pangenome for each species in each per-host reference database using Bowtie 2[23] with default MIDAS settings (local alignment, MAPID≥94.0%, READQ≥20, and ALN_COV≥0.75). MIDAS estimates the copy number of any gene (c) as the ratio between its coverage and the median single-copy marker gene coverage.

For each species, we identified 'core genes', defined as genes present in at least 90% of samples belonging to the largest clade (described in the Main Text and in Garud *et al.* (2019)[1]). Genes were deemed to be present in a sample if their copy number $c \leq 3$ and $c \geq 0.3$ (conditional on the mean single-copy number marker gene coverage of the sample being $\geq 5 \times$). In our calculation of LD, as well as for any iLDS scans, we only considered core genes. Since genes that are shared across species boundaries can result in read 'stealing' and 'donating', we excluded those genes part of a 'blacklist' of shared genes that we previously inferred[1]. Furthermore, since some genes may be absent from the MIDAS database, we also excluded genes with $c \geq 3$ in at least one sample in our cohort as in Garud *et al.* (2019)[1], to avoid examining genes that may have a high copy number due to their being present in multiple species.

3.1.4 Estimation of SNV content

Next, to estimate SNV content, reads were mapped to a single reference genome per species previously selected in the default MIDAS software. Read mapping was performed with Bowtie 2[23] using default MIDAS thresholds (global alignment, MAPID≥94.0%, READQ≥20, ALN_COV≥0.75, and MAPQ≥20). As per the defaults in MIDAS, species for a given sample were excluded from further analysis if ≤ 40% of their genome had any coverage or if they had median read coverage $D^{-} < 5$ at protein coding sites with nonzero coverage. To further avoid read stealing and donating, sites were masked in a given sample if $D < D^{-}/3$ or $D > 3D^{-}$, as these sites harbor coverage anomalously low or high compared to the genome-wide average coverage D^{-}. An additional coverage threshold requirement of 20 reads/site was imposed for calling SNVs for analyses below.

3.1.5 Quasi-phasing procedure

Once SNVs had been determined within each sample, we obtained 'quasi-phased' haplotypes from individual hosts following the procedure originally outlined in[1].

Briefly, quasi-phasing is a method that identifies samples with a single dominant strain so that alleles can be accurately assigned to that strain's haplotype. When multiple dominant strains coexist at intermediate frequencies within a host, there will be alleles present at intermediate frequencies that distinguish the two strains. The goal of strain phasing is to determine which allele at each position belongs to which strain haplotype. This can be challenging, as in general the probability of 'phasing errors' (in which an allele is wrongly imputed to a strain) increases when the frequencies of the strains are more similar. For example, in the example case where two strains coexist at exactly 50% frequency, it is impossible to determine which allele belongs to which strain.

Garud *et al.* (2019)[1] used two major criteria to select samples that can be confidently quasi-phased such that phasing errors are minimal:

1. Low numbers of intermediate frequency alleles: A low number of intermediate frequency alleles is indicative of the presence of a single dominant strain, while a large number is indicative of multiple dominant strains. Samples must have a low number of intermediate frequency ($0.2 < f < 0.8$) alleles relative to the mean number of differences expected between two divergent strains found in two random hosts.

2. High coverage: Quasi-phaseable samples must have high coverage ($\bar{D} > 20$), allowing for sufficient read support of alleles assigned to the quasi-phased haplotype.

With these criteria, Garud *et al.* (2019)[1] find that assigning major alleles with $f \geq 0.8$ to the dominant strain should result in mis-polarization rates less than 0.1 per genome. Any remaining intermediate frequency sites are treated as missing data. Further methodological details on quasi-phasing can be found in Garud *et al.* (2019)[1].

As our paper analyzes the same data as Garud *et al.* (2019)[1], we leveraged the same classifications of quasi-phaseable samples. In total, our analyses were performed on 2641 quasi-phased haplotypes belonging to the largest clade of each of the species considered, as seen in Figure S14.

3.1.6 Inclusion criteria

Once samples had been quasi-phased, we included only species that had a sufficient number of non-closely related ($d > 5 \times 10^{-4}$) haplotypes (described below in Section 3.1.7) and had a reference genome in MIDAS that was not fragmented into a large number of contigs. In particular, we included all species that had at least 20 non-closely related samples and an N50 > 10^5 bp (that is, species for which 50% or more of the reference genome assembly length is covered by contigs of at least 100kb). In total, 32 species passed these thresholds (see Figure S14).

3.1.7 Population structure

Several of the gut microbial species analyzed exhibit strong population structure[1,16,24], with ‘clades’ of lineages that recombine far more frequently with one another than with lineages belonging to other clades. In species with strong clade structure, LD is elevated genome-wide and shows comparatively little decay with distance[1,16]. Genetic diversity is therefore dominated by fixed differences between clades, masking signatures of ongoing recombination within clades. To control for population structure, we analyzed LD only among lineages belonging to the largest clade (as identified previously in Garud *et al.* (2019)[1]) of each species. In Supplementary Section 9, we analyze the performance of iLDS in a structured population.

Additionally, we controlled for the potential effects of elevated LD due to the presence of occasional closely related lineages, as measured by the per base-pair divergence d at fourfold degenerate sites (that is, sites where a nucleotide difference does not result in an amino acid change). Pairwise divergence d was determined by first calculating the number of fourfold degenerate sites at which two haplotypes differed, and then dividing this number by the total number of such sites in the core genome. While on average random pairs of lineages belonging to the same clade have d on the order of 10^{-2}/bp, unrelated hosts occasionally harbor very closely related lineages, with pairwise d two orders of magnitude smaller than typically observed across hosts[1,16]. To avoid elevations in LD arising from these anomalously closely related lineages, we choose only a single representative for any group of lineages $d < 5 \times 10^{-4}$/bp, as in our previous analysis[1].

3.2 UHGG

To augment our analyses using quasi-phased genomes, we downloaded alignments from the Unified Human Gastrointestinal Genome collection, which is made up of both metagenome assembled genomes (MAGs) and isolates. We wrote custom scripts to annotate each SNV falling in a coding region as synonymous or non-synonymous by using coordinates for the reading frame. Metadata for these samples, including accession numbers, can be found in Table S5.

Next, following the pipeline developed for metagenomic samples in[1], we identified top-level clades for each species we analyzed in UHGG by creating a UPGMA dendrogram of all samples using d at four-fold degenerate (4D) sites as a distance metric. Four-fold degenerate sites are sites at which all single nucleotide mutations yield the same amino acid. We then manually cut the dendrogram if there appeared to be multiple deep clades. As with HMP, we focused our subsequent analyses only on samples belonging to the largest clade identified, and retained only a single representative from each group of closely related samples ($d < 5 \times 10^{-4}$/bp).

To identify peaks present in multiple populations (Figure 4, Main Text), we only considered genes that were core to all continents for which a species was present. A gene was considered to be core if 90% of its polymorphic sites were present in 90% of non-closely related samples. Thus, our analyses only focused on genes that are truly present in all populations surveyed.

Species composition varies considerably between industrialized and non-industrialized microbiomes. We chose to focus only on species where 20 or more non-closely related genomes ($d > 5 \times 10^{-4}$/bp) at 4D sites, as in our analysis with quasi-phased genomes) were present in at least one industrialized and one non-industrialized population. As industrialized populations are far more extensively sampled, these populations tended to have considerably more genomes for a given species than did any non-industrialized population. Therefore, to ensure that each iLDS scan had the same sensitivity, we downsampled so that for each species, all populations had the same number of genomes.

For the pathogens analyzed (*C. difficile* and *H. pylori*), we did not make comparisons in the locations of peaks between continents, and therefore ran our analyses on all sites which were present in 90% or more of non-closely related samples, rather than defining core genes. Because these pathogens had large numbers of isolates ($O(10^2)$), we restricted our analyses only to isolate genomes to further minimize any source of mapping error.

3.2.1 Virulence factors

To identify virulence factors in *C. difficile* (Figure 3A, Main Text), we performed a blast search of its reference genome assembly from UHGG against the VFDB database[25]. We annotated any *C. difficile* gene as a virulence factor if it had more than 90% amino acid identity across ≥ 333 codons with a known VFDB virulence factor.

3.2.2 Identifying industrialized samples

To identify a sample as belonging to an industrialized or non-industrialized population, we relied on descriptions of the population lifestyles in the original source papers, looking for attributes such as food type and antibiotic exposure. Additionally, we checked for overlap with the annotations available in the curatedMetagenomicData package[26], which provides a range of

metadata on publicly available human gut microbiome metagenomic data. All samples from Fiji, Tanzania, Madagascar, Peru, and El Salvador were identified as non-Westernized in the curatedMetagenomicData package, while only a subset of samples from Mongolia were identified as non-Westernized. We excluded all Mongolian samples identified as Westernized from analysis.

4 iLDS

4.1 Determining the window size for computing iLDS

To perform an iLDS scan, LD is calculated in sliding windows across the genome, and LD measurements within each window subsequently binned by genomic distance between variants, so that AUC(r_N^2), AUC(r_S^2), and AUC(r_{local}^2) within each window may be calculated. To ensure that all windows contained both an adequate and comparable number of synonymous and non-synonymous variants with which to calculate r_N^2 and r_S^2 curves, windows were defined in terms of SNPs rather than base-pairs. Specifically, each window was defined by the region spanning n consecutive non-synonymous, intermediate frequency SNPs. The value n was chosen such that the average size, in base pairs, of all windows corresponded as closely as possible to the distance over which LD fully decays genome-wide for that species. To determine AUC($r_{genome-wide}^2$) in each window, we first downsampled to 1,000,000 LD measurements total, and then binned these measurements using the bins defined for that window.

To infer the distance over which LD decays, we model recombination as moving tracts of DNA from a donor genome into a recipient genome, where they replace homologous segments in a process known as homologous recombination in bacteria. While bacteria exchange genomic material in a variety of ways, including through the transfer of mobile genetic elements such as plasmids, homologous recombination is thought to be important in breaking up LD in the core genome, which is the focus of this work[16,20]. The size of these homologously exchanged tracts vary, but numerous experimental studies have found that, within a species, tract lengths are approximately exponentially distributed[20,27–29]. The speed at which loci are unlinked therefore depends crucially on the average size of these recombinant fragments (l_r), which in turn characterizes the total (exponential) distribution of tract lengths. In order to obtain the length scale on which LD decays (l_{DD}), we will first obtain an estimate of l_r, the average tract length.

In our model, two loci recombine when one or the other of the sites, but not both simultaneously, is homologously replaced by an imported fragment. Assuming a uniform per base-pair rate r at which a recombination event may begin at each site along the genome, and an exponential distribution of tract lengths with average length l_r, the effective recombination rate R between loci separated by a distance l is

$$R(l) = r l_r (1 - e^{-l/l_r}) \tag{S5}$$

This functional form suggests that the effective recombination rate increases linearly as $\sim rl$ at short distances (specifically, when $l \ll l_r$), and ultimately saturates to a single value rl_r for pairs of variants separated by $l \gg l_r$. The transition between these two regimes occurs when $l \approx l_r$.

In a neutrally evolving, panmictic population of size N, a classic result[30] states that expected LD obeys the following relationship:

$$E[r^2] = \frac{10+2NR}{22+26NR+4(NR)^2} \quad \text{(S6)}$$

where R is the effective recombination rate between sites, provided this expectation is taken with respect only to intermediate frequency alleles (e.g. $0.1 < f < 0.9$[30]). The ultimate cause of the decay of LD is the dependence of the effective recombination rate R on genomic distance—in our model, this relationship is given by Equation S5. We should in principle be able to estimate the parameters N, r, and l_r by fitting Equation S6 to the empirical LD decay curves for intermediate frequency synonymous variants (r_S^2). To obtain an estimate of the effective distance over which LD fully decays—and thereby a window size for iLDS—we are primarily interested in determining l_r.

While Equation S6 describes the behavior of $E[r^2]$ over all distances, its value is, in theory, independent of l_r over short distances ($l \ll l_r$) as $R(l)$ is itself independent of l_r in this regime. Therefore, as our goal is first to estimate l_r, we disregard short-distance LD and focus only on fitting when $l \approx l_r$ and greater. Using a completely different methodology, a previous study investigating the dynamics of recombination in human gut bacteria[16] estimated l_r for many of the species under consideration here, and obtained values which were $O(10^3) - O(10^4)$. Therefore, we restrict our attention to $l > 10^3$ bp.

In particular, we fit our r_S^2 curves with the following model:

$$E[r^2(l)] = C\frac{10+2NR(l)}{22+26NR(l)+4(NR(l))^2} \quad \text{(S7)}$$

where C is a normalization constant allowing us to fit the relative change in r_S^2 with distance, rather than absolute values of LD strictly. Fits were obtained using the scipy.optimize.curve_fit function in scipy[31].

To assess the performance of our model visually, we show the empirical r_S^2 and the fit obtained from Equation S7 in Figure S21. Overall, we see that the theoretical prediction (Equation S7) provides an excellent fit of LD across several orders of magnitude in the vast majority of species.

Having estimated l_r, we next move to the main goal of inferring l_{DD}, the distance over which LD fully decays genomewide. In our model of homologous recombination, the effective rate of recombination (and by extension, the level of LD) saturates in the $l \gg l_r$ limit of Equation S5—that is, as the distance between variants becomes large relative to the size of a tract that can be horizontally transferred. To infer this distance, we took the value of l_r estimated above as the mean of an exponential distribution of tract lengths for each species. We then took the 99th percentile of this distribution to be our estimate of l_{DD}.

As expected, the decay of LD appears to saturate to a constant value at large distances in all species examined (Figure S21). Moreover, by visual inspection, our estimated values of l_{DD} furnish a good approximation for this distance. The estimates of l_r and l_{DD} can be found in Table S3.

Next, to determine the window size n given l_{DD}, we calculated the median number of intermediate frequency non-synonymous SNPs spanning this distance. In general, this window size depends on both l_{DD} and the density of common non-synonymous SNPs, with larger l_{DD} and higher densities leading to greater n.

Finally, once the window size has been assessed, we determined the number of bins b used to bin LD within each window. We set b such that each bin would contain approximately 50 LD measurements—that is, $b = \binom{n}{2}/50$. To avoid creating windows with either very few bins (which obscures the distance dependence of LD within a window) or very many bins (which reduces statistical power), we set a minimum bin number of 3 and a maximum bin number of 10. Window sizes and bin numbers for each species are summarized in Table S3.

4.1.1 Comparison of tract length estimates with previous work

To assess the performance of our method for estimating the mean tract length (l_r) and the 99th percentile of the tract length distribution (l_{DD}), we compared our inferences of these quantities with those of Liu and Good (2024)[16]. The authors of this prior work explored the dynamics of homologous recombination in the same cohort of metagenomic samples examined here, employed quasi-phasing to obtain sample-specific haplotypes, and clade-controlled in an identical fashion. While the method developed by Liu and Good (2024)[16] detects recent transfers using pairwise comparisons between closely related strains, we infer statistical properties of the recombination process using the decay of LD among all non-closely related strains. Our approaches are complementary, and below we show a broad agreement between our estimates.

Liu and Good (2024)[16] identified ≈250000 unique instances of recombination—that is, the horizontal exchange of a genomic tract—across 29 species. The tract lengths for each these instances were provided in their supplemental Table S3. For each species analyzed in both datasets (28/29), we then computed the mean tract length (l_r) and the 99th percentile of the tract length distribution (corresponding to our l_{DD}) from the Liu and Good (2024)[16] empirical observations, and compared these quantities with those we obtained using our LD based method.

Overall, we found a correspondence between the two methods (Figure S23). Across all shared species, we found a correlation coefficient r^2 of 0.58 for l_r and 0.6 for l_{DD} between our approaches. The species for which our differing methodologies conflict most strongly is *Bacteroides caccae*, which Liu and Good (2024)[16] noted appears to have an anomalously low rate of recombination and large number of closely related lineages. Removing this single species increases the correlation coefficient r^2 for l_r to 0.7 and l_{DD} to 0.65.

4.2 Application of iLDS to data

iLDS is calculated in sliding windows across the genome, but in order for distances within each window to be well-defined, all windows are fully contained within a single contig in the reference genome assembly used for that species. Thus, windows never span contig boundaries in the reference genome. To ensure that contigs are much longer than the typical window size in which we calculated iLDS, we only ran scans on species with an N50 > 100, that is, species for which 50% or more of the reference genome assembly length is covered by contigs of at least 100. This threshold ensures that we only perform scans on species where LD can be calculated

across large contiguous regions. All contigs were mapped back to the same reference genome. When multiple contigs were present, the scan is presented as a concatenation across contigs (e.g. Main text Figure 3B).

In our scans, we only focused on core genes. Individual windows could span the boundaries of genes and operons, provided that they lay on the same contig in the reference genome; however iLDS is not calculated in intergenic regions, as only genes may contain non-synonymous polymorphisms.

Additionally, plasmids were also excluded from analysis. To do so, we used the software plasmidfinder[32] to identify any contigs in our reference assemblies which might correspond to a plasmid, so that such contigs could be formally excluded from downstream analysis. We identified plasmids in the reference assemblies of only three species: *Bacteroides xylanisolvens*, *Bacteroides eggerthii*, and CAG-127_sp900319515 (a novel species identified by MAG reconstruction in the UHGG dataset).

4.3 Identification of analysis windows with significant iLDS values

In order for a window to be called significant, three criteria must be met. First, iLDS must exceed some critical value. In simulations, we found that $\bar{r}^2_{\Delta(NS)}$ and $\bar{r}^2_{\Delta(LG)}$ each follow approximately standard normal distributions in the absence of positive selection (Figures S19). Therefore, iLDS should approximately follow a χ^2 distribution with two degrees of freedom. Sweeps, by contrast, produce iLDS values falling in the upper tail of the χ^2 distribution (Figures S18). Thus, we set the critical value of iLDS to be the upper α percentile of the χ^2 distribution (in this work, we employ an $\alpha = 5\%$). If iLDS exceeds this critical threshold, we additionally require that both $r^2_{\Delta NS}$ and $r^2_{\Delta LG}$ are significantly greater than zero at the same α threshold. Together, these criteria ensure that LD patterns within windows called as significant are consistent with selection.

4.4 Evaluating the performance of iLDS in simulations

We assessed the ability of iLDS to correctly identify selective sweeps, as well as the rate at which it misclassified variants not under positive selection as selected. To assess the potential for misclassifying non-sweeps as sweeps, we simulated constant N_e populations and two demographic contractions. To assess the ability of iLDS to correctly identify sweeps in a range of evolutionary parameters, we simulated sweeps with varied selection strengths as well as the number of generations since selection ceased to capture the decay of a sweep via mutation and recombination events over time.

We used the forward simulation engine SLiM to simulate populations at mutation-selection balance and selective sweeps[19]. These simulations are similar to those described in Supplementary Section 2, but are 500kb rather than 10kb, allowing us to assess iLDS's ability to detect sweeps of various strengths that have had the opportunity to sufficiently decay to roughly neutral levels at the edges. We simulated populations with an initial size of 10^4 individuals. As in the simulations outlined in Supplementary Section 2, we assumed a ratio of 2.31 non-synonymous sites per synonymous site, with per-base pair mutation rate $\mu = 10^{-6}$ and recombination rate $\rho = 10^{-6}$. For each evolutionary scenario described below, we simulated

1000 replicate populations. From each replicate population, we sampled 100 individuals, and calculated the value of iLDS based on this sample.

Based on our analyses in Supplementary Section 2, we performed these simulations in a parameter regime where we expect iLDS to be effective in detecting selective sweeps: that is, when non-synonymous variants are under weak purifying selection such that $s_D < s_B$. If purifying selection is very strong, we expect that few deleterious variants will hitchhike along with the focal adaptive variant during a sweep. Conversely, if purifying selection is weaker than drift, we do not expect differential patterns of LD between synonymous and non-synonymous variants to emerge during selective sweeps (Figure S5). In testing iLDS, we therefore modeled all non-synonymous variants (with the exception of the sweeping variant) as deleterious, with a single selection coefficient $s_D = -10^{-3}$ (i.e. $N_e|s_D| = 10$).

Before simulating selective sweeps or demographic contractions, we allowed each population to equilibrate to mutation-selection balance for $10N_e$ generations. Below we describe selective sweep and demographic contraction simulations.

4.4.1 Selective sweeps

We simulated selective sweeps of strength $s_B = 0.01, 0.05$, and 0.1, beginning after the population had reached mutation-selection balance. The adaptive variant was seeded at the midpoint of the genome. Simulations were restarted if the variant was lost from the population prior to reaching 50% frequency. After the beneficial variant reached a frequency of 50% in the population, the selection coefficient of the focal variant changed from beneficial to neutral. To assess the impact of aging on the ability of iLDS to detect sweeps, the sweep was allowed to decay for 0, 10, 100, 500, and 1000 generations.

4.4.2 Demographic contractions

As in Supplementary Section 2, we simulated two kinds of contractions: short, sharp contractions (90% reduction in population size for 20 generations) and long, shallow contractions (50% reduction for 5000 generations). These population size changes were introduced to populations equilibrated to mutation-selection balance.

4.4.3 Calculating iLDS

iLDS is applied in windows of a fixed number of intermediate frequency ($0.2 < f < 0.8$), non-synonymous SNPs. To identify the appropriate number of SNPs to use for each window, we computed the distance at which LD decays (l_{DD}) in simulations under mutation-selection balance. This corresponded to ~7.5kb, or 10 intermediate frequency non-synonymous SNPs. While sweeps may elevate LD locally in the region of the adaptive variant, we observed in our simulations of sweeps that on average across the whole genome, LD decayed at roughly the same rate as mutation-selection balance.

As expected, LD decays considerably more slowly in the contraction scenarios (~50kb). We therefore set the window size to be 40 intermediate frequency non-synonymous SNPs for the populations undergoing contractions, corresponding to this distance.

For each simulation, we first calculated $AUC(r_N^2 - r_S^2)$ and $AUC(r_{local}^2 – r_{genome-wide}^2)$ in each window as described in the Main Text:

$$r_{\Delta NS}^2 = AUC(r_N^2 - r_S^2) \quad and \quad r_{\Delta LG}^2 = AUC(r_{local}^2 - r_{genome-wide}^2)$$

and then normalized these quantities:

$$\bar{r}_{\Delta NS}^2 = \frac{r_{\Delta NS}^2 - E[r_{\Delta NS}^2]}{std(r_{\Delta NS}^2)} \quad and \quad \bar{r}_{\Delta LG}^2 = \frac{r_{\Delta LG}^2 - E[r_{\Delta LG}^2]}{std(r_{\Delta LG}^2)}$$

where $E[r_{\Delta NS}^2], E[r_{\Delta LG}^2], std[r_{\Delta NS}^2]$, and $std]r_{\Delta LG}^2]$ were computed from 1000 simulations at mutation-selection balance, where one value of $AUC(r_N^2 - r_S^2)$ and $AUC(r_{local}^2 - r_{genome-wide}^2)$ was drawn from each simulation at random.

However, for the populations experiencing demographic contractions, $E[r_{\Delta NS}^2], E[r_{\Delta LG}^2], std[r_{\Delta NS}^2]$, and $std[r_{\Delta LG}^2]$ were calculated from the simulations of contractions of the same magnitude.

To assess significance, we applied the criteria as described in Supplementary Section 4.3.

4.4.4 Simulation results

We plotted receiving operator characteristic (ROC) curves, which compare the true positive rate (TPR) of correctly identifying a selective sweep versus the false positive rate (FPR) of incorrectly classifying a neutral simulation as a selective sweep (Figures S7 - 9). To create the ROC curves, we calculated the TPR and FPR across a range of significance values, from $\alpha = 10^{-6}$ to $\alpha = 1$. Each of the three significance tests of iLDS was assessed at the same α value, with windows being called as significant if all three tests were significant (that is, does iLDS exceed the critical threshold, and are $r_{\Delta NS}^2 > 0$ and $r_{\Delta LG}^2 > 0$). Note that in Figure S10, we assessed true and false positive rates for $r_{\Delta NS}^2$ and $r_{\Delta LG}^2$ by assessing significance at each α threshold for these components individually.

Overall, iLDS is able to identify recent and strong selective sweeps. Specifically, when selection coefficients are sufficiently strong (e.g. $s_B = 0.05$ or 0.1), iLDS has the ability to detect sweeps that have decayed for up to 500 generations in our simulations. However, when selection coefficients are weak (e.g. $s_B = 0.01$), iLDS can only reliably detect sweeps that have decayed up to 100 generations in the past in our simulations. By 1000 generations in our simulations, iLDS typically struggles to distinguish sweeps. The performance of iLDS is marginally worse for populations experiencing bottlenecks, particularly sharp, short bottlenecks—however, this decrement in performance is mainly apparent for weak selection ($s_B = 0.01$, Figure S8).

Additionally, we computed false discovery rates (Figures S11 - 13). To do so, we set the critical value to match that of the value applied to data ($\alpha = 0.05$) for the χ^2 test of significance. We also required significant windows to have $AUC(r_N^2 - r_S^2) > 0$ and AUC($r_{local}^2 - r_{genome-wide}^2) > 0$. We computed the number of true positives identified in a set of 1000 simulations of selection and we computed the number of false positives in a set of 1000 simulations without selection. FDR was computed as the ratio of number of false positives vs the

total number of positives identified (i.e. $TP/(TP + FP)$). We found that in scenarios when iLDS has good power to detect selective sweeps, FDR rarely surpasses 10%.

4.5 Peak calling

After significant windows were identified, we next sought to localize individual gene-specific selective sweeps by identifying ‘peaks’ of elevated values of iLDS. To do so, we identified groups of physically proximate and tightly linked intermediate-frequency non-synonymous SNPs in the center of significant windows—that is, groups of non-synonymous SNPs consistent with having been carried together to intermediate frequency on shared recombinant tracts. Each SNP contained within a peak may be involved in the putative sweep as either a driver or passenger mutation.

We defined tight linkage as pairwise $r^2 \geq 0.5$ between SNPs. Among all pairwise LD comparisons between common, non-synonymous variants within distance l_{DD}, only 1.3% in *R. bromii* exceeded $r^2 = 0.5$, with similar proportions observed in other species. Thus, our linkage threshold ensures that variants within a peak are among the most tightly linked over this length scale.

To call peaks, we employed a greedy clustering algorithm which grouped tightly linked ($r^2 \geq 0.5$) and physically proximate (distance $\leq l_{DD}$) central SNPs in significant windows. First, we identified all pairwise comparisons between central SNPs in significant windows which met the linkage and proximity criteria described above. Next, we constructed a graph where nodes were individual central SNPs, and edges were drawn between tightly linked, proximate central SNPs. We then extracted the connected component containing the window with the largest iLDS value from this graph. Only connected components with more than one significant window were considered, otherwise solo significant windows were discarded from further peak-calling analysis. By filtering out clusters made up of only a single significant window, we conservatively ensure that all peaks are supported by multiple windows. While not all windows within a peak need be consecutive to one another, and central SNPs need not be tightly linked to all other variants in the peak, each is connected by an edge to at least one other significant window within the peak.

After identifying the peak with the highest iLDS value, we next removed from further peak calling analysis any significant windows within 25kb of any variant in the cluster. By doing so, we attempt to identify peaks that comprise independent selective events.

We then repeated this procedure—i.e. drawing our proximity/linkage graph, identifying the connected component containing the window with the highest iLDS value as a peak, and removing windows within 25kb of subsequent peaks—until all significant windows are either placed within a peak or removed from analysis.

To assign putative functions associated with each potential sweep, we then inspected gene annotations for all significant SNPs contained in a single peak. We report all such annotations in Table S4.

5 Distribution and overlap of sweeps among populations

5.1 Detecting widespread sweeps

Here we quantify whether sweeps are shared across multiple human populations more than expected by chance. To do so, we compare the observed distribution of sweeps to a null model in which all detected sweeps were distributed randomly across populations. Suppose a species is present in M populations and exhibits S unique peaks (i.e. S peaks appearing in one or more populations). Out of a maximum of MS possible sweeps, a total of S' sweeps are actually observed. If these S' sweeps were distributed randomly among populations, then the number of populations X in which a given sweep is detected would follow a binomial distribution:

$$P(X = k) = \binom{M}{k} p^k (1 - p)^{M-k}$$

where $p = S'/(MS)$. By extension, the probability of a sweep having spread to at least m populations under the null model is

$$P(X \geq m) = \sum_{k=m}^{M} \binom{M}{k} p^k (1 - p)^{M-k}.$$

We identify a sweep as 'widespread' if it is detected in m populations and m is such that

$$P(X \geq m) < \frac{0.05}{S}$$

where the denominator on the right hand side of the equation corrects for the fact that we are testing S hypotheses for each species.

In Figure S25, widespread sweeps are indicated by green boxes below the x-axis.

5.2 Jaccard index

To quantify the overlap in number of peaks between cohorts, we calculated the Jaccard index between all pairs of populations. The Jaccard index measures the overlap between two sets, and is the ratio of the size of the intersection of the sets to the size of the union of the sets.

$$J = \frac{\#elements\ shared}{\#elements\ total}$$

To determine the Jaccard index for a pair of populations, we first calculated the number of peaks shared and the number of peaks in total for each species. To determine a mean Jaccard index across industrialized populations, for example, we took the sum of the number of peaks shared across all species and all pairs of industrialized populations, and divided this quantity by the total number of peaks considered for each pair of populations. As the precise non-synonymous variants detected as significant by iLDS may vary, we counted peaks as shared between populations if a variant within one of the peaks was within 1000bp of a variant in the other, and not shared otherwise.

Next, to determine whether the value of the Jaccard index between each cohort was statistically significant, we performed a permutation analysis. First, we randomly shuffled the industrialized and non-industrialized status. Then, we repeated the Jaccard index calculation as described above 10^4 times. Finally, to obtain a (single-sided) p-value, we compared the true value of J for each pair of groups with the distribution of values of J for that pair, taking the p-value to be the frequency of permuted values more extreme than the observed value. Because the groups are of different sizes—with the industrialized/industrialized group containing nearly 10 times as many measurements as the Non-industrialized/Non-industrialized group—the permuted distributions have different variances (though identical means). Histograms showing the distributions of permuted values relative to the true values of the Jaccard index can be found in Figure S26.

6 Gene enrichment analysis

To assess if particular categories of genes were enriched for signatures of selection, we used gene annotations from the PATRIC database and performed several gene enrichment analyses on different classes of annotations[33,34]. First, we collated a list of all genes which lay within a peak in any of the 32 species for which we ran a scan (Table S4). Next, we performed separate enrichment analyses on three types of annotations, (i) predicted gene products[33], (ii) enzyme commission numbers[8], and (iii) COG categories[7]. The MIDAS reference database already contained predicted gene product and enzyme commission number annotations from PATRIC[33]. To obtain COG categories for each gene, we annotated the reference genomes in MIDAS using eggnog-mapper[35].

To assess whether a functional category was significantly enriched among selected genes, we performed a single-sided Fisher exact test comparing the number of these classes of genes (i.e. (i), (ii), & (iii), above) detected to be under selection with the number of such genes found among all core genes, and corrected for false discovery using the Benjamini-Hochberg procedure. After false discovery correction, we assessed significance at the $\alpha = 5\%$ level.

Observed versus expected counts of functional categories among genes under selection, as well as statistical significance, is summarized in Extended Data Figure 7.

7 Comparison of iLDS with previous selection scans in data

To directly assess the performance of iLDS relative to other scans for selection, we compared the results of our iLDS scan on *C. difficile* with iHS[36], Tajima's D[37], and d_N/d_S. The results of this comparison of scans is shown in Extended Data Figure 2.

iHS is designed to follow an approximately standard normal distribution, and we therefore used the 99th percentile of the normal distribution (≈ 2.32) as a cut-off value of iHS to identify outlier windows which may be candidates for selective sweeps. To calculate iHS, we used the implementation found in the scikit-allel package[38], specifically the allel.ihs() function. We normalized iHS within 10% allele frequency bins using the allel.standardize_by_allele_count() utility.

We likewise used scikit-allel (allel.windowed_tajima_d()) to calculate Tajima's D in sliding 1kb windows spaced at 100bp intervals along the genome.

d_N/d_S was calculated on a per-gene basis. Specifically, for each gene, we summed the total number of SNP differences at 1D and 4D sites between each pair of strains. We then divided these sums by the total possible number of opportunities for 1D and 4D SNP differences—that is, the total number of 1D or 4D sites in the gene multiplied by the number of pairwise comparisons between strains. Genes with fewer than five 4D SNPs were excluded from analyses.

8 Robustness of iLDS to synteny

For iLDS to meaningfully reflect patterns of LD along the genome, genomic distances between SNPs must be locally well-defined. Throughout this work, distances between SNPs are determined with respect to a single reference genome for each species. While we restrict our attention to SNPs in the core genome, differences in synteny as well as presence/absence of accessory genes between the reference genome and other genomes may alter the relative positions of sites on the genome. In general, variants that are physically closer to one another on some genomes than on the reference genome will exhibit abnormally high LD, and likewise, variants that are physically farther away from one another will have lower than expected LD. Here we investigate the impact of syntenic variation on iLDS.

First we investigate the effects of syntenic variation on iLDS in simulations. To assess the susceptibility of each of iLDS's components to false negatives and false positives induced by syntenic variation, we computed values of $r^2_{\Delta NS}$ and $r^2_{\Delta LG}$ in simulations in scenarios where synteny is preserved vs destroyed for both simulations of sweeps and neutral evolution. To destroy all synteny, we randomly permuted distances between SNPs in the vicinity of a focal 10kb region surrounding the sweeping SNP (for sweeps) or in a randomly chosen 10kb region (for neutrality). We found that for sweeps, $r^2_N > r^2_S$ both when distances are preserved (Figure S27B) and permuted (Figure S27D), indicating that $r^2_{\Delta NS}$ is still sensitive to sweeps when there is syntenic variation. Conversely, for neutrality, we found that $r^2_N = r^2_S$ both when distances are preserved (Figure S27A) and when permuted (Figure S27C), indicating that $r^2_{\Delta NS}$ is not artificially elevated under neutrality when there is syntenic variation. Different patterns emerge for $r^2_{\Delta LG}$, however. In the case of sweeps, r^2_L is elevated over a neutral genomewide baseline (r^2_G) both when distances are locally preserved (Figure S27F) and permuted (Figure S27H), indicating $r^2_{\Delta LG}$ can maintain power to detect sweeps even when there is syntenic variation. However, under neutrality, r^2_L is elevated above r^2_G in the permuted scenario (Figure S27G) but not when distances are preserved (Figure S27E), indicating that $r^2_{\Delta LG}$ alone can produce false positives when there is syntenic variation.

Altogether, these simulation results indicate that syntenic variation does not introduce systematic bias into $r^2_{\Delta NS}$, as changes in synteny affect the distances between synonymous and non-synonymous variants equivalently, on average. However, syntenic variation can produce falsely elevated values of $r^2_{\Delta LG}$, even in the absence of selection. Thus, we expect distortions to iLDS due to syntenic variation to arise in the $r^2_{\Delta LG}$ rather than the $r^2_{\Delta NS}$ component. However, the requirement that $r^2_{\Delta NS}$ significantly exceed zero in order for a peak to be called makes it unlikely (though not impossible) that iLDS will be falsely significant due to syntenic variation alone. As

seen previously for both demographic contractions and recombination rate variation, $r^2_{\Delta NS}$ buffers against potential false positives due to syntenic variation, even as $r^2_{\Delta LG}$ provides additional sensitivity to detect selective sweeps (Figures S1-3).

Next, we assessed the extent of syntenic variation and its effects on iLDS output in real data from a single species. First, we assessed syntenic variation between 24 strains of *R. bromii*. These include the reference genome in the MIDAS database[22], which was used to perform quasi-phasing, as well as the other 23 *R. bromii* isolates available in the UHGG dataset[6] (for accession numbers see Supplementary Table S6). To assess the relative positions of aligned regions between each of the 23 isolates from UHGG and the MIDAS reference genome (PATRIC ID: 657321.5), which is assembled in a single contig, we used nucmer[39] to perform pairwise alignment of each isolate in UHGG against the MIDAS reference. We visualized these pairwise alignments as 'dotplots' (Figure S29)[40].

For *R. bromii*, iLDS is calculated in genomic windows of ≈13kb, on average. To better understand the extent of syntenic variation within windows, we assessed the typical length scale over which synteny is conserved between pairs of strains. To do so, we first identified runs of consecutive alignment blocks exhibiting identical synteny and orientation for each query-subject pair. Next, we calculated the genomic distance $N50^{syn}$ such that 50% of the total subject assembly size is contained in runs of consecutive syntenic alignment blocks of size $N50^{syn}$ or greater. Across all strains, we found a median $N50^{syn}$ of 175kb, indicating that for a typical subject strain, the majority of the genome is contained within large regions of exact synteny with the query strain—in particular, $N50^{syn}$ for *R. bromii* exceeds the typical iLDS window size by more than an order of magnitude. We note that the $N50^{syn}$ we calculated here is likely an underestimate of the true extent of synteny, as subject strain genome assemblies are fragmented into many contigs, limiting the maximum length of consecutive syntenic blocks we can infer. Overall, syntenic breaks generally occur over a length scale far greater than the iLDS window size.

By visual inspection, we identified five strains exhibiting syntenic variation relative to the MIDAS reference genome in one or more genes contained within peaks identified by iLDS (Figure S30, bold titles). Four strains (AM31-32, AF17-11, 14207_7_95, and 14670_1_66) exhibited inversions encompassing iLDS peaks, while one strain (AM36-18) exhibited a partial deletion of one gene within an iLDS peak. To directly assess the impact of syntenic variation on the output of iLDS, we removed these five strains and re-ran our scan. We found that peaks were identical when these strains were excluded (Figure S30). We conclude that the peaks we identify in *R. bromii* do not appear to be driven by distance distortions introduced by syntenic variability between strains.

Lastly, we confirm the robustness of the iLDS scan results with respect to choice of reference genome by comparing scans conducted among UHGG isolates vs the results obtained from out quasi-phasing approach. We find that 3 of the 5 peaks identified using UHGG isolates were also present using the alignment-based quasi-phasing approach, despite the reference strain differing between the UHGG data and the MIDAS reference database. Overall, the agreement between the locations of iLDS peaks between scans including strains with and without syntenic variation in the relevant genomic regions, and between independently collected datasets relying on different reference genomes.

9 iLDS performance in structured populations

Population structure can profoundly affect adaptive evolution in bacteria and other organisms, as different, independent selective sweeps can occur in different subpopulations in the face of structure. F_{ST} is a standard measure of inter-population genetic differentiation, and substantially elevated F_{ST} values are often used to infer adaptive genetic divergence between populations in bacterial species[41,42]. To assess the performance of iLDS in detecting sweeps in a structured population, we investigated the relative abilities of iLDS and F_{ST} to detect selection within and between clades of the species *C. difficile*, which is known to be composed of five major clades[43,44]

Several previous studies have demonstrated that evolution at the positively selected *tcdB* locus in *C. difficile* is strongly constrained by the background population structure, with each haplotype of *tcdB* largely or entirely restricted to a single clade[43,44]. By contrast, different alleles of the S-layer cassette have been shown to spread frequently between strains in different clades through horizontal gene transfer[45–47]. We therefore specifically investigated the relative abilities of iLDS and F_{ST} to detect selection within and between clades at loci which are either strongly (*tcdB*) or weakly (S-layer cassette) constrained by background population structure.

First, we assigned the *C. difficile* isolates available in UHGG to previously established multi locus sequence type (MLST) clades using PubMLST (Figure S31A) [43,44]. When we compared patterns of divergence between the two largest clades (Clade 1 and Clade 2) across the genome using F_{ST}, we found that the highest peak of F_{ST} overlapped *tcdB*, consistent with adaptive differentiation between clades at this locus (Figure S31B). By contrast, F_{ST} was far less peaked at the S-layer cassette, indicating that inter-clade diversification is less significant at this locus.

Next, we performed iLDS scans for Clade 1 and Clade 2 separately (Figures S31C-D), and for the two clades jointly (Figure S31E). We found that iLDS does not detect *tcdB* as under selection within Clade 1, but does detect it as under selection within Clade 2 and also in the combined Clade 1 and Clade 2 population. The *tcdB* haplotype diagram shown in Figure S31F demonstrates that Clade 1 is fixed for its own *tcdB* haplotype, while Clade 2 harbors two *tcdB* haplotypes at intermediate frequency with one another. While there is little evidence of inter-clade recombination of the entire *tcdB* locus given the tight association between *tcdB* haplotype and clade assignment, the two dominant Clade 2 *tcdB* haplotypes appear to differ from one another. This is likely due to a recombination event that preserved the C-terminal divergent receptor binding (DRBD) and CROPs domains while overwriting the N-terminal glucosyltransferase (GPD) and cysteine protease (CPD) domains[43,44]. Taken together, the results of the iLDS scans and the structure of the haplotypes at *tcdB* appear consistent with a model in which selection has fixed adaptive differences at *tcdB* between clades, and where a selective sweep of a recombinant allele is ongoing within Clade 2.

By contrast, using iLDS the *cwp2* gene (which falls in the S-layer cassette) is detected as under selection both within each clade individually, and also in the combined population (Figure S31C-E). This likely is because the sweep at *cwp2* is evidently far less constrained by the background population structure, as the entire *cwp2* locus has recombined multiple times between clades (Figure S31G).

Altogether, this analysis indicates that similar to F_{ST}, iLDS can identify instances where selection is driving adaptive differentiation between clades. However, compared to F_{ST}, iLDS is much better able to identify instances where sweeps are occurring within clades or where adaptive alleles recombine frequently across clades.

10 Analyses with *Drosophila melanogaster*

Though the primary focus of this work is detecting selective sweeps in bacteria, hitchhiking of linked deleterious variants with selected sites is a general evolutionary phenomenon that many species experience[48–52]. If hitchhiking is an important factor elevating r_N^2 above r_S^2, we expect this signal to manifest in a very broad range of adapting populations. Therefore, in addition to our scans in gut bacteria, we further assessed the capacity of iLDS to recapitulate known sweeps in natural populations of *Drosophila melanogaster*, focusing in particular on three loci implicated in pesticide and virus resistance on chromosomes 2R (*Cyp6g1*) and 3R (*Ace*, *CHKov1*)[53–56].

We analyzed alignments of 100 *D. melanogaster* genomes from Raleigh, North Carolina[57] as well as 100 *D. melanogaster* genomes from a putative ancestral population in Zambia[58]. These genomes were chosen for analysis in Harris *et al.* (2023)[59] based on having identity by descent levels <20% with any other genome and having the least amount of missing data in the overall data set.

To identify an analysis window, we visually estimated that LD among common synonymous variants fully decayed by ≈300kb on both chromosomes and used this distances to determine the window sizes on these chromosomes for both the Raleigh and Zambia populations (Extended Data Figure 4).

We found that in the North Carolina population, our scans were sharply peaked around the three known instances of selective sweeps at *Cyp6g1*, *Ace*, and *CHKov1*, Extended Data Figure 4). By contrast, in the Zambian population, the peaks were not significant, consistent with previous work[60–62] showing that these loci are under diminished or not under selection in this population. While the sweeps at *Cyp6g1* and *CHKov1* are both driven by transposable element insertions, the sweep at *Ace* is a result of three single nucleotide mutations in that gene[53,54]. When we applied our peak-calling procedure, in which groups of significant windows centered around variants which are both tightly linked and physically close to one another are considered a peak (Section 4.5), we found that the peak in chromosome 3R near *Ace* (Extended Data Figure 4) was, in fact, made up of two variants lying in *Ace* and one other nearby variant. However, the peaks lying near *Cyp6g1* in chromosome 2R and *CHKov1* in chromosome 3R did not contain any SNPs lying in these genes, consistent with these sweeps being driven by transposable element insertions which are not directly visible to iLDS, which computes LD and calls peaks using SNPs alone.

While we only tested iLDS's ability to identify simulated single mutational origin selective sweeps (hard sweeps), the sweeps at *Cyp6g1*, *Ace*, and *CHKov1* in the Raleigh population are all thought to involve multiple, independent mutational origins (soft sweeps)[53,54]. The ability of iLDS to successfully identify these soft sweeps may indicate that the statistic has power to identify both hard and soft selective sweeps. Future work testing the capacity of iLDS to uncover

selective sweeps under a range of adaptive scenarios — including hard versus soft sweeps — is needed.

Works cited

1. Garud, N. R., Good, B. H., Hallatschek, O. & Pollard, K. S. Evolutionary dynamics of bacteria in the gut microbiome within and across hosts. *PLoS Biology* **17**, e3000102 (2019).

2. Lloyd-Price, J. *et al.* Strains, functions and dynamics in the expanded human microbiome project. *Nature* **550**, 61–66 (2017).

3. Xie, H. *et al.* Shotgun metagenomics of 250 adult twins reveals genetic and environmental impacts on the gut microbiome. *Cell Systems* **3**, 572–584 (2016).

4. Qin, J. *et al.* A metagenome-wide association study of gut microbiota in type 2 diabetes. *Nature* **490**, 55–60 (2012).

5. Korpela, K. *et al.* Selective maternal seeding and environment shape the human gut microbiome. *Genome Research* **28**, 561–568 (2018).

6. Almeida, A. *et al.* A unified catalog of 204,938 reference genomes from the human gut microbiome. *Nature Biotechnology* **39**, 105–114 (2021).

7. Tatusov, R. L., Galperin, M. Y., Natale, D. A. & Koonin, E. V. The COG database: A tool for genome-scale analysis of protein functions and evolution. *Nucleic acids research* **28**, 33–36 (2000).

8. Barrett, A. J. Enzyme nomenclature. Recommendations 1992: Supplement 2: Corrections and additions (1994). *European Journal of Biochemistry* **232**, 1–1 (1995).

9. Hill, W. G. & Robertson, A. The effect of linkage on limits to artificial selection. *Genetics Research* **8**, 269–294 (1966).

10. Garcia, J. A. & Lohmueller, K. E. Negative linkage disequilibrium between amino acid changing variants reveals interference among deleterious mutations in the human genome. *PLoS Genetics* **17**, e1009676 (2021).

11. Good, B. H. Linkage disequilibrium between rare mutations. *Genetics* **220**, iyac004 (2022).

12. Sohail, M. *et al.* Negative selection in humans and fruit flies involves synergistic epistasis. *Science* **356**, 539–542 (2017).

13. Sandler, G., Wright, S. I. & Agrawal, A. F. Patterns and causes of signed linkage disequilibria in flies and plants. *Molecular Biology and Evolution* **38**, 4310–4321 (2021).

14. Lee, Y. C. G. Synergistic epistasis of the deleterious effects of transposable elements. *Genetics* **220**, iyab211 (2022).

15. Ragsdale, A. P. Local fitness and epistatic effects lead to distinct patterns of linkage disequilibrium in protein-coding genes. *Genetics* **221**, iyac097 (2022).

16. Liu, Z. & Good, B. H. Dynamics of bacterial recombination in the human gut microbiome. *Plos Biology* **22**, e3002472 (2024).

17. Comeron, J. M., Ratnappan, R. & Bailin, S. The many landscapes of recombination in drosophila melanogaster. (2012).

18. Harris, C. R. *et al.* Array programming with NumPy. *Nature* **585**, 357–362 (2020).

19. Haller, B. C. & Messer, P. W. SLiM 4: Multispecies eco-evolutionary modeling. *The American Naturalist* **201**, E127–E139 (2023).

20. Förster, M., Rathmann, I., Yüksel, M., Power, J. J. & Maier, B. Genome-wide transformation reveals extensive exchange across closely related bacillus species. *Nucleic Acids Research* **51**, 12352–12366 (2023).

21. A framework for human microbiome research. *Nature* **486**, 215–221 (2012).

22. Nayfach, S., Rodriguez-Mueller, B., Garud, N. & Pollard, K. S. An integrated metagenomics pipeline for strain profiling reveals novel patterns of bacterial transmission and biogeography. *Genome Research* **26**, 1612–1625 (2016).

23. Langmead, B. & Salzberg, S. L. Fast gapped-read alignment with bowtie 2. *Nature Methods* **9**, 357–359 (2012).

24. Costea, P. I. *et al.* Subspecies in the global human gut microbiome. *Molecular Systems Biology* **13**, 960 (2017).

25. Liu, B., Zheng, D., Zhou, S., Chen, L. & Yang, J. VFDB 2022: A general classification scheme for bacterial virulence factors. *Nucleic acids research* **50**, D912–D917 (2022).

26. Pasolli, E. *et al.* Accessible, curated metagenomic data through ExperimentHub. *Nature methods* **14**, 1023–1024 (2017).

27. Croucher, N. J., Harris, S. R., Barquist, L., Parkhill, J. & Bentley, S. D. A high-resolution view of genome-wide pneumococcal transformation. *PLoS Pathogens* **8**, e1002745 (2012).

28. Mell, J. C., Lee, J. Y., Firme, M., Sinha, S. & Redfield, R. J. Extensive cotransformation of natural variation into chromosomes of naturally competent haemophilus influenzae. *G3: Genes, Genomes, Genetics* **4**, 717–731 (2014).

29. Power, J. J. *et al.* Adaptive evolution of hybrid bacteria by horizontal gene transfer. *Proceedings of the National Academy of Sciences* **118**, e2007873118 (2021).

30. McVean, G. A. A genealogical interpretation of linkage disequilibrium. *Genetics* **162**, 987–991 (2002).

31. Virtanen, P. *et al.* SciPy 1.0: Fundamental algorithms for scientific computing in python. *Nature Methods* **17**, 261–272 (2020).

32. Carattoli, A. *et al.* In silico detection and typing of plasmids using PlasmidFinder and plasmid multilocus sequence typing. *Antimicrobial agents and chemotherapy* **58**, 3895–3903 (2014).

33. Gillespie, J. J. *et al.* PATRIC: The comprehensive bacterial bioinformatics resource with a focus on human pathogenic species. *Infection and Immunity* **79**, 4286–4298 (2011).

34. Ashburner, M. *et al.* Gene ontology: Tool for the unification of biology. *Nature Genetics* **25**, 25–29 (2000).

35. Cantalapiedra, C. P., Hernández-Plaza, A., Letunic, I., Bork, P. & Huerta-Cepas, J. eggNOG-mapper v2: Functional annotation, orthology assignments, and domain prediction at the metagenomic scale. *Molecular biology and evolution* **38**, 5825–5829 (2021).

36. Voight, B. F., Kudaravalli, S., Wen, X. & Pritchard, J. K. A map of recent positive selection in the human genome. *PLoS biology* **4**, e72 (2006).

37. Tajima, F. Statistical method for testing the neutral mutation hypothesis by DNA polymorphism. *Genetics* **123**, 585–595 (1989).

38. Miles, A. *et al.* Cggh/scikit-allel: v1.3.13. (2024).

39. Marçais, G. *et al.* MUMmer4: A fast and versatile genome alignment system. *PLoS computational biology* **14**, e1005944 (2018).

40. Sonnhammer, E. L. & Durbin, R. A dot-matrix program with dynamic threshold control suited for genomic DNA and protein sequence analysis. *Gene* **167**, GC1–GC10 (1995).

41. Crits-Christoph, A., Olm, M. R., Diamond, S., Bouma-Gregson, K. & Banfield, J. F. Soil bacterial populations are shaped by recombination and gene-specific selection across a grassland meadow. *The ISME journal* **14**, 1834–1846 (2020).

42. Arevalo, P., VanInsberghe, D., Elsherbini, J., Gore, J. & Polz, M. F. A reverse ecology approach based on a biological definition of microbial populations. *Cell* **178**, 820–834 (2019).

43. Shen, E. *et al.* Subtyping analysis reveals new variants and accelerated evolution of clostridioides difficile toxin b. *Communications Biology* **3**, 347 (2020).

44. Mansfield, M. J. *et al.* Phylogenomics of 8,839 clostridioides difficile genomes reveals recombination-driven evolution and diversification of toxin a and b. *PLoS Pathogens* **16**, e1009181 (2020).

45. Steinberg, H. D. & Snitkin, E. S. Homologous recombination in clostridioides difficile mediates diversification of cell surface features and transport systems. *Msphere* **5**, 10–1128 (2020).

46. Dingle, K. E. *et al.* Recombinational switching of the clostridium difficile s-layer and a novel glycosylation gene cluster revealed by large-scale whole-genome sequencing. *The Journal of infectious diseases* **207**, 675–686 (2013).

47. Yahara, K. *et al.* The landscape of realized homologous recombination in pathogenic bacteria. *Molecular biology and evolution* **33**, 456–471 (2016).

48. Chun, S. & Fay, J. C. Evidence for hitchhiking of deleterious mutations within the human genome. *PLoS Genetics* **7**, e1002240 (2011).

49. Hartfield, M. & Otto, S. P. Recombination and hitchhiking of deleterious alleles. *Evolution* **65**, 2421–2434 (2011).

50. Desai, M. M., Walczak, A. M. & Fisher, D. S. Genetic diversity and the structure of genealogies in rapidly adapting populations. *Genetics* **193**, 565–585 (2013).

51. Steux, C. & Szpiech, Z. A. The maintenance of deleterious variation in wild chinese rhesus macaques. *bioRxiv* 2023–10 (2023).

52. Assaf, Z. J., Petrov, D. A. & Blundell, J. R. Obstruction of adaptation in diploids by recessive, strongly deleterious alleles. *Proceedings of the National Academy of Sciences* **112**, E2658–E2666 (2015).

53. Garud, N. R., Messer, P. W., Buzbas, E. O. & Petrov, D. A. Recent selective sweeps in north american drosophila melanogaster show signatures of soft sweeps. *PLoS Genetics* **11**, e1005004 (2015).

54. Karasov, T., Messer, P. W. & Petrov, D. A. Evidence that adaptation in drosophila is not limited by mutation at single sites. *PLoS Genetics* **6**, e1000924 (2010).

55. Schmidt, J. M. *et al.* Copy number variation and transposable elements feature in recent, ongoing adaptation at the Cyp6g1 locus. *PLoS Genetics* **6**, e1000998 (2010).

56. Magwire, M. M., Bayer, F., Webster, C. L., Cao, C. & Jiggins, F. M. Successive increases in the resistance of drosophila to viral infection through a transposon insertion followed by a duplication. *PLoS Genetics* **7**, e1002337 (2011).

57. Mackay, T. F. *et al.* The drosophila melanogaster genetic reference panel. *Nature* **482**, 173–178 (2012).

58. Lack, J. B. *et al.* The drosophila genome nexus: A population genomic resource of 623 drosophila melanogaster genomes, including 197 from a single ancestral range population. *Genetics* **199**, 1229–1241 (2015).

59. Harris, M. & Garud, N. R. Enrichment of hard sweeps on the x chromosome in drosophila melanogaster. *Molecular Biology and Evolution* **40**, msac268 (2023).

60. Vy, H. M. T., Won, Y.-J. & Kim, Y. Multiple modes of positive selection shaping the patterns of incomplete selective sweeps over african populations of drosophila melanogaster. *Molecular Biology and Evolution* **34**, 2792–2807 (2017).

61. Sheehan, S. & Song, Y. S. Deep learning for population genetic inference. *PLoS computational biology* **12**, e1004845 (2016).

62. Garud, N. R. & Petrov, D. A. Elevated linkage disequilibrium and signatures of soft sweeps are common in drosophila melanogaster. *Genetics* **203**, 863–880 (2016).
