# Supplementary material for: Gene-specific selective sweeps are pervasive across human gut microbiomes
Source: Nature. 2025 Dec 17;650(8102):710–7. doi: 10.1038/s41586-025-09798-y (PMC12916291; doi:10.1038/s41586-025-09798-y)
Supplement: Supplementary file 2 — Reporting Summary [file 41586_2025_9798_MOESM2_ESM.pdf]

nature portfolio

Corresponding author(s): 2024-01-00816A-Z

Last updated by author(s): 10/3/2025

Reporting Summary

Nature Portfolio wishes to improve the reproducibility of the work that we publish. This form provides structure for consistency and transparency in reporting. For further information on Nature Portfolio policies, see our Editorial Policies and the Editorial Policy Checklist.

Statistics

For all statistical analyses, confirm that the following items are present in the figure legend, table legend, main text, or Methods section.

n/a	Confirmed	
☐	☒	The exact sample size (*n*) for each experimental group/condition, given as a discrete number and unit of measurement
☒	☐	A statement on whether measurements were taken from distinct samples or whether the same sample was measured repeatedly
☐	☒	The statistical test(s) used AND whether they are one- or two-sided<br>*Only common tests should be described solely by name; describe more complex techniques in the Methods section.*
☒	☐	A description of all covariates tested
☐	☒	A description of any assumptions or corrections, such as tests of normality and adjustment for multiple comparisons
☐	☒	A full description of the statistical parameters including central tendency (e.g. means) or other basic estimates (e.g. regression coefficient) AND variation (e.g. standard deviation) or associated estimates of uncertainty (e.g. confidence intervals)
☐	☒	For null hypothesis testing, the test statistic (e.g. *F*, *t*, *r*) with confidence intervals, effect sizes, degrees of freedom and *P* value noted<br>*Give P values as exact values whenever suitable.*
☒	☐	For Bayesian analysis, information on the choice of priors and Markov chain Monte Carlo settings
☒	☐	For hierarchical and complex designs, identification of the appropriate level for tests and full reporting of outcomes
☐	☒	Estimates of effect sizes (e.g. Cohen's *d*, Pearson's *r*), indicating how they were calculated

Our web collection on statistics for biologists contains articles on many of the points above.

Software and code

Policy information about availability of computer code

Data collection	Data was collected by downloading from the internet using standard command line functions (wget)
Data analysis	This analysis relies extensively on custom python code, and uses several standard open-source libraries written in python (pandas, scipy, numpy, numba, and matplotlib). We also perform simulations of evolution using the software SLiM (version 4.0), which is also publicly available.<br><br>The software written for this paper can be accessed here: https://github.com/garudlab/iLDS

For manuscripts utilizing custom algorithms or software that are central to the research but not yet described in published literature, software must be made available to editors and reviewers. We strongly encourage code deposition in a community repository (e.g. GitHub). See the Nature Portfolio guidelines for submitting code & software for further information.

Data

Policy information about [availability of data](#)

All manuscripts must include a [data availability statement](#). This statement should provide the following information, where applicable:

- Accession codes, unique identifiers, or web links for publicly available datasets
- A description of any restrictions on data availability
- For clinical datasets or third party data, please ensure that the statement adheres to our [policy](#)

The data analyzed in this study is all publicly available.

Research involving human participants, their data, or biological material

Policy information about studies with [human participants or human data](#). See also policy information about [sex, gender (identity/presentation), and sexual orientation](#) and [race, ethnicity and racism](#).

Reporting on sex and gender	N/A
Reporting on race, ethnicity, or other socially relevant groupings	N/A
Population characteristics	N/A
Recruitment	N/A
Ethics oversight	N/A

Note that full information on the approval of the study protocol must also be provided in the manuscript.

Field-specific reporting

Please select the one below that is the best fit for your research. If you are not sure, read the appropriate sections before making your selection.

☐ Life sciences ☐ Behavioural & social sciences ☒ Ecological, evolutionary & environmental sciences

For a reference copy of the document with all sections, see [nature.com/documents/nr-reporting-summary-flat.pdf](#)

Ecological, evolutionary & environmental sciences study design

All studies must disclose on these points even when the disclosure is negative.

Study description	We used publicly available metagenomic data to identify selective sweeps across human gut microbiomes.
Research sample	We analyzed publicly available data from the Human Microbiome Project (HMP) (accession numbers PRJNA48479 and PRJNA275349), Xie et al (2016) (accession number PRJEB9576), Qin et al (2012) (accession number PRJNA422434), Korpela et al (2018) (accession number PRJEB24041), and the Unified Human Gastrointestinal Genome (http://ftp.ebi. ac.uk/pub/databases/ metagenomics/mgnify_genomes/). We used the publicly available Drosophila Genome Nexus data set (Lack et al. 2015), downloaded from www.johnpool.net.
Sampling strategy	In order to accurately identify common variants, we required at least 20 genomes/species for each species for which we attempted to identify selective sweeps.
Data collection	Data is all publicly available, and was downloaded from the internet.
Timing and spatial scale	HMP data was originally downloaded for a previous paper ~2017. Likewise, Drosophila data was downloaded for a previous paper by a member of our group in ~2022. UHGG data was downloaded in 2023.
Data exclusions	No data were excluded from analysis
Reproducibility	We (1) successfully recapitulated several identified positive controls (previously identified sweeps presented in other papers) in two pathogen species (H. pylori and C. difficile, most notably at the vacA and tcdB loci, respectively) as well as in Drosophila melanogaster (Ace, Chkov, and Cypg6 genes) compared sweeps identified. (2) We identified genes which were detected as under selection in the same species in multiple datasets, most notably at the mdxEF locus in the species R. bromii, which was detected as under selection in both the HMP dataset and the Westernized populations in the UHGG dataset

Randomization	One of the goals of our study is to identify differential selective signatures between groups (particularly Westernized vs. non-Westernized individuals). Therefore, it is not desirable to perform a random assignment of samples to groups.
Blinding	Blinding was not relevant to this study. All samples were analyzed in the same manner, and we were specifically interested in identifying differential signatures of evolution between known groups (Westernized vs. non-Westernized individuals).

Did the study involve field work? ☐ Yes ☒ No

Reporting for specific materials, systems and methods

We require information from authors about some types of materials, experimental systems and methods used in many studies. Here, indicate whether each material, system or method listed is relevant to your study. If you are not sure if a list item applies to your research, read the appropriate section before selecting a response.

Materials & experimental systems

n/a	Involved in the study
☒	☐ Antibodies
☒	☐ Eukaryotic cell lines
☒	☐ Palaeontology and archaeology
☒	☐ Animals and other organisms
☒	☐ Clinical data
☒	☐ Dual use research of concern
☒	☐ Plants

Methods

n/a	Involved in the study
☒	☐ ChIP-seq
☒	☐ Flow cytometry
☒	☐ MRI-based neuroimaging

Plants

Seed stocks	N/A
Novel plant genotypes	N/A
Authentication	N/A
